# *Epichloë*—the obligate mutualist protecting some commercially important temperate grasses from biotic and abiotic stresses

**DOI:** 10.3389/fmicb.2026.1878689

**Published:** 2026-08-11

**Authors:** John R. Caradus, Katrin G. Hewitt, David E. Hume, Wade J. Mace

**Affiliations:** 1 Grasslanz Technology Ltd., Hamilton, New Zealand; 2 Bioeconomy Science Institute, Hamilton, New Zealand; 3 Bioeconomy Science Institute, Palmerston North, New Zealand

**Keywords:** alkaloids, pasture persistence, perennial ryegrass, plant-insect interactions, temperate grasslands

## Abstract

The relationship between *Epichloë* fungal endophytes found in some temperate grasses spans the continuum from antagonistic to mutualistic. The predominant focus here is on those that form mutualistic associations with those temperate grasses of the New World due to their substantial economic impact. This review describes the evolutionary origin, discovery, lifestyle and life cycle of *Epichloë* as background to understand the close association between plant host and fungal endophyte. Many asexual *Epichloë* fungal endophytes are unique in being obligate mutualists, lacking a free-living stage in nature, and being maternally inherited through host seed. The compatibility between *Epichloë* and its natural host plant is so complete and specific that manually transferring an *Epichloë* endophyte strain to other grasses, even some that are closely related genetically, though possible, often results in endophyte and/or plant death or poorly performing plant genotypes. The stability of the association is facilitated through the biotic and abiotic protection of the host grass through the endophyte promoting endogenous defense responses in the host, or directly through the production of secondary metabolites. *Epichloë* endophytes are renowned for producing a diverse range of alkaloids that can negatively affect invertebrate and vertebrate herbivores, either through toxicity or feeding deterrence, thereby enhancing host survival. However, *Epichloë* chemistry can significantly impact the health and welfare of grazing animals resulting in large economic impacts. This includes effects known as ryegrass staggers, heat stress and fescue toxicosis. In some temperate areas of the world, notably New Zealand, Australia and USA, grasses such as perennial ryegrass and tall fescue need to have the *Epichloë*-derived protection to ensure persistence against invertebrate pests and drought but without the downsides of clinical and subclinical losses for grazing animals. Extensive screening has identified strains of *Epichloë* which have been successfully commercialised for ryegrasses and fescues that ensure persistence of the grass host without causing serious health and welfare impacts on the grazing animal. The development and deployment of selected *Epichloë* strains therefore represent a critical intersection of plant protection, animal welfare, and agricultural productivity, a theme explored throughout this review.

## 1 Background and discovery of *Epichloë*

Perennial ryegrass (*Lolium perenne* L.) and tall fescue (*Festuca arundinacea* Schreb.; *Schedonorus arundinaceus*) are temperate grass species of substantial economic importance due to their widespread use in grazed pasture systems throughout temperate regions globally (Hannaway et al., 1999; Stewart, 2006; Inda et al., 2014; Filho et al., 2023). *Lolium* is considered to have originated in the eastern Mediterranean region around 4.1 million years ago, with diversification occurring through autogamous (self-fertilisation) and allogamous (cross-fertilisation) lineages (Ghariani et al., 2020). Tall fescue is an allohexaploid species native to Europe and North Africa, with a probable hybrid origin, involving multiple morphotypes that suggest independent origins from different diploid progenitors (Hunt and Sleper, 1981; Hand et al., 2010; Cuyeu et al., 2013; Ghariani et al., 2020). Both of these grass species, now widely used in the New World (Stewart, 2006), co-evolved with the *Epichloë* fungus (Clavicipitaceae, Ascomycota) (Schardl et al., 1997; Schardl et al., 2008; Saikkonen et al., 2016; Bastías et al., 2024). *Epichloë* are biotrophs, requiring living host tissue to survive, grow, and reproduce, rather than simply killing the host upon infection (Florea et al., 2015). This mutualistic association provides *Epichloë* with a safe environment with adequate nutrients and water, while the grass host gains effective protection from both invertebrate and vertebrate herbivores (Schardl, 1996; Saikkonen et al., 2016; Realini et al., 2024), some foliar diseases (Pérez et al., 2020; Card et al., 2021; Fernando et al., 2022) as well as an enhanced tolerance to some abiotic stresses (Malinowski and Belesky, 2019; Hewitt et al., 2021). It has been described as a “defensive mutualism” in which the fungi defend their hosts against herbivory, thereby defending their own resources (Clay, 1988).

*Epichloë* was first noted over a century ago in *Lolium temulentum*, a grass known for its toxicity dating back to Roman times (Freeman, 1903). It is now known to contain *E. occultans* (Moon et al., 2000). However, the toxicity noted was most probably not due to *E. occultans*, as this is not known to contain any mammalian toxic alkaloids, but most likely due to the production of other mycotoxins, e.g., ergot in seedheads (*Claviceps purpurea*). A pathogenic and sexual form, *E. typhina*, was then noted in *Dactylis glomerata* with the potential to cause “choke” [i.e., maturation of the inflorescence is halted and no seeds are produced on that tiller (Kirby, 1961)] and identified as systemic, growing intercellularly and distributed by infected seed but also capable of producing fruiting bodies and spores (Sampson, 1933). The maternal inheritance for *Epichloë* was noted in *L. temulentum* and *L. perenne* over six seasons of seed production and the relationship was noted as not being obligate for the host species (Sampson, 1935). The endophyte in *L. perenne* was noted as being symptomless and for not producing fruiting bodies or “choke” (Sampson, 1937). Also, in the 1930s the fungus in *L. perenne* was suspected of causing staggers, an animal health neurotoxic disorder causing incoordination, and described in two papers in the early 1940s (Neill, 1940, 1941). However, it was not until the late 1970s and early 1980s that the significance of *Epichloë* in temperate grasses on animal health disorders, notably with tall fescue (fescue toxicosis; Bacon, 1995; Hoveland, 2009) and perennial ryegrass (ryegrass staggers first noted in 1906; Gilruth, 1906—referenced by Fletcher et al., 1999), was fully documented (Bacon et al., 1977; Fletcher and Harvey, 1981, Gallagher et al., 1981). The discovery in tall fescue in the USA was originally assigned as *E. typhina* (Bacon et al., 1977) (sexual form of *Epichloë*) but later confirmed as *E. coenophialum* (Morgan-Jones and Gams, 1982; Christensen and Latch, 1991) (asexual form of *Epichloë*). In New Zealand the discovery was made for the asexual form *E. festucae* var. *lolii* in perennial ryegrass (Hume et al., 2020; Card et al., 2024).

The aim of this review is to examine the biology and significance of *Epichloë* fungal endophytes in temperate grasses, and to highlight why these specialised microbes are unique and have been successfully commercialised as an economical and reliable biocontrol technology.

## 2 Nomenclature of *Epichloë*

What is now known as *Epichloë* was originally classified as two genera—*Neotyphodium* (formerly *Acremonium*) which included those species with no sexual life cycle (anamorphs) and *Epichloë* which included all those with a sexual life cycle (teleomorphs). However, nomenclatural rule changes in the International Code of Nomenclature for algae, fungi and plants, adopted at the 18th International Botanical Congress in Melbourne, Australia, in 2011, which required that a single name to be used for each fungal species, meant that *Neotyphodium* and *Epichloë* species were required to be brought into the one genus—in this case *Epichloë* (Leuchtmann et al., 2014).

## 3 The biology of *Epichloë*

While research on *Epichloë* has focused largely on its impact in perennial ryegrass and tall fescue it does occur in many other grass species and genera in the subfamily Pooideae (Clay, 1990; Leuchtmann, 1992; Schardl et al., 2004; Tadych et al., 2012) distributed through Europe, North and South America, Asia and Australasia (Tadych et al., 2014).

*Epichloë* species, and strains within some species, can vary from being a pathogen to a mutualist (White, 1988; Schardl, 1996; Campbell et al., 2017). Additionally, within a species of *Epichloë* there can be significant variation in the secondary metabolite profile among strains (Schardl, 2001).

### 3.1 Evolutionary origin

It has been proposed that “evidence of a significant tendency for co-divergence suggests that the origin of genus *Epichloë* may have been close in time to the origin of the highly speciose grass subfamily, Pooideae” (Saikkonen et al., 2016). Co-evolution of *Epichloë* and their grass host (Schardl et al., 2008) might suggest that the center of diversity and perhaps the region of origin of *Epichloë* was in the Northern Hemisphere (Eurasia or North America) based on ribosomal DNA diversity studies (White et al., 2001). However, other studies have concluded that some *Epichloë*-host phylogenies are incongruent and do not support codivergence as a characteristic of the association (Jackson, 2004; Ambrose et al., 2014).

The evolutionary origins of *Epichloë* remain uncertain, but it has been proposed that these fungi arose from plant pathogenic ancestors (Carroll, 1988; White, 1988; Saikkonen et al., 2004a). Repeat-induced point mutations, which are common in pathogenic fungi, have been identified in chromosome-level genome studies of two pathogenic *Epichloë* sibling species along with bipartite genome organisation (Treindl et al., 2021). This allows high evolutionary flexibility, enabling faster adaptation to new environments or hosts through reassortment of chromosome segments and integration of new genes as seen in other microbes (Sonnenberg and Haugen, 2023). Additionally, there have been examples of where saprophytic or pathogenic fungi have become asymptomatic endophytes due to a single genetic mutation (Freeman and Rodriguez, 1993) or perhaps as a result of phenotypic plasticity (Schulz and Boyle, 2005).

### 3.2 Lifestyle and life cycle

*Epichloë* hyphae are predominantly found in the above ground plant, both leaves and flowers, growing in intercellular spaces (Clay and Schardl, 2002; Saikkonen et al., 2016). Adventitious roots can also become infected as they start their development in the true stem below the meristematic zone (Christensen and Voisey, 2007). Growth of the endophyte is synchronised with that of the host at all stages of growth of the host grass (Christensen and Voisey, 2007). *Epichloë* exist asymptomatically at least for most of their life cycle within plant tissues (White, 1987; Leuchtmann and Clay, 1997). The hyphae are attached to the outside of cells (intercellular) and grow by intercalary extension (expansion of hyphae at locations other than the tips) as the leaf and flowering stem are growing and extending (Christensen et al., 2008; Voisey, 2010). The growth of host plant and endophyte hyphae appear to be synchronised, with hyphae seldom branching and predominantly growing parallel to the leaf axis (Christensen et al., 2002; Liu et al., 2017). While this is a notable feature of *Epichloë* hyphal growth which is crucial for forming a structured hyphal network within the host plant, contributing to the mutualistic relationship and allowing maternal inheritance, it is not unique and has been documented in other filamentous fungi (Read, 2011). Intercalary growth of hypha is supported by specific genetic and biochemical pathways, such as the cell-wall integrity mitogen-activated protein kinase cascade in *Epichloë* (Becker et al., 2015), which regulates hyphal network formation (Lowry et al., 2010). In endophytic *Epichloë*, chitin is absent from hyphal cell walls and replaced by chitosan via the action of chitin deacetylase. Chitin is, however, retained in septa, where it facilitates intercalary hyphal growth and may contribute to evasion of host immune responses (Noorifar et al., 2021). Chitosan is a poor substrate for extracellular plant chitinases (Ride and Barber, 1990; Pusztahelyi, 2018). However, chitin is present when the fungus is grown in axenic culture (Scott et al., 2018) or epiphytically (Noorifar et al., 2021) acting as a protective barrier (Lenardon et al., 2010).

The *Epichloë* species can be either sexually and/or asexually transmitted (Moon et al., 2004; Tadych et al., 2014; Berry et al., 2022). Sexual reproduction of *Epichloë* species provides the ability for horizontal transmission of the fungus from one individual host plant to another unrelated plant. Interestingly, where an *Epichloë* strain can be transmitted both asexually and sexually on an individual plant, it seems likely that this duality of reproductive processes is likely triggered by an epigenetic effect (Saikkonen et al., 2016). A taxonomic revision of *Epichloë* genus, now over a decade old, determined that of the 43 distinct lineages (i.e., 34 distinct species, 3 subspecies, and 6 varieties), 25 were anamorphic (asexual and reproduce by conidia rather than sexual ascospores) and 18 teleomorphic (produces ascospores through meiosis), with only 3 reproduced only sexually with only horizontal transmission, 28 only asexually and 12 species/subspecies both sexually and asexually (Leuchtmann et al., 2014; Table 1). Since then, several new species of *Epichloë* have been identified including *E. alsodes, E. hybrida, E. novae-zelandiae, E. scottii, E. transsilvanica* (Table 1) and unnamed species such as the *Epichloë* recently identified in *Festuca simensis*, a grass of highland tropical Uganda, Africa (Card et al., 2025).

**TABLE 1:** Characteristics of *Epichloë* species, including host association, region of origin, interspecific hybrid status, reproductive mode, and transmission pathway to another host.

*Epichloë* species	Host grass genus	Region of origin	Hybrid status	Sexual and/or asexual reproduction*	Ability to reproduce sexually	Form of transmission
*E. alsodes†*	*Poa*	North America	Hybrid	Asexual	Not observed	Vertical
*E. amarillans*	*Ammophila, Agrostis, Hordeum, Sphenopholis*	North and South America, Europe, North Africa	Non-hybrid	Sexual	Yes	Vertical
***E. aotearoae***	*Echinopogon*	New Zealand	Non-hybrid	Asexual	Not observed	Vertical
***E. australiensis***	*Dichelachne, Echinopogon*	New Zealand, Australia	Hybrid	Asexual	Not observed	Vertical
*E. baconii*	*Agrostis*	Europe, North Africa	Non-hybrid	Sexual	Yes	Horizontal
*E. brachyelytri*	*Brachyelytrum*	Europe, North Africa, North America	Non-hybrid	Sexual	Yes	Vertical
*E. bromicola*	*Agropyron, Brachypodium, Bromus, Elymus, Leymus, Roegneria*	Europe, North Africa, Asia, North and South America	Non-hybrid	Sexual	Yes, in some host species	Vertical and horizontal
*E. cabralii*	*Bromus, Phleum*	Europe, North Africa, Asia, North America and South America	Hybrid	Asexual	Not observed	Vertical
*E. canadensis*	*Elymus*	Europe, North Africa, Asia, North America	Hybrid	Asexual	Not observed	Vertical
***E. chisosa***	*Achnatherum*	Asia	Hybrid	Asexual	Not observed	Vertical
***E. coenophialum***	*Festuca*	Europe, North Africa, Asia, North and South America	Hybrid	Asexual	Not observed	Vertical
*E. danica*	*Hordelymus*	Europe, North Africa	Hybrid	Asexual	Not observed	Vertical
*E. disjuncta*	*Hordelymus*	Europe, North Africa	Hybrid	Asexual	Not observed	Vertical
*E. elymi*	*Elymus*	Europe, North Africa, Asia, North America	Non-hybrid	Sexual	Yes	Vertical
*E. festucae*	*Fescue*	Europe, North Africa, Asia, North and South America	Non-hybrid	Sexual	Yes	Vertical
*E. festucae* var. ***lolii***	*Lolium*	Europe, North Africa	Non-hybrid	Sexual	Not observed	Vertical
***E. funkii***	*Achnatherum*	Asia	Hybrid	Asexual	Not observed	Vertical
***E. gansuensis***	*Achnatherum*	Asia	Non-hybrid	Asexual	Not observed	Vertical
***E. gansuensis* var. *inebrians***	*Achnatherum*	Asia	Non-hybrid	Asexual	Not observed	Vertical
*E. glyceriae*	*Glyceria*	Europe, North Africa, North America	Non-hybrid	Sexual	Yes	Horizontal
***E. guerinii***	*Melica*	South America, Sub-Saharan Africa	Hybrid	Asexual	Not observed	Vertical
*E. hordelymi*	*Hordelymus*	Europe, North Africa	Hybrid	Asexual	Not observed	Vertical
*E. hybrida†*	*Lolium*	Europe	Hybrid	Asexual	Not observed	Vertical
*E. liyangensis*	*Poa*	Europe, North Africa, Asia, North and South America, New Zealand	Hybrid	Sexual	Yes	Vertical
***E. melicicola***	*Melica*	South America, Sub-Saharan, Africa, South America	Hybrid	Asexual	Not observed	Vertical
***E. mollis***	*Holcus*	Europe, North Africa	Non-hybrid	Asexual	Yes	Vertical
***E. novae-zelandiae†***	*Poa*	New Zealand	Hybrid	Not observed		
***E. occultans***	*Lolium*	Europe, North Africa	Hybrid	Asexual	Not observed	Vertical
***E. pampeana***	*Bromus*	Europe, North Africa, Asia, North and South America	Hybrid	Asexual	Not observed	Vertical
***E. schardlii***	*Cinna*	North America	Hybrid	Asexual	Not observed	Vertical
*E. scottii* var. *nov.*†	*Melica*	Europe	Non-hybrid	Sexual	Not observed	Vertical and possibly horizontal
***E. sibirica***	*Achnatherum*	Asia	Non-hybrid	Asexual	Not observed	Vertical
***E. siegelii***	*Fescue*	Europe, North Africa, Asia, North and South America	Hybrid	Asexual	Not observed	Vertical
***E. sinica***	*Roegneria*	Asia	Hybrid	Asexual	Not observed	Vertical
***E. sinofestucae***	*Fescue*	Europe, North Africa, Asia, North and South America	Hybrid	Asexual	Not observed	Vertical
***E. stromatolonga***	*Calamagrostis*	Asia	Non-hybrid	Asexual	Not observed	Vertical
*E. sylvatica*	*Brachypodium*	Europe, North Africa, Asia	Non-hybrid	Sexual	Yes	Vertical
*E. sylvatica* subsp. *polliinensis*	*Hordelymus*	Europe, North Africa	Non-hybrid	Sexual	Yes	Vertical
***E. tembladerae***	*Briza. Bromus, Fescue, Hordeum, Melica, Phleum. Poa*	Europe, North Africa, Asia, North and South America, Sub-Saharan Africa, New Zealand	Hybrid	Asexual	Not observed	Vertical
*E. typhina*	*Anthoxanthum, Brachypodium, Bromus, Dactylis, Lolium, Phleum*	Europe, North Africa, Asia, North and South America	Non-hybrid	Sexual	Yes, in some host species	Vertical and horizontal
*E. typhina* var. ***ammophilae***	*Ammophila*	North America	Non-hybrid	Sexual	Not observed	Not observed
*E. typhina* sub sp. *clarkii*	*Holcus*	Europe, North Africa	Non-hybrid	Sexual	Yes	Horizontal
*E. typhina* sub sp. *poae*	*Poa*	Europe, North Africa, Asia North and South America, New Zealand	Non-hybrid	Sexual	Yes, in some host species	Vertical and horizontal
*E. typhina* sub sp. *poae* var. *aonikenkana*	*Bromus*	Europe, North Africa, Asia, North and South America	Non-hybrid	Sexual	Not observed	Vertical
*E. typhina* sub sp. *poae* var. ***canariensis***	*Lolium*	Europe, North Africa	Non-hybrid	Sexual	Not observed	Vertical
*E. typhina* sub sp. *poae* var. ***huerfana***	*Fescue*	Europe, North Africa, Asia, North and South America	Non-hybrid	Sexual	Not observed	Vertical
***E. uncinata***	*Fescue*	Europe, North Africa, Asia, North and South America	Hybrid	Asexual	Not observed	Vertical

*Epichloë* species in bold were previously classified as *Neotyphodium*. Information derived primarily from Leuchtmann et al. (2014), Caradus et al. (2021b) and Newman et al. (2021). Vertical transmission refers to the inheritance of *Epichloë* endophytes from parent to offspring via host seed, whereas horizontal transmission involves the spread of the fungus between host plants through infectious spores.

* Sexual indicates teleomorphic type and asexual indicates anamorphic type. †Discovered after publication of, or not described in Leuchtmann et al. (2014)—refer either Moon et al. (2007), Leuchtmann et al. (2019), Campbell et al. (2017), Shymanovich et al. (2017), or Thünen et al. (2022).

**FIGURE 1:**
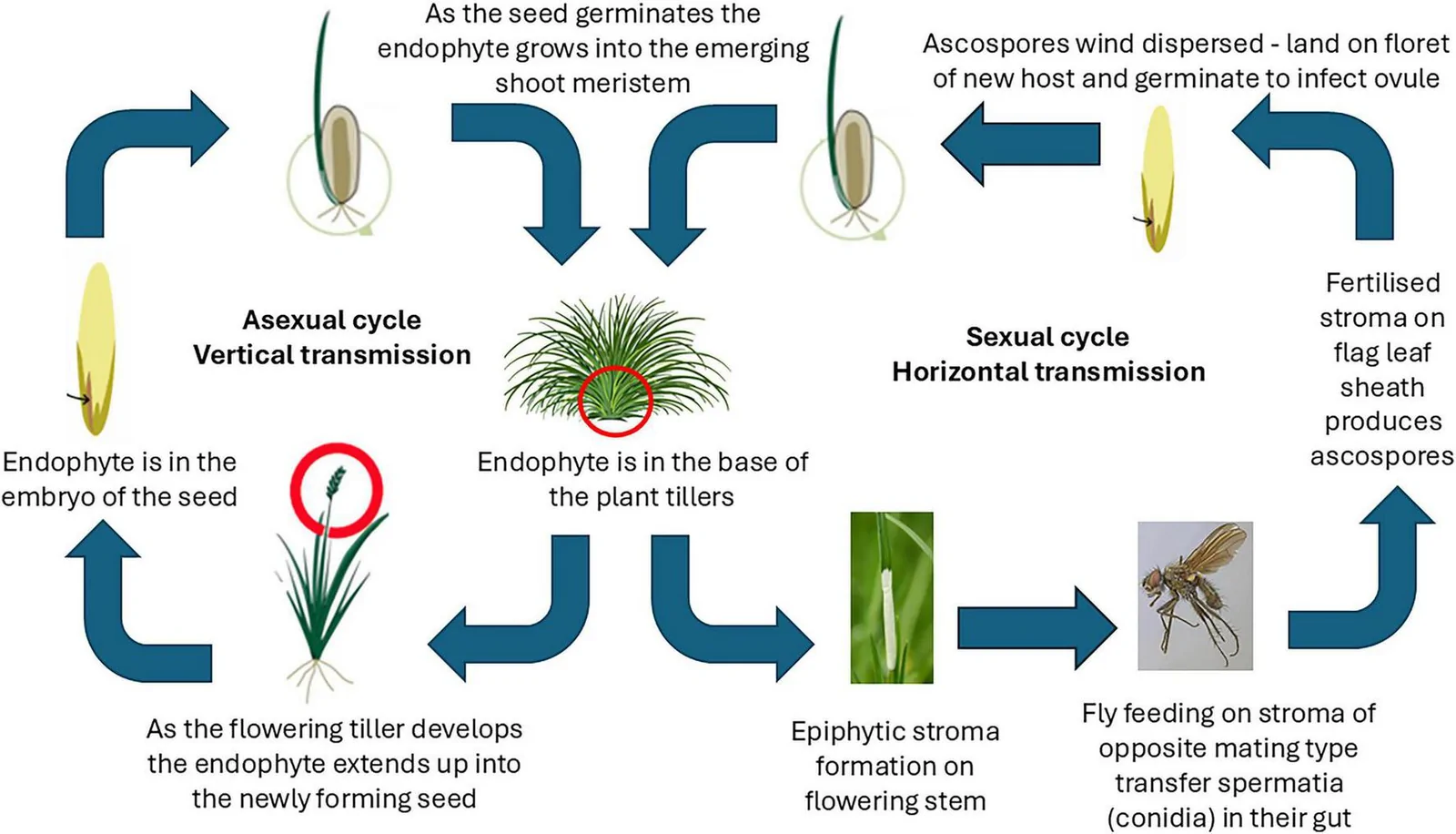
Asexual and sexual life cycles of *Epichloë*. A fly from the genus *Botanophila* is often involved in transferring conidia to the opposite mating type. The fungal stromata are visited by *Botanophila* flies for feeding and egg laying (Kohlmeyer and Kohlmeyer, 1974; Bultman and Leuchtmann, 2008; Bultman et al., 2022) which are attracted by the volatile compounds—methyl (Z)-3-methyldodec-2-enoate and chokol K (Steinebrunner et al., 2008b).

The rare occurrence of sexually reproducing strains had been previously observed (Meijer and Leuchtmann, 1999). For asexual *Epichloë* species it has been categorically shown that they are solely maternally inherited (vertical transmission) and do not travel via pollen (Liu et al., 2017; Forester et al., 2025). It is generally accepted that *Epichloë* species that are interspecific hybrids, which have heteroploid (aneuploid or polyploid) genomes, lack the capacity for sexual reproduction and are vertically transmitted through seed (Clay and Schardl, 2002). In contrast, those that are horizontally (contagiously) transmitted to other hosts through ascospore production are haploids (Schardl, 2001; Moon et al., 2004; Figure 1). In pre-anthesis florets, vertically transmitted *Epichloë* hyphae grow throughout the ovule nucellus, but not the integument or embryo sac, with a single hypha extending from the ovary placenta into the ovule which when fertilised extends into the developing seed and embryo (Liu et al., 2017).

It has been concluded that “the abundance of hybrid species among grass endophytes, and their prevalence in many host populations suggests a selective advantage of hybridisation for the mutualistic endophytes” (Moon et al., 2004). Hybridisation of *Epichloë* endophytes has been proposed as a mechanism that may increase host competitive potential in stressful, resource-limited environments (Saari and Faeth, 2012). However, a consideration that it may have been due to their greater genetic variation through combining genomes from different species, which can then lead to increased adaptability and fitness benefits for host plants possibly through producing a wider range of bioactive alkaloids (Tsai et al., 1994; Gentile et al., 2005; Charlton et al., 2012; Schardl et al., 2013b) does not necessarily appear to be the case (Oberhofer et al., 2014; Jia et al., 2016). It is possible that *Epichloë* strain by host species compatibility may compromise these types of comparisons. It has been convincingly argued, supported by modeling, that *Epichloë* species that are strictly vertically transmitted through seed can only function as mutualists, as persistence in the environment would be unlikely without conferring some benefit to the host (Newman et al., 2021). That is despite others proposing that vertically transmitted endophytes are “usually parasitic” (Faeth and Sullivan, 2003) or fall along a mutualism-parasitism continuum (Müller and Krauss, 2005). However, it has also been considered that “in mutualistic associations, where one organism is contained within another organism, selection against sexual reproduction of the endobiont frequently occurs” (White, 1988; referring to Law, 1985). There is no doubt that complex and dynamic processes underlie grass-endophyte symbioses (Charlton et al., 2014).

Hybridisation of *Epichloë* species could potentially occur either during sexual reproduction or through vegetative nuclear fusion between two *Epichloë* species growing with the same host (Selosse and Schardl, 2007). Both options seem to be problematic. Sexual hybridisation between different *Epichloë* species was unsuccessful from many attempts with only one generated hybrid which still only produced haploid offspring which had to be rescued from the stroma since ascospores did not eject (Schardl and Leuchtmann, 1999). Most *Epichloë* species are not interfertile, an exception being *E. typhina* and *E. clarkii* (presumably being *E. typhina* sub *clarkii* (Craven et al., 2001; Table 1) which can hybridise (Bultman et al., 2011; Treindl and Leuchtmann, 2019). However, even here when ascospores were collected from both species, only 9.3% of fungal fruiting bodies contained spores of interspecific hybrid origin, suggesting that postzygotic isolating mechanisms provide an important means of reproductive isolation for *Epichloë* species (Bultman et al., 2011). It has also been considered that if selection against hybrids is strong then prezygotic reproductive barriers are likely reinforcing this outcome (Servedio and Noor, 2003). Additionally, sexual reproduction of *Epichloë* involves stroma formation which prevents host flowering (a disease referred to as choke; Western and Cavett, 1959; Kohlmeyer and Kohlmeyer, 1974; Merlet et al., 2022) whereas asexual reproduction through vertical transmission in host seeds does not affect host fitness (Leuchtmann and Schardl, 1998).

Hybridisation via vegetative nuclear fusion (Schardl and Craven, 2003) is equally challenging. In nature it is most unusual for more than one *Epichloë* strain to infect a single grass genotype, and generally competitive exclusion will eliminate one quite quickly (Christensen et al., 2000; Wille et al., 2002). However, grove bluegrass (*Poa alsodes*), a North American woodland grass, can independently host two species of *Epichloë* that vary in alkaloids produced (Shymanovich et al., 2019). It is therefore hypothesised that “hybridisation events must occur shortly after coinfection” (Selosse and Schardl, 2007). However, Selosse and Schardl (2007) noted that many hybrid *Epichloë* strains are found in grass species that are quite distantly related to all of their parents’ known hosts (Chung et al., 1997; Moon et al., 2004; Gentile et al., 2005), and that *Epichloë* strains manually inoculated into a non-host species often cause a defense response leading to spontaneous endophyte extinction after inoculation or plant death (Koga et al., 1993; Christensen, 1995; Christensen et al., 2000).

Mating of sexual *Epichloë* occurs due to the formation of stroma—a structure that proliferates over the plant host surface. For mating to occur spermatia (conidia) of the opposite mating type within the same mating population can be transferred by a specific fly of the genus *Botanophila* (Bultman et al., 2000; Bultman and Leuchtmann, 2008; Bultman et al., 2022; Figure 1). However, it has been noted that this interaction with flies is not obligatory, and that fertilisation of the fungus can also occur without the involvement of flies (Rao and Baumann, 2004; Górzyńska et al., 2011). Fertilisation leads to proliferation of thick mycelium over the stroma surface (Schardl and Tsai, 1993; Leuchtmann et al., 1994). The proliferating mycelium appears to be from spermatia or stroma and not the result of heterokaryosis (Chung and Schardl, 1997) where the mycelium would contain two or more genetically distinct nuclei within a single common cytoplasm. However, formation of heterokaryons both within and between *Epichloë* species is possible when grown on culture media as demonstrated by Chung and Schardl (1997). Despite that it is considered that heterokaryosis occurs so rarely for *Epichloë* species that it has not been selected against. Similarly, though multiple *Epichloë* strain infection of individual plants probably does occur (Schardl et al., 1994) they are rare (Meijer and Leuchtmann, 1999) except when in sexually reproducing forms. This may be modulated by the genetic variation of both host and endophyte (Wille et al., 2002).

Recently, the bacterial sexual parasite *Wolbachia* has been shown to infect 70% of fly larvae emanating from eggs laid by *Botanophila* flies resulting in larval death which could have far reaching effects on not only the fly host, but also the *Epichloë* fungi (Pagel et al., 2019). Infection by *Wolbachia* could therefore limit consumption of *Epichloë* spores by *Botanophila* larvae if the bacteria promoted premature larval death. Additionally, the mycoparasite *Clonostachys epichloë*, also has entomopathogenic activity toward *Botanophila* flies involved in the sexual cycle of the *Epichloë* and can colonise the surface of grass seeds infected with the *Epichloë* endophyte (Górzyńska, 2025).

Though *Epichloë* are biotrophs, it is possible to culture some species/strains on defined media, which is a core requirement for transferring asexual species/strains to novel hosts (Latch and Christensen, 1985). When isolated from the host plant, *Epichloë* generally grow slowly on defined media (Chung and Schardl, 1997).

### 3.3 Host—strain specificity

The genetic compatibility of an *Epichloë* strain with a host grass can be very specific, such that moving *Epichloë* strains across taxa can be challenging and often results in poor host plant growth and performance (Song H. et al., 2015; Saikkonen et al., 2016) and also resulting in different levels of alkaloid production than the original host and poorer viability in stored seed (Hennessy et al., 2016; Freitas, 2017). This effect became evident when infecting modern cereals such as rye (*Secale cereale*) and wheat (*Triticum aestivum*) with *Epichloë* strains isolated from wild grass relatives (Simpson et al., 2014) such as *Elymus* and *Hordeum* species (Card et al., 2014). It was concluded that “the formation of synthetic symbioses is possible but that issues of compatibility require resolution” with resulting plant phenotypes ranging from heavily stunted (sometimes terminal) through to some that resemble healthy uninfected plants (Simpson et al., 2014). This close strain to host specificity is possibly a result of their co-evolution (Saikkonen et al., 2004b; Easton, 2007; Schardl et al., 2008; Gundel et al., 2010). Genes in *Epichloë* encoding for secreted proteins have been identified as key determinants of host specialisation of endophyte strains (Schirrmann et al., 2018). Despite the general principle of close strain to host specificity there are examples of where some *Epichloë* strains are shared between different *Elymus* species and even across continents (Schirrmann et al., 2015).

### 3.4 Chemistry

The association of mutual benefit between asexual *Epichloë* and host grasses is facilitated by the large range of secondary metabolites and specifically alkaloids produced by the endophyte when located *in planta* (Bush et al., 1997; Johnson et al., 2019; Lee et al., 2021). Secondary metabolites are organic compounds produced by organisms that are not directly involved in their growth, development, or reproduction (Zandavar and Afshari Babazad, 2023). They are associated with defense, signaling, and environmental adaptation, improving ecological fitness but do not directly impact growth (Bharadwaj et al., 2020).

The impact of *Epichloë* in improving plant defenses is due not only to produced secondary metabolites, but also through these endophytes enhancing plant immunity against chewing insects by promoting endogenous defense responses mediated by the jasmonic acid pathway (Bastías et al., 2017a). Additionally, endophytes have been shown to influence quantitative changes in antioxidant phenolic composition (Qawasmeh et al., 2012a; Qawasmeh et al., 2012b). Some phenolic-like compounds have been shown to be exuded into the rhizosphere resulting in improved phosphorus uptake in low phosphorus environments (Malinowski and Belesky, 2000).

#### 3.4.1 Diversity of secondary metabolites

The secondary metabolite chemistry of *Epichloë* is only partly understood and includes both alkaloids and non-alkaloids.

##### 3.4.1.1 Alkaloids

**FIGURE 2:**
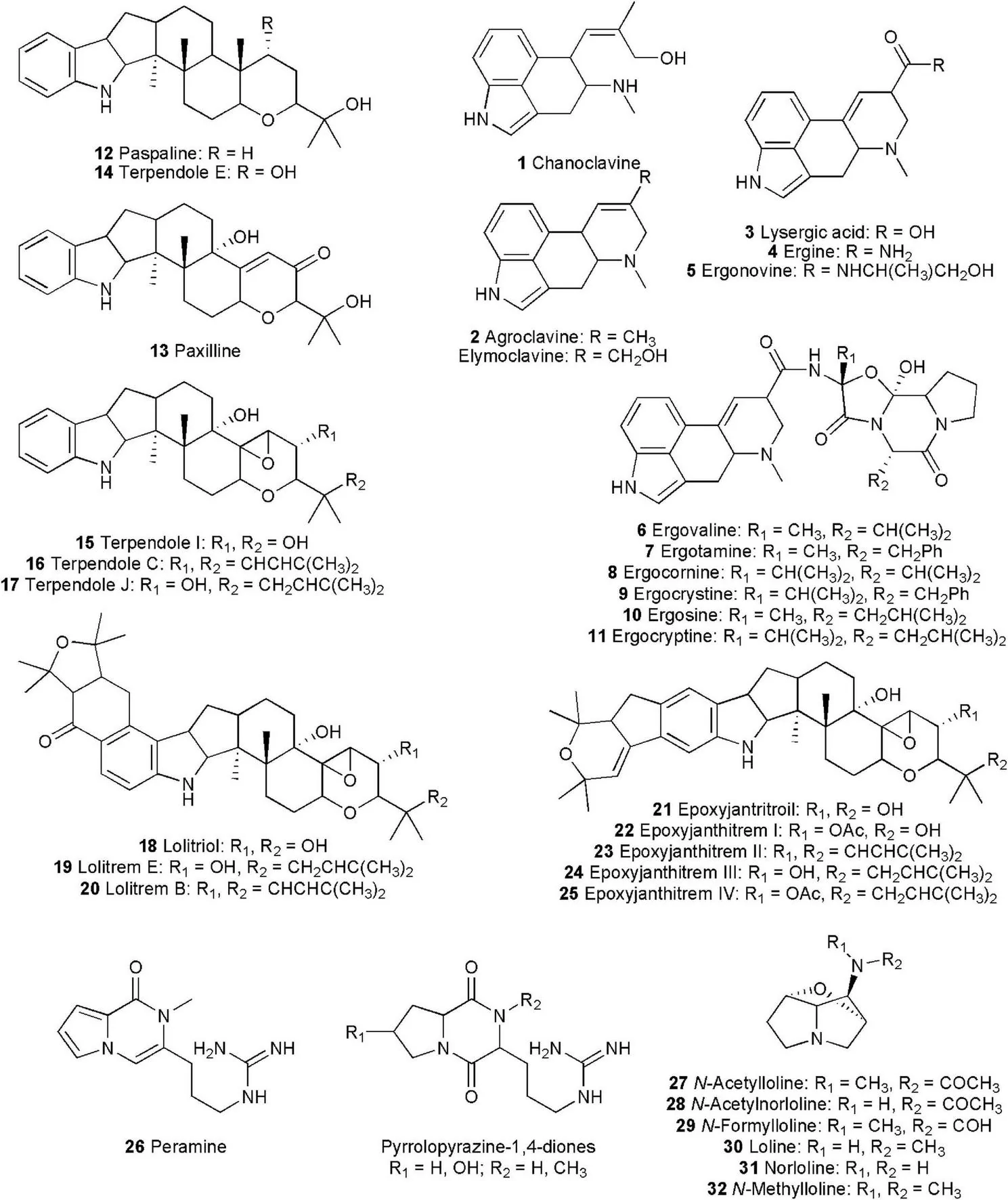
Chemical structures of known alkaloids produced by *Epichloë* in host grasses.

An alkaloid is an organic compound containing a nitrogen atom, typically in a heterocyclic ring (Bhambhani et al., 2021). Four major classes of alkaloids are known to be produced by *Epichloë*: ergot alkaloids, indole diterpenes, pyrrolopyrazines, and aminopyrrolizidines (Siegel and Bush, 1996; Schardl et al., 2012; Schardl et al., 2013a; Schardl et al., 2013b; Panaccione et al., 2014). The chemical structures of compounds representing the different alkaloids expressed by *Epichloë* are shown in Figure 2. A description of the main categories of known alkaloids associated with *Epichloë* is summarised below.

1. *Ergot alkaloids:* This extensive group of nitrogenous compounds derived from L-tryptophan has four main groups of compounds—clavines [e.g., chanoclavine **(1)**, agroclavine **(2)**], lysergic acid **(3)**, lysergic acid amides [e.g., ergine **(4)**, ergonovine **(5)**] and ergopeptines [e.g., ergovaline **(6)**, ergotamine **(7)**, ergocornine **(8)**, ergocristine **(9)**, ergosine **(10)**, ergocryptine **(11)**] (Schardl et al., 2006; Gerhards et al., 2014; Guerre, 2015). During the evolutionary development of *Epichloë* this diversity of ergot alkaloids has resulted from gene gains, gene losses, and gene sequence changes (Young et al., 2015). Ergovaline is the most prevalent and toxic ergot alkaloid expressed by *Epichloë* infected grasses (Lyons et al., 1986; Schardl et al., 2009; Caradus et al., 2020), but other ergot alkaloids produced by *Epichloë* endophytes can also contribute to animal health and welfare issues (Gadberry et al., 2003; Duringer et al., 2007; Guerre, 2015). The main animal health and welfare concerns associated with ergot alkaloid consumption are summarised in Table 2. Ergovaline has low *in-planta* mobility and strong regulation of accumulation by the internal plant environment (Spiering et al., 2002) and found principally in the basal tissues of tillers (Spiering et al., 2005). Clavines are the simplest compounds of the ergot-alkaloid class produced by the early steps of the ergot-alkaloid biosynthetic pathway (Young et al., 2015). Chanoclavine, although an intermediate compound in the ergot alkaloid pathway, can accumulate to be at a similar concentration as the ergopeptine alkaloids. However, chanoclavine is not known to cause any animal toxicity issues (Finch et al., 2019) but may have potential insecticidal activity although this is not confirmed (Hudson et al., 2021). Lysergic acid which is metabolised from elymoclavine however had been associated with high levels of African black beetle (*Heteronychus arator*) deterrence (Hudson et al., 2021), although no apparent effect on fall army worm (*Spodoptera frugiperda*) (Clay and Cheplick, 1989).

**TABLE 2:** Animal health and welfare disorders associated with consuming *Epichloë* infected grasses.

Animal health disorder	*Epichloë* species	Potential associated alkaloids	References
Ruminants			
Fescue toxicosis in cattle—causes tail loss, decreased feed intake, abortions, lameness due to sloughing off or loss of hooves, rough hair coats, reduction in calving rate, resulting in production losses, e.g., reduced weight gains, reduced milk production in dairy cows	*E. coenophiala*	Ergovaline and to a lesser extent lysergic acid, primarily acting as a vasoconstrictor	Jacobson et al., 1970; Porter et al., 1979; Hoveland et al., 1983; Hemken et al., 1984; Bacon et al., 1986; Stuedemann and Hoveland, 1988; Porter and Thompson, 1992; Roberts and Andrae, 2004; Klotz et al., 2006, 2008; Strickland et al., 2009; Tor-Agbidye et al., 2001; Guerre, 2015; Hume et al., 2025
Fescue toxicosis in deer—causing abdominal fat accumulation particularly in females	*E. coenophiala*	Ergovaline	Wolfe et al., 1998
Heat stress in ryegrasses	*E. festucae* var. *lolii*	Ergovaline	Fletcher et al., 1994; Fletcher et al., 2017
Ryegrass staggers, resulting in reduced milk production in dairy cows and lower liveweight gain, greater faecal soiling in the breech area (termed “dags”), higher incidence of myiasis (flystrike), compromised reproductive performance and increased mortality	*E. festucae* var. *lolii*	Lolitrem B	Fletcher and Harvey, 1981; Gallagher et al., 1981; Gallagher et al., 1982; Gallagher et al., 1984; Mortimer et al., 1984; Smith and Julian, 1998; Tor-Agbidye et al., 2001; Duringer et al., 2021; Philippe, 2016; Fletcher et al., 2017; Hume et al., 2025
	*E. festucae* var. *lolii*	Lolitrem A	Munday-Finch et al., 1995
Ryegrass staggers	*Epichloë* sp. LpTG-3	Epoxyjanthitrems	Babu, 2009; Babu et al., 2018; Finch et al., 2020
Ryegrass staggers (mild)	*E. festucae* var. *lolii*	Paxilline and terpendole C	Gallagher and Hawkes, 1986; Vassiliadis et al., 2023
Perennial ryegrass toxicosis—noted in Australia	*E. festucae* var. *lolii*	Ergovaline and lolitrem B	van Heeswijck and McDonald, 1992; Reed, 2005; Reed et al., 2016
“Huecu” toxicosis, a staggers-like syndrome	*E. tembladerae*	Indole-diterpenoid tremorgens other than lolitrem B; and glycoproteins	Miles et al., 1995; Cabral et al., 1999
Drunken horse grass poisoning	*E. gansuense*	Ergonovine and ergonovinine	Zhang et al., 2014
Pseudoruminants			
Ryegrass staggers, brain oedema, degenerative renal and hepatic lesions in camels	*E. festucae* var. *lolii*	Lolitrem B (and possible ergovaline)—in ryegrass hay and straw	Alabdouli et al., 2014
Staggers in alpacas, along with subclinical effects—heat sensitivity and ill-thrift and reduced fertility	*E. festucae* var. *lolii*	Lolitrem B and ergovaline	Sampaio et al., 2008
Non-ruminants			
Abortions in pregnant horses—increased gestation lengths, agalactia, foal and mare mortality, tough and thickened placentas, weak and dysmature foals	*E. festucae*	Ergovaline	Cross, 1997
Ryegrass staggers in a range of non-ruminants	*E. festucae* var. *lolii*	Lolitrem B	Hume et al., 2016; Zebeli et al., 2026
Horse oedema	*E. coenophiala* in Mediterranean-type tall fescue	Speculated to be loline, but essentially unknown	Bourke et al., 2009; Finch et al., 2017
White rhinoceros (*Ceratotherium simum*)	*E. festucae* var. *lolii*	Lolitrem B	Bluett et al., 2004

More detailed impacts of *Epichloë* alkaloids on animal heath symptoms are summarised by Caradus and Johnson (2020).

2. *Indole diterpenoids*: Indole diterpenoids are a structurally diverse group of compounds with a common core structure of a cyclic diterpene skeleton (derived from geranylgeranyl diphosphate), and an indole structure derived from tryptophan (Saikia et al., 2006). They include the compounds paspaline (**12**), paxilline (**13**), terpendoles (**14**–**17**), lolitriol (**18**), lolitrems (**19**, **20**), and epoxyjanthitrems (**21**–**25**) (Weedon and Mantle, 1987; Munday-Finch et al., 1997; Munday-Finch et al., 1998; Gatenby et al., 1999; Tapper and Lane, 2004; Finch et al., 2020). Some lolitrem compounds are neurotoxins and cause tremorgens or staggers in livestock (Gallagher et al., 1984; Philippe, 2016). However, not all lolitrem compounds are tremorgenic—for example lolitrem E (**19**) the precursor of lolitrem B (**20**) is noted as not being tremorgenic when fed to mice (Miles et al., 1994). Lolitrem B, which is tremorgenic, has highest concentration in seed, the base of tillers and then in older leaf sheaths and dead leaves (Ball et al., 1997b; Spiering et al., 2005).

3. *Pyrrolopyrazines*: Peramine (**26**) is a pyrrolopyrazine-1-one metabolite (Rowan et al., 1986) found widely across *Epichloë* species but not outside of the epichloae (Siegel et al., 1990; Panaccione et al., 2014; Tanaka et al., 2005). Peramine is the product of a single multifunctional enzyme (PerA) as opposed to a complex pathway (Tanaka et al., 2005). Natural mutations and truncations of the PerA enzyme have been shown to produce a range of pyrrolopyrazine-1,4-dione and pyrrolopyrazine-1-one derivatives (Berry et al., 2019). Peramine is water-soluble and is distributed throughout the host plant, including tissues remote from fungal hyphae (Ball et al., 1997a; Spiering et al., 2002, 2005). Its detection in cut leaf fluid and guttation fluid suggests that peramine is transported within the plant, potentially via the xylem (Koulman et al., 2007). Peramine has no known negative effects on vertebrates (Rowan, 1993), although it has been speculated to be associated with high fecal moisture with lambs, and consequent fecal soiling (Pownall et al., 1993).

4. *Lolines (1-aminopyrrolizidines)*: Three main loline derivatives can be found in *Epichloë* infected grasses—*N*-acetylloline (NAL **27**), *N*-acetylnorloline (NANL **28**) and *N*-formylloline (NFL **29**) (Schardl et al., 2012). Trace amounts of loline (**30**), norloline (**31**) and *N*-methylloline (**32**) have also been observed in endophyte infected meadow fescue (*Festuca pratensis*) (Justus et al., 1997; Pan et al., 2014). Lolines are found in high concentrations in tall fescue and even higher levels in meadow fescue, also in some other *Epichloë* infected grasses (Schardl et al., 2007; Schardl et al., 2012), but not in perennial ryegrass infected with *E. festucae* var. *lolii* (Siegel et al., 1990; Ball and Tapper, 1999). In meadow and tall fescue, evidence from insect bioassays indicates that loline alkaloids are translocated in, or readily accessible from, the phloem (Popay et al., 2023) and translocated to the root system (Patchett et al., 2008b; Patchett et al., 2011b). Lolines have also been observed at detectable levels in guttation fluid (Koulman et al., 2007). Lolines appear to have no negative effects on vertebrates (Schardl et al., 2007; Finch et al., 2016a).

##### 3.4.1.2 Non-alkaloids

**FIGURE 3:**
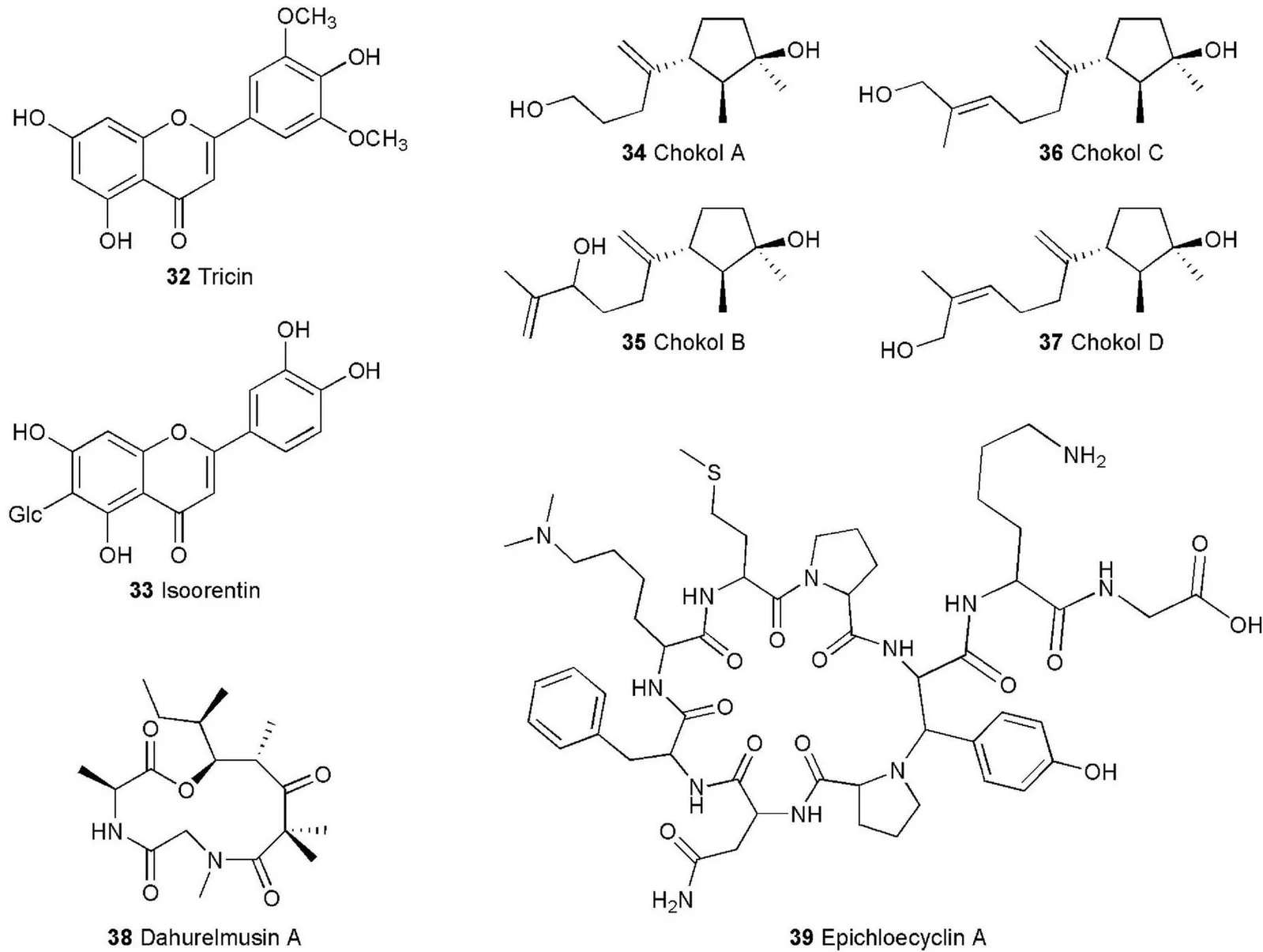
Chemical structures of known non-alkaloid secondary metabolites produced by *Epichloë* in host grasses.

Non-alkaloid secondary metabolites associated with *Epichloë* include flavonoids such as tricin (**33**) and isoorientin (**34**) (Ju et al., 1998), sesquiterpenoids (e.g., chokols (**35**–**38**), Hiroyuki et al., 1989), fatty acids (Koshino et al., 1987), a dipeptide-polyketide (Song et al., 2017) and cyclic octa/nona-peptides (Johnson et al., 2015; Figure 3). Some of these have been associated with anti-fungal activity (refer section 3.2.2) while the function of others remains unknown. The recently identified dipeptide-polyketide dahurelmusin A (**39**) has been indicated to provide resistance to aphids (Song et al., 2017). The epichloëcyclins [e.g., epichloëcyclin A **(40)**] are octa- and nonapeptides produced by non-ribosomally synthesised peptide (NRPS) that is cyclised via a ribosomally synthesised and post-translationally modified peptide system (RiPPs). Though produced by one of the most highly expressed genes *in planta*, the function of the epichloëcyclins is still unknown.

Additionally, fungal reactive oxygen species (i.e., highly reactive, unstable oxygen-containing molecules that are crucial for cellular communication and metabolism) production is critical in maintaining a mutualistic fungus–plant interaction (Tanaka et al., 2006).

### 3.5 Vulnerability of endophytes

The viability of endophyte in seed is primarily determined by the length of storage, temperature, and relative humidity which directly affects seed moisture levels (Rolston and Agee, 2007; Hume et al., 2013; Bradford et al., 2016). For valuable commercial seed lots, it is recommended that they are packed into moisture proof packaging and stored at a temperature of 5°C or less, relative humidity of 30% or less, and seed moisture of 8% or less (Hume et al., 2013). Transmission of endophyte to the developing seed is crucial for the life cycle of *Epichloë*, which at least for ryegrass, is heavily influenced by host genetics (Gagic et al., 2018).

## 4 The role of *Epichloë* in grazed temperate grasslands

The secondary metabolite chemistry produced *in planta* by *Epichloë* is primarily directed toward deterring herbivores from consuming the grass host. This ensures enduring plant persistence which is evident with Kentucky 31 tall fescue in United States (Bouton et al., 1993; Satognon, 2024) and ryegrasses in New Zealand and Australia with “wild type” standard endophyte (van Heeswijck and McDonald, 1992; Hume et al., 2020; Caradus, 2024; Hewitt et al., 2025). In both cases these endophytes produce ergovaline and/or lolitrem B that directly affect the health and wellbeing of the grazing ruminant in addition to deterring grazing (Ball et al., 1991; Hoveland, 1993; Bouton et al., 2001; Ball et al., 2019). Secondary metabolites from *Epichloë* also protect the host grass from insect pests (Breen, 1994; Caradus and Johnson, 2020) and pathogens (Card et al., 2021). It has been argued with reference to *Epichloë* that “plant secondary chemicals and endophytic alkaloids often fail to protect plants because: (1) specialist herbivores evolve to detoxify and use defensive chemicals for growth and survival; and (2) natural enemies of herbivores may be more negatively affected by alkaloids than are herbivores” (Faeth and Saari, 2012). However, over many decades in New Zealand where insect pressure can be severe, this has not been observed with both “wild type” (also called “common toxic” or “standard endophyte”) and commercial (novel selected strains) *Epichloë* strains used in perennial ryegrass. Improved drought tolerance due to *Epichloë* has been observed in tall fescue, although the biochemistry and/or mechanism causing this effect are largely unknown (Elmi and West, 1995; Elbersen and West, 1996; Nagabhyru et al., 2013; Xu et al., 2017).

**TABLE 3:** *Epichloë* alkaloids associated with providing protection of grass host against invertebrate pests.

Alkaloid compound	Invertebrate pest	Modes of protection	References
Compounds not known to cause animal health and welfare concerns			
Lolines	Aphids—*Rhopalosiphum padi* and *Schizaphis graminum*	Toxicity	Johnson et al., 1985; Latch et al., 1985; Siegel et al., 1990; Eichenseer et al., 1991; Wilkinson et al., 2000; Bultman et al., 2006; Bastías et al., 2017b
	Grass grub—*Costelytra giveni*	Feeding deterrent	Popay et al., 2003a; Patchett et al., 2008b; Patchett et al., 2011a
	Root aphid—*Aploneura lentisci*	Reduced root aphid numbers per plant	Jensen and Popay, 2007; Schmidt, 1993
	Red-headed cockchafer*—Adoryphorus coulonii*	Feeding deterrent	Bryant et al., 2010
	African black beetle—*Heteronychus arator*	Deterrent, antifeeding effect on larval and adult stages	Bryant et al., 2010; Barker et al., 2015a
	Argentine stem weevil—*Listronotus bonariensis*	Feeding deterrent and death of larvae	Patchett et al., 2008a; Jensen et al., 2009; Popay et al., 2009; Barker et al., 2015c
	Porina—*Wiseana cervinata*	Reduce feeding and weight gain	Popay and Lane, 2000
	Black field cricket—*Teleogryllus commodus*	Deterrent	Barker et al., 2015b
	Fall armyworm larvae—*Spodoptera frugiperda*	Deterrent—reduced worm survival and liveweight gains	Riedell et al., 1991
	Greenbugs—*Schizaphis graminum*	Toxicity	Riedell et al., 1991
	Japanese beetle larvae—*Popillia japonica*	Toxicity and deterrence	Patterson et al., 1991; Bush et al., 1993
	Rice leaf bug—*Trigonotylus caelestialium*	Toxicity and deterrence	Shiba and Sugawara, 2009
N-formyl loline	Cat flea larvae—*Ctenocephalides felis*	Contact toxicity	Bush et al., 1993
	Grass grub—*Costelytra giveni*	Toxicity	Popay and Lane, 2000; Popay and Tapper, 2007
	Large milkweed bug—*Oncopeltus fasciatus*	Feeding deterrent and toxic	Johnson et al., 1985; Yates et al., 1989
	American cockroach—*Periplaneta americana*	Contact toxicity	Bush et al., 1993
	Lesion nematode—*Pratylenchus scribneri*	Toxicity and deterrence	Kimmons et al., 1990; Bacetty et al., 2009
N-acetylnorloline	Argentine stem weevil—*Listronotus bonariensis*	Deterrent	Jensen et al., 2009
N-acetyl loline	European corn borer larvae—*Ostrinia nubilalis*	Toxic effects and reduced larval weight gain	Riedell et al., 1991
	Grass grub—*Costelytra giveni*	Deterrent	Popay and Tapper, 2007
Possibly loline (*Epichloë* strain was in cultivar Kentucky 31 in tall fescue)	Soil nematodes—*Pratylenchus scribneri* and *Tylenchorhynchus acutus*	Reduced infection of roots of tall fescue	West et al., 1988
Peramine	Argentine stem weevil—*Listronotus bonariensis* (larva), pasture mealy bug—*Balanococcus poae*	Feeding deterrent for both adults and larvae; reduced oviposition	Prestidge et al., 1982; Gaynor and Rowan, 1985; Rowan and Gaynor, 1986; Rowan et al., 1990; Pennell et al., 2005; Chacón-Fuentes et al., 2025
	Cutworm—*Graphania mutans*	Not a deterrent, but disrupted development	Dymock et al., 1989b
	Aphid—*Schizaphis graminum*	Feeding deterrent and toxic	Siegel et al., 1990
Compounds known to cause some animal health and welfare concerns			
Ergovaline	Argentine stem weevil—*Listronotus bonariensis*	Feeding deterrent	Dymock et al., 1989b; Popay et al., 1990
	African black beetle—*Heteronychus arator*	Ergopeptine alkaloids—ergotamine, ergovaline, ergocryptine	Ball and Prestidge, 1992, 1993; Popay and Ball, 1998
Ergovaline, ergotamine	Lesion nematode—*Pratylenchus scribneri*	Attractant and causes death	Bacetty et al., 2009
Ergovaline and/or ergine Ergonovine Ergocryptine, ergonovine	Black cutworm—*Agrostis ipsilon*	Deterrence and toxicity	Baldauf et al., 2011
	Aphids—*Rhopalosiphum padi*	Toxicity	Shymanovich et al., 2015
	Lesion nematode—*Pratylenchus scribneri*	Deterrent	(Bacetty et al., 2009)
	Large milkweed bug—*Oncopeltus fasciatus*	Toxic	Yates et al., 1989
Ergotamine, ergonovine, ergocryptine	Fall army worm—*Spodoptera frugiperda*	Reduced worm survival and liveweight gains	Clay and Cheplick, 1989
Chanoclavine	African Black beetle—*Heteronychus arator*	Mild deterrent	Hudson et al., 2021
Lysergic acid	African black beetle—*Heteronychus arator*	Deterrent	Hudson et al., 2021
Lolitrem B	Porina—*Wiseana* spp.	Reduce feeding and weight gain	Kaufononga, 2019
	Argentine stem weevil*—Listronotus bonariensis*	Toxic/feeding deterrent of larvae	Prestidge and Gallagher, 1985; Dymock et al., 1989a; Rowan, 1993; Popay et al., 2003b
	Mollusc—*Deroceras reticulatum*	Reduced feeding	Barker, 2008
Paxilline	Porina—*Wiseana cervinata*	Reduce feeding and weight gain	Babu, 2009; Kaufononga, 2019
	Argentine stem weevil—*Listronotus bonariensis*	Feeding deterrent	Rowan, 1993
Epoxyjanthitrems	Porina—*Wiseana* spp.	Reduced feeding and toxicity	Popay et al., 2012; Hennessy et al., 2016; Finch et al., 2020

### 4.1 Impact on grazing vertebrates

*Epichloë* produce alkaloids that can cause a variety of health and welfare concerns, and productivity losses, for both grazing ruminants and non-ruminants (Table 2). In United States, the cost of fescue toxicosis was estimated to be US$600 million per year over 20 years ago (Fribourg and Waller, 2004). In New Zealand, the estimated cost of ryegrass staggers was NZ$100 million per year 20 years ago (NZ Veterinary Association, 2007). Similarly, in Australia the estimated cost for perennial ryegrass toxicosis in sheep production systems was A$100 million per year (MLA, 2012). These negative effects of *Epichloë* endophytes on animals led to the proposition that endophyte-free grasses would be more beneficial for livestock production (Stuedemann and Hoveland, 1988; Bouton et al., 1993). However, it was soon realised that the endophyte was crucial to persistence of the grass host (Bacon and Siegel, 1988; Bacon, 1995; Easton, 1999; Easton et al., 2001) and resulted in programmes to deliver novel less mammalian toxic strains, which have been successfully commercialised for over 25 years (Bouton et al., 2002; Parish et al., 2003; Franzluebbers et al., 2009; Hopkins et al., 2010; Caldwell et al., 2013; Johnson and Caradus, 2019; Caradus and Johnson, 2020).

In addition to impacts of animal health and welfare disorders indirectly reducing levels of production (Table 2) some *Epichloë* chemistry (e.g., ergovaline) can also directly cause reduced levels of production through feeding deterrence (Stuedemann et al., 1989; Seman et al., 1990) resulting in reduce animal liveweight gains (Layton et al., 2004). However, the feeding deterrence due largely to ergovaline has been used as a benefit in turf grasses to deter birds from feeding on them when used in airports and playgrounds (Pennell et al., 2010; Pennell and Rolston, 2013; Pennell et al., 2016; Pennell et al., 2017b; Pennell et al., 2017a). Similar *Epichloë* chemistry, such as ergovaline, that maybe toxic to mammals and deter insect pests, aid in deterring insectivorous and predatory birds from those areas as well (Popay and Wyatt, 1995; Ball et al., 1997c; Popay and Ball, 1998).

The movement of *Epichloë* endophyte alkaloids to animal meat and milk has been examined and raise no food safety concerns. It has been shown that concentrations of epoxyjanthitrems (**20**–**24**) and lolitrem B (**19**) in sheep and dairy cow fat can increase when concentrations in pasture increase but then decrease when animals were removed from pastures containing these compounds (Finch et al., 2012; Finch et al., 2013; Duringer et al., 2021). There is no convincing evidence that ergot alkaloids accumulate in edible tissues of animals fed ergot alkaloids (Anon, 2005; Schumann et al., 2009; Zbib et al., 2015).

### 4.2 Impact on growth and persistence of pasture grasses

*Epichloë* endophytes particularly in New Zealand, Australia and United States have been shown to improve both the persistence and yield of important temperate grasses (Hume and Sewell, 2014; Thom et al., 2014; Caradus, 2024). Interestingly, with many of these originating in Europe (particularly for perennial ryegrass and tall fescue) the selective advantage of *Epichloë* endophyte is not immediately obvious but has been suggested as an option for sustainable agriculture (Kauppinen et al., 2016; von Cräutlein et al., 2021). However, where *Epichloë* is important for yield and persistence it is due primarily to providing resistance and/or tolerance to biotic and abiotic stress (Lee et al., 2021). It has been stated that “over 40 insect pests and diseases have been documented as responding to endophyte infection” (Stewart et al., 2022).

#### 4.2.1 Insect pest resistance

In some temperate grassland environments (e.g., New Zealand; Caradus, 2024; Card et al., 2024) *Epichloë* is crucial for the persistence of grasses against a wide range of invertebrate pests. The known impact of various alkaloids produced by *Epichloë* on insect pests are primarily either due to toxicity or deterring feeding and are summarised in Table 3. There are however several *Epichloë* strains that effectively control the impact of insect pests but for which the chemistry responsible is still unknown. For example, the control of root aphid (*Aploneura lentisci*) by strains AR5 and AR37 (Popay et al., 2021); reduced numbers of African black beetle and reduced feeding by Argentine stem weevil (*Listronotus bonariensis*) by strain AR37 (Popay et al., 2008); reduced numbers of *Philobota* spp.—pasture tunnel moths and *Pseudococcidae*—mealybugs by strain AR37 (Moate et al., 2012); and reduced fall army (*Spodoptera frugiperda*) larval growth and feeding with *Epichloë* strains in meadow fescue and selected strains in tall fescue (Lee et al., 2025); and strains AR47 and AR48 have been shown to have a deterrent and/or toxic effects on wheat sheath miner (*Cerodontha australis*) (Jensen et al., 2022); an undocumented *Epichloë* strain that provided resistance to sod webworm (*Crambus* spp.) (Funk et al., 1983); and naturally *Epichloë*-infected native Iranian tall fescue providing biological control of Elm-grass root aphid (*Tetraneura ulmi*) and barley aphid (*Sipha maydis*) (Mohammadi et al., 2024).

While the focus for the impact of *Epichloë* on insect pests has been through alkaloid production there is evidence that some insect resistance may come from genes transferred horizontally into *Epichloë* from bacterial donors (Ambrose et al., 2014).

#### 4.2.2 Pathogen resistance

Early *Epichloë* studies referred to production of antifungal substance(s) in *in vitro* assays with fungal pathogens such as *Rhizoctonia cerealis* (White and Cole, 1986). Other reports of *Epichloë* suppressing other fungal diseases of their host grass include dollar spot disease (*Sclerotinia homoeocarpa*) in fine fescues (*Festuca rubra* subsp. *rubra*) (Clarke et al., 2006), *Rhizoctonia zeae* in tall fescue (Gwinn and Gavin, 1992), and barley yellow dwarf virus in meadow fescue due to reduced aphid numbers (Lehtonen et al., 2006). *Epichloë* can protect the grass host plant against pathogens both directly through the production of fungicidal compounds, competition for a common resource, or the induction of plant defenses, and indirectly through endophyte-generated changes in the abiotic or the biotic environment (Pérez et al., 2020). A meta-analysis of 22 publications, covering a total of 18 host grass species, 11 *Epichloë* endophyte species, and 22 fungal pathogen species, demonstrated a negative overall effect of *Epichloë* on infection by pathogens (Pérez et al., 2020). Interestingly, but perhaps not surprisingly, in that analysis the protective impact of the *Epichloë* was greater in laboratory and glasshouse trials than in field trials. An extensive summary of both *in vitro* and *in planta* bioactivity exhibited by species of *Epichloë* toward fungal saprophytes or phytopathogens is provide by Card et al. (2021).

Using *in vitro* assays, it has been confirmed that the chemistry responsible for the antifungal activity observed by *Epichloë* endophytes is not due to the known *Epichloë*-derived antimammalian and insecticidal alkaloids and their intermediates [peramine (**26**), *N*-formylloline (**29**), *N*-acetylloline (**27**), lolitrem B (**20**), epoxyjanthitrem I (**22**), paxilline (**13**), terpendole E (**14**), terpendole C (**16**), ergovaline (**6**)] (Siegel and Latch, 1991; Christensen, 1996; Fernando et al., 2021). Chemistry associated with providing antifungal activity is described in Table 4. Some *Epichloë* species do not necessarily produce some of these compound, e.g., *E. festucae* and *E. bromicola* did not produce chokol K (**38**) (Steinebrunner et al., 2008a). Additionally, the inhibition of fungal pathogens can be endophyte strain specific within a species of *Epichloë* (Niones and Takemoto, 2014; Fernando et al., 2021; Li et al., 2023).

**TABLE 4:** Chemistry associated with antifungal activity provide by *Epichloë.*

Chemical	*Epichloë* species	Pathogens active against	Effect and testing procedure	References
Sesquiterpinoid—Chokol K	*E. sylvatica* *E. clarkii*	*Stagonospora nodorum* *Mycosphaerella graminicola*	Reduced the spore germination of all tested fungi—tested in culture	Steinebrunner et al., 2008a
Sesquiterpinoids—Chokols A, B, C	*E. typhina*		Known to be fungitoxic	Yoshihara et al., 1985
Sesquiterpenoids—Chokols A, B, C, D, E, F and G	*E. typhina*		Known to be fungitoxic	Hiroyuki et al., 1989
Protein—designated Efe-AfpA	*E. festucae*	*Sclerotinia homoeocarpa*	*In vitro* petri dish assay with extracted compound	Tian et al., 2017
Indole derivatives—indole-3-acetic acid and indole-3-ethanol	*E. festucae*	*Cryphonectria parasitica*	Isolated from organic extracts from liquid fermentation cultures	Yue et al., 2000
Fatty acids—C-18 hydroxy unsaturated fatty acids	*E. typhina*		Noted as fungitoxic	Koshino et al., 1987
Peptide—ε-poly-L-lysine	*E. festucae*	*Drechslera erythrospila, Botrytis cinerea, Phytophthora infestans*	Reduced the spore germination of all tested fungi and hyphal growth *in vitro*	Purev et al., 2020
Gamahonolides—Gamahonolides A and B, gamahorin, and 5-hydroxyl-4-phenyl-2(5H)-furanone	*E. typhina*		Noted as fungitoxic	Koshino et al., 1992
Phenolics—1,3-O-di-trans-p-coumaroylglycerol, 1,2-O-di-trans-p-coumaroylglycerol, chokorin	*E. typhina*		Noted as fungitoxic	Koshino et al., 1988
Cyclic peptide—epichlicin	*E. typhina*	*Cladosporium phlei*	Reduced the spore germination	Seto et al., 2007
Cyclic peptide—Cyclosporin T	*E. bromicola*	*Alternaria alternata, Fusarium avenaceum, Bipolaris sorokiniana, Curvularia lunata*		Song Q. Y. et al., 2015
Protease and endoglucanase activity possibly related to *vibA* gene which was associated with anti-fungal activity	*E. festucae*	*Drechslera erythrospila, D. siccans, D. dictyoides, Colletotrichum graminicola, Bipolaris sorokiniana*	Inhibited hyphal growth conidial germination	Niones and Takemoto, 2014; Niones and Takemoto, 2015

The antagonism of *Epichloë* toward multiple species of saprophytic and pathogenic microbes associated with some temperate grasses has been reviewed by Card et al. (2021). In addition to the secondary metabolites list in Table 4, *Epichloë*-mediated induction of the plant’s own defenses has been proposed as another means of providing resistance to phytopathogens (Xia et al., 2018; Pérez et al., 2020).

#### 4.2.3 Abiotic stress tolerance

In addition to “protecting” the host plant from numerous biotic challenges, *Epichloë* also provide some benefits that reduce abiotic stress impacts (Malinowski et al., 2005; Malinowski and Belesky, 2019; Lee et al., 2021). The most notable is improved tolerance to drought or low soil moisture environments (Arachevaleta et al., 1989; Eerens et al., 1998; West et al., 1993; Malinowski and Belesky, 2000; Kannadan and Rudgers, 2008; Li et al., 2008; Kane, 2011; Decunta et al., 2021). However, there are some studies reporting that endophyte status does not affect plant responses to drought (White et al., 1992; West, 1994; Cheplick et al., 2000; Rudgers and Swafford, 2009; He et al., 2017), although these were glasshouse pot trials. Where *Epichloë* has shown improved drought tolerance, the effect in fescue has been a reduction in stomatal conductance and improved osmotic adjustment, particularly in the meristematic and growing zone of the plant resulting in improved recovery from drought (Bacon, 1993; Elmi and West, 1995; Elbersen and West, 1996). The imposition of water deficit has also been shown to increase free glucose, fructose, trehalose, sugar alcohols, proline and glutamic acid in shoots and roots, along with the fungal metabolites, mannitol, loline alkaloids (Nagabhyru et al., 2013) and in ryegrass ergovaline and lolitrem B (Hahn et al., 2008). For perennial ryegrass, some *Epichloë* strains such as AR37 have been shown to increase root system growth (Hume, 2018) which may provide the shoot with a better supply of nutrients and water in periods of stress. Changes in antioxidant enzyme activity due to *Epichloë* presence has also been linked to improved recovery from drought (Xu et al., 2017). The positive effects of *Epichloë* on ryegrass persistence during drought become even more important if coinciding with insect attack, confirming their critical importance in many intensively managed pastoral systems (Hewitt et al., 2021).

Improvements have also been shown for tolerance to salinity (Li et al., 2008; Sabzalian and Mirlohi, 2010; Yin et al., 2014; Wang et al., 2020), tolerance to high aluminum (Malinowski and Belesky, 1999; Zaurov et al., 2001) or high nickel (Mirzahosseini et al., 2014) soils, and improved nutrient uptake in low nutrient (e.g., phosphorus) soils (Malinowski and Belesky, 2000) due to *Epichloë* infection in grasses. However, the mechanisms for these improvements are not necessarily well understood. Some may be due to improved resilience with the host plants protected from some insect pests and pathogens by the *Epichloë* endophyte (Lee et al., 2021).

## 5 The development of commercially viable *Epichloë* strains

Following the discovery of *Epichloë* endophytes in temperate grasses, such as ryegrass and fescue, and their links to both animal health and welfare issues, as well as enhanced host-plant persistence, efforts were initiated to identify *Epichloë* strains that retained agronomic benefits while mitigating negative impacts on livestock. This search focused on endophyte strains capable of sustaining host persistence without compromising animal health and welfare (Easton, 1999; Easton et al., 2001; Gunter and Beck, 2004; Phillips and Aiken, 2009; Johnson et al., 2013; Johnson and Caradus, 2019; Caradus and Johnson, 2020; Caradus et al., 2021b; Eady, 2021; Caradus, 2024; Hewitt et al., 2025). This led to the identification for what has been termed “novel” or “selected” endophytes.

### 5.1 Ryegrasses

In New Zealand, and to a lesser extent in Australia, the focus on identifying novel *Epichloë* for ryegrasses has been to reduce the potential for ryegrass staggers and heat stress, and as a result cause less dags/flystrike in sheep and remove the hand brake on milk production and live weight gain (Hume et al., 2025). To ensure success, plant breeders needed to ensure that the genetic compatibility between the selected *Epichloë* strain and host plant was effective, so that transmission through seed and establishment in resulting seedlings was effective (Christensen et al., 1997; Gagic et al., 2018; Zhang et al., 2022). The first strain successfully commercially released was AR1 in 2001—aimed at providing resistance to Argentine stem weevil with very low levels of ryegrass staggers and no heat stress (Fletcher, 1999; Popay et al., 1999). Ryegrass cultivars with AR1 endophyte strain were rapidly adopted by farmers (Caradus et al., 2013). Evaluation under dairying indicated that AR1-infected ryegrass while producing similar pasture yields as standard endophyte-infected ryegrass, it also provided small (6.7%) improvements in milk yield with no incidence of ryegrass staggers in grazing animals (Bluett et al., 2005). This has been followed by numerous other novel strains with different profiles in terms of insect pest resistance while keeping animal health and welfare concerns to a minimum (Caradus et al., 2021b; Table 5). The most notable outcome of this work has been the development of the *Epichloë* strain AR37. This endophyte has been shown to substantially increase perennial ryegrass production, with national gains of approximately 12% and increases of up to 37% in northern New Zealand compared with standard *Epichloë* strains over a 4-year period (Hume et al., 2007). More recent evaluations report production increases of up to 33% and 55% relative to AR1 and endophyte-free cultivars, respectively (Ferguson et al., 2025). In addition, AR37 confers resistance against five of the six major insect pests affecting ryegrass (Young et al., 2013), extending pasture persistence such that average renewal intervals may increase from 5 to 8 years (Thom et al., 2013; PGWS, 2026). Importantly, it also reduces the severity of ryegrass staggers compared with standard endophyte strains (Fletcher et al., 2017; Caradus and Johnson, 2020; Caradus et al., 2021b; Eady, 2021). Consequently, AR37, 1924 has remained the dominant *Epichloë* endophyte incorporated into proprietary ryegrass cultivars in New Zealand through to the mid-2020s (Caradus et al., 2025). The success of *Epichloë* endophytes has resulted in > 90% of all New Zealand bred or marketed proprietary forage tall fescue, perennial and hybrid ryegrass cultivars containing a novel *Epichloë* endophyte (Milne, 2007; Chynoweth et al., 2015).

**TABLE 5:** Insect pests that commercialized strains of *Epichloë* for use in pasture grasses have been documented to protect their host plant.

Insect pest	*Epichloë* Brands (or strains)		References
	High level of protection	Moderate to low level of protection	
Epichloë festucae var. lolii in ryegrasses			
Argentine stem weevil—*Listronotus bonariensis*	Standard, AR1, NEA2, NEA4, AR37^1^, NEA12^1^, AR128, Endo5, RGT18, CM142	(Edge)	Popay and Hume, 2011; Thom et al., 2014; Popay et al., 2017; Caradus et al., 2025; PBRA, 2026a
Pasture mealy bug—*Balanococcus poae*	Standard, AR1, NEA2, NEA4^2^, AR37^1^, Endo5, NEA, (Happe^4^)	(Edge)	Pennell et al., 2005; Popay and Hume, 2011; Popay et al., 2021; PBRA, 2026a; Ferguson, personal communication April 2026
African black beetle—*Heteronychus arator*	Standard, NEA2, NEA4^2^, AR37^1^, NEA12^1^, AR128, Endo5, NEA, RGT18, CM142, (Happe^4^)	AR1	Popay and Hume, 2011; Thom et al., 2014; Caradus et al., 2025; PBRA, 2026a; Ferguson, personal communication April 2026
Root aphid—*Aploneura lentisci*	AR37^1^, (AR6^3^) NEA12^1^, AR128, RGT18, CM142, (Happe^4^)	Standard, NEA2, NEA4	Popay and Gerard, 2007; Pennell et al., 2005; Popay and Hume, 2011; Thom et al., 2014; Popay and Cox, 2016; Popay et al., 2021; Hewitt et al., 2024a; Caradus et al., 2025; PBRA, 2026a; Ferguson, personal communication April 2026
Porina—*Wiseana cervinata*	AR37^1^, NEA12^1^, AR128, RGT18, CM142, (Happe^4^)	Standard	Jensen and Popay, 2004; Popay and Hume, 2011; Caradus et al., 2025; PBRA, 2026a; Ferguson, personal communication April, 2026
Grass grub—*Costelytra giveni*		AR37, (Happe^4^)	Popay and Thom, 2009; Hewitt et al., 2024b; PBRA, 2026a; Ferguson, personal communication April, 2026
Epichloë coenophialum in fescues			
Argentine stem weevil—*Listronotus bonariensis*	AR542 (MaxQ)		Popay et al., 2005
African black beetle—*Heteronychus arator*	AR542 (MaxQ), AR584—MaxQII,		Popay et al., 2005; PBRA, 2026a
Root aphid—*Aploneura lentisci*	AR542 (MaxQ), AR584—MaxQII		Popay and Jensen, 2005; PBRA, 2026a
Grass grub—*Costelytra giveni*	AR1017^5^ MaxR	AR542 (MaxQ), AR584—MaxQII,	Popay and Tapper, 2007; Hewitt et al., 2024b; PBRA, 2026a
Black field cricket—*Teleogryllus commodus*	AR584—MaxQII		PBRA, 2026a
Epichloë uncinata in festulolium			
Argentine stem weevil—*Listronotus bonariensis*	U2		Patchett et al., 2008a; Barker et al., 2015c; Popay et al., 2017; PBRA, 2026a
Pasture mealy bug—*Balanococcus poae*	U2		PBRA, 2026a
African black beetle—*Heteronychus arator*	U2		Barker et al., 2015a; PBRA, 2026a
Root aphid—*Aploneura lentisci*	U2		PBRA, 2026a
Porina—*Wiseana cervinata*	U2		PBRA, 2026a
Grass grub—*Costelytra giveni*		U2	Patchett et al., 2011a; PBRA, 2026a
Black field cricket—*Teleogryllus commodus*	U2		Barker et al., 2015b; PBRA, 2026a

Information from Caradus and Johnson (2020), Caradus et al. (2021a,b), Caradus (2024) and PBRA (2026a) has been integrated into this table. * Brackets indicate the strain is no longer commercially available.

^1^*Epichloë* sp. LpTG-3.

^2^*Epichloë* sp. LpTG-2.

^3^ *Epichloë hybrida.*

^4^ *Epichloë siegelii.*

*^5^E. uncinata.*

### 5.2 Fescues

The priority for a novel *Epichloë* endophyte for fescues was to eliminate the occurrence of fescue toxicosis. This was effectively achieved through the release of tall fescue endophyte strain AR542 (branded MaxQ in USA and MaxP in New Zealand/ Australia) in 2000 and then AR584 (branded MaxQII in USA and MaxP in New Zealand/ Australia) (Bouton, 2009; Phillips and Aiken, 2009; Young et al., 2014; Johnson and Caradus, 2019; Card et al., 2024). To date there have been relatively few other tall fescue novel *Epichloë* strains successfully commercialised, with only ArkShield, Protek, and E34, joining MaxQII on the market (Smith and Phillips, 2016; Caradus and Johnson, 2020; Allen et al., 2021). In New Zealand, a novel strain for meadow fescue (strain AR1017, branded MaxR) has also been commercialised (Stewart et al., 2025).

### 5.3 Other pasture grasses

While grasses other than ryegrass or fescue do form mutualistic associations with *Epichloë*, none have been commercialised successfully.

### 5.4 Turf grasses

The *Epichloë*–grass mutualism, and resulting impacts on insect pests and mammals, makes it an ideal technology for use in areas where grazing animals are absent and grass cover is required, such as in parks, sports playing surfaces and airports (Funk et al., 1993; Meyer et al., 2013; Stewart et al., 2022; Reid et al., 2025). It has been shown that some of the mammalian toxic alkaloids expressed by *Epichloë* act as deterrents to not just insects, but also birds, mice and rabbits (Pennell et al., 2010; Finch et al., 2016b; Pennell et al., 2016). As a result, an *Epichloë* endophyte product has been released for specific use at airports and in parks and playing areas and branded Avanex™, using endophyte strains AR95 for ryegrasses and AR601 for tall fescue (Pennell and Rolston, 2013; Pennell et al., 2016; Pennell et al., 2017b; Pennell et al., 2017a).

### 5.5 Commercialisation code of practice

Proprietary forage seed companies in New Zealand (PBRA, 2026b) and USA (Alliance for Grassland Renewal, 2026) have agreed that for a tall fescue or ryegrass seed line to be labeled as “endophytic” then at least 70% of seed must contain viable *Epichloë* endophyte. For New Zealand the agreed wording includes “its members, adopt the position that for grade endophytic ryegrass and fescue seed to be sold, that there is a current seed test to confirm that at least 70% of seed will be infected with live endophyte at the time of sale to the retailer.” In Australia, a unified seed company position is still to be confirmed but individually some companies agree that for an endophyte to be effective on farm, at least 70% of the seed needs to contain a live endophyte (DLF Seeds Australia, 2026).

## 6 Concluding comments

**FIGURE 4:**
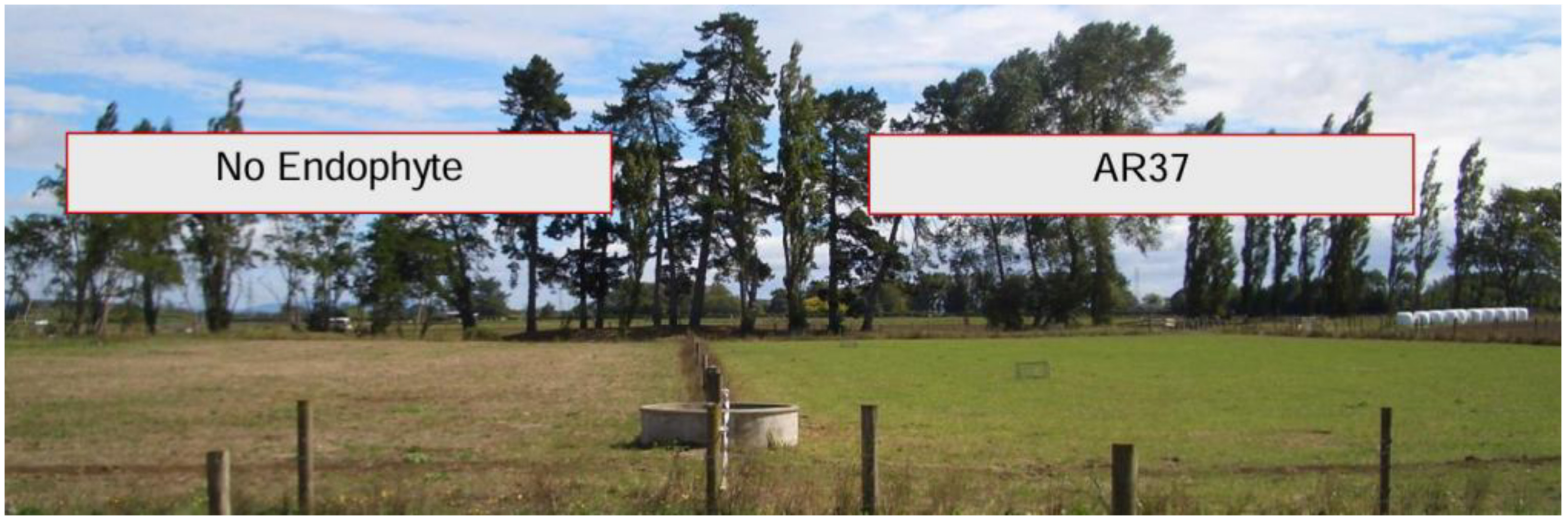
Effect of *Epichloë* endophyte strain AR37 (right) compared with nil endophyte (left) on perennial ryegrass persistence and growth in New Zealand.

Asexual *Epichloë* endophytes in New World pastures (e.g., New Zealand, Australia, and USA) provide considerable benefit to the persistence of temperate grass pastures (Bouton, 2009; Thom et al., 2012; Figure 4). They are highly efficacious, making them one of the most successful biocontrol products. However, balancing that benefit while ensuring reduced or nil animal health and welfare concerns has been a major driver in delivering *Epichloë* strains of commercial value. Clearly in these pastures the absence of *Epichloë* endophyte results in poor persistence and the need for ongoing pasture renewal, which is both expensive and has considerable environmental downside through soil disturbance. Over the last 25 years several strains of *Epichloë* have been successful commercialised in New Zealand, Australia and USA. Being maternally inherited they are part of the seed and move into the seedling grass plant upon germination. Pastoral farmers can use *Epichloë* endophytic grasses without it being disruptive to their traditional on-farm management systems. To ensure viable endophyte is maintained in seed during storage, seed is recommended to be kept at 5°C or less and relative humidity of 30% or less. While *Epichloë* has to date been used only in temperate grasses it may have application in some cereals which while not having *Epichloë* are genetically related to some wild grasses that do harbor *Epichloë* (Card et al., 2014; Simpson et al., 2014; Wood et al., 2024).

The *Epichloë*–grass association represents a stable, co-evolved symbiosis in which microbial function is embedded within plant life history, supporting consistent performance across seasons and variable stress regimes. Its success challenges conventional distinctions between genetics, management, and environment by demonstrating that adaptive capacity can be distributed across plant–microbe assemblages rather than residing solely within the plant genome. In New Zealand, the implications extend beyond individual pastures; by underpinning the persistence and productivity of temperate grasslands, *Epichloë* endophytes have helped stabilise the pastoral foundation of the national agricultural economy. Their widespread adoption illustrates how biologically embedded microbial symbioses can enhance resilience, reduce system vulnerability, and support long-term agricultural viability at national scale. There is little doubt that *Epichloë* typifies the stunning role that microbial symbioses can play in improving resilience and yield in plants of economic importance.

## References

[B1] Alabdouli K. O. Blythe L. L. Duringer J. M. Elkhouly A. Kassab A. Askar A. M. et al. (2014). Physiological effects of endophyte-infected perennial ryegrass straw on female camels in the Middle East. *Emirates J. Food Agric.* 26 82–92. 10.9755/ejfa.v26i1.16473

[B2] Allen I. Robertson S. M. Broster J. C. Friend M. A. (2021). Evaluation of tall fescue cultivars containing endophytes on pasture productivity and lamb performance. *Small Ruminant Res.* 202:106463. 10.1016/j.smallrumres.2021.106463

[B3] Alliance for Grassland Renewal (2026). *Quality Control.* Available online at: https://grasslandrenewal.org/quality-control/ (accessed March 22, 2026).

[B4] Ambrose K. V. Koppenhöfer A. M. Belanger F. C. (2014). Horizontal gene transfer of a bacterial insect toxin gene into the *Epichloë* fungal symbionts of grasses. *Sci. Rep.* 4:5562. 10.1038/srep05562 24990771 PMC4080199

[B5] Anon (2005). Opinion of the scientific panel on contaminants in food chain on a request from the commission related to ergot as undesirable substance in animal feed question N° EFSA-Q-2003-38. *EFSA J.* 225 1–27. 10.2903/j.efsa.2005.225

[B6] Arachevaleta M. Bacon C. W. Hoveland C. S. Radcliffe D. E. (1989). Effect of the tall fescue endophyte on plant response to environmental stress. *Agron. J.* 81 83–90. 10.2134/agronj1989.00021962008100010015x

[B7] Babu J. (2009). *Bioactive Chemicals of Importance in Endophyte-Infected Grasses.* Thesis, Doctor of Philosophy (PhD), Hamilton: The University of Waikato. Available online at: https://researchcommons.waikato.ac.nz/handle/10289/2608

[B8] Babu J. V. Popay A. J. Miles C. O. Wilkins A. L. di Menna M. E. Finch S. C. (2018). Identification and structure elucidation of janthitrems A and D from *Penicillium janthinellum* and determination of the tremorgenic and anti-insect activity of janthitrems A and B. *J. Agric. Food Chem.* 66 13116–13125. 10.1021/acs.jafc.8b04964 30482018

[B9] Bacetty A. A. Snook M. E. Glenn A. E. Noe J. P. Nagabhyru P. Bacon C. W. (2009). Chemotaxis disruption in *Pratylenchus scribneri* by tall fescue root extracts and alkaloids. *J. Chem. Ecol.* 35 844–850. 10.1007/s10886-009-9657-x 19575265

[B10] Bacon C. W. (1993). Abiotic stress tolerances (moisture, nutrients) and photosynthesis in endophyte-infected tall fescue. *Agric. Ecosyst. Environ.* 44 123–141. 10.1016/0167-8809(93)90042-N

[B11] Bacon C. W. (1995). Toxic endophyte-infected tall fescue and range grasses: Historic perspectives. *J. Animal Sci.* 73 861–870. 10.2527/1995.733861x 7608021

[B12] Bacon C. W. Siegel M. R. (1988). Endophyte parasitism of tall fescue. *J. Prod. Agric.* 1 45–55. 10.2134/jpa1988.0045

[B13] Bacon C. W. Lyons P. C. Porter J. K. Robbins J. D. (1986). Ergot toxicity from endophyte-infected grasses: A review. *Agron. J.* 78 106–116. 10.2134/agronj1986.00021962007800010023x

[B14] Bacon C. W. Porter J. K. Robbins J. D. Luttrell E. S. (1977). *Epichloe typhina* from toxic tall fescue grasses. *Appl. Environ. Microb.* 34 576–581. 10.1128/aem.34.5.576-581.1977 931377 PMC242703

[B15] Baldauf M. W. Mace W. J. Richmond D. S. (2011). Endophyte mediated resistance to black cutworm as a function of plant cultivar and endophyte strain in tall fescue. *Environ. Entomol.* 40 639–647. 10.1603/EN09227 22251642

[B16] Ball D. M. Lacefield G. D. Hoveland C. S. (1991). *The Tall Fescue Endophyte.* Available online at: https://uknowledge.uky.edu/anr_reports/33 (accessed March 18, 2026).

[B17] Ball D. M. Lacefield G. D. Hoveland C. S. (2019). *The Wonder Grass: The Story of Tall Fescue in the United States.* Salem, OR: Oregon Tall Fescue Commission.

[B18] Ball O. J.-P. Prestidge R. A. (1992). The effect of the endophytic fungus *Acremonium lolii* on adult black beetle (*Heteronychus arator*) feeding. *Proc. N.Z. Plant Prot. Conf.* 45 201–204. 10.30843/nzpp.1992.45.11260

[B19] Ball O. J.-P. Prestidge R. A. (1993). “The use of the Endophytic Fungus *Acremonium lolii* as a Biological Control Agent of Black Beetle, *Heteronychus arator* (Coleoptera: Scarabaeidae),” in *Proceedings of the 6th Australasian Conference on Grassland Invertebrate Ecology*, ed. Prestidge R. A. (Hamilton).

[B20] Ball O. J.-P. Tapper B. A. (1999). “The Production of Loline Alkaloids in Artificial and Natural Grass/Endophyte Associations,” in *Proceedings of the 52nd New Zealand Plant Protection Conference, Auckland Airport Royal-Centra*, (Auckland).

[B21] Ball O. J.-P. Barker G. M. Prestidge R. A. Lauren D. R. (1997a). Distribution and accumulation of the alkaloid peramine in *Neotyphodium lolii*-infected perennial ryegrass. *J. Chem. Ecol.* 23 1419–1434. 10.1023/B:JOEC.0000006473.26175.19

[B22] Ball O. J.-P. Barker G. M. Prestidge R. A. Sprosen J. M. (1997b). Distribution and accumulation of the mycotoxin lolitrem B in *Neotyphodium lolii*-infected perennial ryegrass. *J. Chem. Ecol.* 23 1435–1449. 10.1023/B:JOEC.0000006474.44100.17

[B23] Ball O. J.-P. Miles C. O. Prestidge R. A. (1997c). Ergopeptine alkaloids and *Neotyphodium lolii* mediated resistance in perennial ryegrass against adult *Heteronychus arator* (Coleoptera: Scarabaeidae). *J. Econ. Entomol.* 90 1382–1391. 10.1093/jee/90.5.1382

[B24] Barker G. M. (2008). Mollusc herbivory influenced by endophytic claviciptaceous fungal infections in grasses. *Ann. Appl. Biol.* 153 381–393. 10.1111/j.1744-7348.2008.00267.x

[B25] Barker G. M. Patchett B. J. Cameron N. E. (2015a). *Epichloë uncinata* infection and loline content afford *Festulolium grasses* protection from black beetle (*Heteronychus arator*). *N.Z. J. Agric. Res.* 58 35–56. 10.1080/00288233.2014.978480

[B26] Barker G. M. Patchett B. J. Cameron N. E. (2015b). *Epichloë uncinata* infection and loline content protect *Festulolium* grasses from crickets (Orthoptera: Gryllidae). *J. Econ. Entomol.* 108 789–797. 10.1093/jee/tou058 26470191

[B27] Barker G. M Patchett B. J. Gillanders T. Brown G. Montel S. Cameron N. (2015c). Feeding and oviposition by Argentine stem weevil on *Epichloë uncinata*-infected loline-containing *Festulolium*. *N.Z. Plant Prot.* 68 212–217. 10.1093/jee/tou058 26470191

[B28] Bastías D. A. Applegate E. R. Gundel P. E. Johnson L. J. Mace W. J. Moon C. D. et al. (2024). “After Air, Light, and Water, the Next Most Important Thing is Grass: An Introduction to the *Epichloë*–grass Symbiosis,” in *Fungal Associations*, eds Hsueh Y. P. Blackwell M. (Cham: Springer International Publishing).

[B29] Bastías D. A. Martínez-Ghersa M. A. Ballaré C. L. Gundel P. E. (2017a). *Epichloë* fungal endophytes and plant defenses: Not just alkaloids. *Trends Plant Sci.* 22 939–948. 10.1016/j.tplants.2017.08.005 28923242

[B30] Bastías D. A. Ueno A. C. Machado Assefh C. R. Alvarez A. E. Young C. A. Gundel P. E. (2017b). Metabolism or behavior: Explaining the performance of aphids on alkaloid-producing fungal endophytes in annual ryegrass (*Lolium multiflorum*). *Oecologia* 185 245–256. 10.1007/s00442-017-3940-2 28879573

[B31] Becker Y. Eaton C. J. Brasell E. May K. J. Becker M. Hassing B. et al. (2015). The fungal cell-wall integrity MAPK cascade is crucial for hyphal network formation and maintenance of restrictive growth of *Epichloë festucae* in symbiosis with *Lolium perenne*. *Mol. Plant-Microbe Inter.* 28 69–85. 10.1094/MPMI-06-14-0183-R 25303335

[B32] Berry D. Lee K. Winter D. Mace W. Becker Y. Nagabhyru P. et al. (2022). Cross-species transcriptomics identifies core regulatory changes differentiating the asymptomatic asexual and virulent sexual life cycles of grass-symbiotic *Epichloë* fungi. *G3* 12:jkac043. 10.1093/g3journal/jkac043 35191483 PMC8982410

[B33] Berry D. Mace W. Grage K. Wesche F. Gore S. Schardl C. L. et al. (2019). Efficient nonenzymatic cyclization and domain shuffling drive pyrrolopyrazine diversity from truncated variants of a fungal NRPS. *Proc. Nat. Acad. Sci. U. S. A.* 116 25614–25623. 10.1073/pnas.1913080116 31801877 PMC6926027

[B34] Bhambhani S. Kondhare K. R. Giri A. P. (2021). Diversity in chemical structures and biological properties of plant alkaloids. *Molecules* 26:3374. 10.3390/molecules26113374 34204857 PMC8199754

[B35] Bharadwaj R. Jagadeesan H. Kumar S. R. Ramalingam S. (2020). Molecular mechanisms in grass-*Epichloë* interactions: Towards endophyte driven farming to improve plant fitness and immunity. *World J. Microbiol. Biotechnol.* 36:92. 10.1007/s11274-020-02868-5 32562008

[B36] Bluett S. J. Thom E. R. Clark D. A. Waugh C. D. (2005). Effects of a novel ryegrass endophyte on pasture production, dairy cow milk production and calf liveweight gain. *Aust. J. Exptal Agric.* 45 11–19. 10.1071/ea03263

[B37] Bluett S. Hume D. Tapper B. Stephens S. (2004). Incidence of ryegrass staggers in white rhinoceros (*Ceratotherium simum*) at Auckland Zoo. *N. Z. Vet. J.* 52:48. 10.1080/00480169.2004.36392 15768084

[B38] Bourke C. A. Hunt E. Watson R. (2009). Fescue-associated oedema of horses grazing on endophyte-inoculated tall fescue grass (*Festuca arundinacea*) pastures. *Aust. Vet. J.* 87 492–498. 10.1111/j.1751-0813.2009.00519.x 19930166

[B39] Bouton J. (2009). “Deployment of novel endophytes in the tall fescue commercial seed trade,” in *Tall Fescue for the Twenty-First Century*, eds Fribourg H. Hannaway D. West C. (Madison, WI: American Society of Agronomy, Crop Science Society of America, Soil Science Society of America), 367–375. 10.2134/agronmonogr53.c20

[B40] Bouton J. H. Gates R. N. Hoveland C. S. (2001). Selection for persistence in endophyte-free Kentucky 31 tall fescue. *Crop Sci.* 41 1026–1028. 10.2135/cropsci2001.4141026x

[B41] Bouton J. H. Gates R. N. Belesky D. P. Owsley M. (1993). Yield and persistence of tall fescue in the Southeastern Coastal Plain after removal of its endophyte. *Agron. J.* 85 52–55. 10.2134/agronj1993.00021962008500010011x

[B42] Bouton J. H. Latch G. C. M. Hill N. S. Hoveland C. S. McCann M. A. Watson R. H. et al. (2002). Reinfection of tall fescue cultivars with non-ergot alkaloid–producing endophytes. *Agron. J.* 94 567–574. 10.2134/agronj2002.5670

[B43] Bradford K. J. Dahal P. Bello P. (2016). Using relative humidity indicator paper to measure seed and commodity moisture contents. *Agricult. Environ. Lett.* 1:160018. 10.2134/ael2016.04.0018

[B44] Breen J. P. (1994). *Acremonium* endophyte interactions with enhanced plant resistance to insects. *Ann. Rev. Entomol.* 39 401–423. 10.1146/annurev.en.39.010194.002153

[B45] Bryant R. H. Cameron N. E. Edwards G. R. (2010). Response of black beetle and red-headed pasture cockchafer larvae to loline alkaloids in meadow fescue roots. *Proc. N. Z. Plant Protect.* 63 219–223. 10.30843/nzpp.2010.63.6571

[B46] Bultman T. L. Leuchtmann A. (2008). Biology of the *Epichloë–Botanophila* interaction: An intriguing association between fungi and insects. *Fungal Biol. Rev.* 22 131–138. 10.1016/j.fbr.2009.04.003

[B47] Bultman T. L. Lembicz M. Leuchtmann A. (2022). Does the degree of mutualism between *Epichloë* fungi and *Botanophila* flies depend upon the reproductive mode of the fungi? *J. Fungi* 8 1270. 10.3390/jof8121270 36547603 PMC9781194

[B48] Bultman T. L. Leuchtmann A. Sullivan T. J. Dreyer A. P. (2011). Do Botanophila flies provide reproductive isolation between two species of *Epichloë* fungi? A field test. *New Phytol.* 190 206–212. 10.1111/j.1469-8137.2010.03612.x 21244433

[B49] Bultman T. L. Pulas C. Grant L. Bell G. Sullivan T. J. (2006). Effects of fungal endophyte isolate on performance and preference of bird cherry oat aphid. *Environ. Entomol.* 35 1690–1695. 10.1093/ee/35.6.1690

[B50] Bultman T. L. Welch A. M. Boning R. A. Bowdish T. I. (2000). The cost of mutualism in a fly-fungus interaction. *Oecologia* 124 85–90. 10.1007/s004420050027 28308416

[B51] Bush L. P. Fannin F. F. Siegel M. R. Dahlman D. L. Burton H. R. (1993). Chemistry, occurrence and biological effects of saturated pyrrolizidine alkaloids associated with endophyte-grass interactions. *Agric. Ecosyst. Environ.* 44 81–102. 10.1016/0167-8809(93)90040-V

[B52] Bush L. P. Wilkinson H. H. Schardl C. L. (1997). Bioprotective alkaloids of grass-fungal endophyte symbioses. *Plant Physiol.* 114 1–7. 10.1104/pp.114.1.1 12223685 PMC158272

[B53] Cabral D. Cafaro M. J. Saidman B. Lugo M. Reddy P. V. White J. F. Jr. (1999). Evidence supporting the occurrence of a new species of endophyte in some South American grasses. *Mycologia* 91 315–325. 10.1080/00275514.1999.12061021

[B54] Caldwell J. D. Coffey K. P. Jennings J. A. Philipp D. Young A. N. Tucker J. D. et al. (2013). Performance by spring and fall-calving cows grazing with full, limited, or no access to toxic *Neotyphodium coenophialum*-infected tall fescue. *J. Animal Sci.* 91 465–476. 10.2527/jas.2011-4603 22785163

[B55] Campbell M. A. Tapper B. A. Simpson W. R. Johnson R. D. Mace W. J. Ram A. et al. (2017). *Epichloë hybrida*, sp. nov., an emerging model system for investigating fungal allopolyploidy. *Mycologia* 109 715–729. 10.1080/00275514.2017.1406174 29370579

[B56] Caradus J. R. (2024). *Epichloë* fungal endophytes – a vital component for perennial ryegrass survival in New Zealand. *N. Z. J. Agric. Res.* 67 451–458. 10.1080/00288233.2023.2170426

[B57] Caradus J. R. Johnson L. J. (2020). *Epichloë* fungal endophytes – from a biological curiosity in wild grasses to an essential component of high performing ryegrass and fescue pastures. *J. Fungi* 6:322. 10.3390/jof6040322 33261217 PMC7720123

[B58] Caradus J. R. Card S. Finch S. C. Hume D. Johnson L. J. Mace W. J. et al. (2020). Ergot alkaloids in New Zealand pastures and their impact. *N. Z. J. Agric. Res.* 65 1–41. 10.1080/00288233.2020.1785514

[B59] Caradus J. R. Card S. Hewitt K. G. Hume D. Johnson L. (2021a). *Epichloë* fungi – obligate mutualists. *Encyclopedia* 1 1084–1100. 10.3390/encyclopedia1040083

[B60] Caradus J. R. Chapman D. Cookson T. Cotching B. Deighton M. Donnelly L. et al. (2021b). “*Epichloë* Endophytes – New Perspectives on a Key Ingredient for Resilient Perennial Grass Pastures,” in *Proceedings of the Resilient Pastures Symposium*, ed. Douglas G. B. (Hamilton: N.Z. Grassland Association Research and Practice Series). 10.33584/rps.17.2021.3435

[B61] Caradus J. R. Hume D. E. Popay A. J. Bonth A. C. M. Croy R. Forester N. et al. (2025). “AR128 - A New *Epichloë* Fungal Endophyte for Ryegrass,” in *Proceedings of the 11th International Symposium of Grass Microbial Endophytes*, eds Card S. Finch S. Popay A. (Hamilton: N.Z. Grassland Association Research and Practice Series), 10.33584/rps.18.2025.3771

[B62] Caradus J. R. Lovatt S. Belgrave B. (2013). Adoption of forage technologies, Proc. N.Z. *Grassland Assoc.* 75 39–44. 10.33584/jnzg.2013.75.2917

[B63] Card S. D. Bastías D. A. Caradus J. R. (2021). Antagonism to plant pathogens by *Epichloë* fungal endophytes-A review. *Plants* 10:1997. 10.3390/PLANTS10101997 34685806 PMC8539511

[B64] Card S. D. Bastías D. A. Zhang W. Hume D. E. Caradus J. R. (2024). *Epichloë* – a key element of New Zealand’s agricultural landscape. *N. Z. J. Bot.* 62 519–547. 10.1080/0028825X.2024.2331570

[B65] Card S. D. Faville M. J. Simpson W. R. Johnson R. D. Voisey C. R. de Bonth A. C. M. et al. (2014). Mutualistic fungal endophytes in the Triticeae – survey and description. *FEMS Microbiol. Ecol.* 88 94–106. 10.1111/1574-6941.12273 24754753

[B66] Card S. Christensen M. Ellison N. Faville M. Hume D. Johnson R. et al. (2025). “An *Epichloë* Endophyte Associated with the Afromontane grass *Festuca simensis*,” in *Proceedings of the 11th International Symposium of Grass Microbial Endophytes*, eds Card S. Finch S. Popay A. (Hamilton: N.Z. Grassland Association Research and Practice Series), 10.33584/rps.18.2025.3784

[B67] Carroll G. (1988). Fungal endophytes in stems and leaves: From latent pathogen to mutualistic symbiont. *Ecology* 69 2–9. 10.2307/1943154

[B68] Chacón-Fuentes M. León-Finalé G. Lizama M. Gutiérrez-Gamboa G. Martínez-Cisterna D. Quiroz A. et al. (2025). Induced defense in ryegrass–*Epichloë* symbiosis against *Listronotus bonariensis*: Impact on peramine levels and pest performance. *J. Fungi* 11:50. 10.3390/jof11010050 39852469 PMC11766347

[B69] Charlton N. D. Craven K. D. Afkhami M. E. Hall B. A. Ghimire S. R. Young C. A. (2014). Interspecific hybridization and bioactive alkaloid variation increases diversity in endophytic *Epichloë* species of Bromus laevipes. *FEMS Microbiol. Ecol.* 90 276–289. 10.1111/1574-6941.12393 25065688

[B70] Charlton N. D. Craven K. D. Mittal S. Hopkins A. A. Young C. A. (2012). *Epichloë canadensis*, a new interspecific epichloid hybrid symbiotic with Canada wildrye (*Elymus canadensis*). *Mycologia* 104 1187–1199. 10.3852/11-403 22675049

[B71] Cheplick G. P. Perera A. Koulouris K. (2000). Effect of drought on the growth of *Lolium perenne* genotypes with and without fungal endophytes. *Funct. Ecol.* 14 657–667. 10.1046/j.1365-2435.2000.00466.x

[B72] Christensen M. J. (1995). Variation in the ability of *Acremonium* endophytes of *Lolium perenne*, *Festuca arundinacea* and *F. pratensis* to form compatible associations in the three grasses. *Mycol. Res.* 99 466–470. 10.1016/S0953-7562(09)80647-3

[B73] Christensen M. J. (1996). Antifungal activity in grasses infected with *Acremonium* and *Epichloë* endophytes. *Australas. Plant Pathol.* 25 186–191. 10.1071/AP96032

[B74] Christensen M. J. Ball O. J.-P. Bennett R. J. Schardl C. L. (1997). Fungal and host genotype effects on compatibility and vascular colonization by *Epichloë festucae*. *Mycol. Res.* 101 493–501. 10.1017/S0953756296002833

[B75] Christensen M. J. Bennett R. J. Schmid J. (2002). Growth of *Epichloë/Neotyphodium* and p-endophytes in leaves of *Lolium* and *Festuca grasses*. *Mycol. Res.* 106 93–106. 10.1017/S095375620100510X

[B76] Christensen M. J. Bennett R. J. Ansari H. A. Koga H. Johnson R. D. Bryan G. T. et al. (2008). *Epichloë* endophytes grow by intercalary hyphal extension in elongating grass leaves. *Fungal Genet. Biol.* 45 84–93. 10.1016/j.fgb.2007.07.013 17919950

[B77] Christensen M. J. Simpson W. R. Al Samarrai T. (2000). Infection of tall fescue and perennial ryegrass plants by combinations of different *Neotyphodium* endophytes. *Mycol. Res.* 104 974–978. 10.1017/S0953756299002300

[B78] Christensen M. Latch G. (1991). Variation among isolates of *Acremonium endophytes* (*A. coenophialum* and possibly *A. typhinum*) from tall fescue (*Festuca arundinacea*). *Mycol. Res.* 95 1123–1126. 10.1016/S0953-7562(09)80558-3

[B79] Christensen M. Voisey C. (2007). “The Biology of the Endophyte/Grass Partnership,” in *Proceedings of the 6th International Symposium on Fungal Endophytes of Grasses*, eds Popay A. J. Thom E. R. (Christchurch: N.Z. Grassland Association Research and Practice Series). 10.33584/rps.13.2006.3078

[B80] Chung K. R. Schardl C. L. (1997). Vegetative compatibility between and within *Epichloë* species. *Mycologia* 89 558–565. 10.1080/00275514.1997.12026819

[B81] Chung K. R. Hollin W. Siegel M. R. Schardl C. L. (1997). Genetics of host specificity in *Epichloë typhina*. *Phytopathology* 87 599–605. 10.1094/PHYTO.1997.87.6.599 18945076

[B82] Chynoweth R. J. Pyke N. B. Rolston M. P. Kelly M. (2015). Trends in New Zealand herbage seed production: 2004-2014. *Proc. Agron. Soc. N. Z.* 45 47–56.

[B83] Clarke B. B. White J. F. Hurley R. H. Torres M. S. Sun S. Huff D. R. (2006). Endophyte-mediated suppression of dollar spot disease in fine fescues. *Plant Dis.* 90 994–998. 10.1094/PD-90-0994 30781289

[B84] Clay K. (1988). Fungal endophytes of grasses: A defensive mutualism between plants and fungi. *Ecology* 69 10–16. 10.2307/1943155

[B85] Clay K. (1990). Fungal endophytes of grasses. *Ann. Rev. Ecol. Syst.* 21 275–297. 10.1146/annurev.es.21.110190.001423

[B86] Clay K. Cheplick G. P. (1989). Effect of ergot alkaloids from fungal endophyte-infected grasses on fall armyworm (*Spodoptera frugiperda*). *J. Chem. Ecol.* 15 169–182. 10.1007/BF02027781 24271434

[B87] Clay K. Schardl C. (2002). Evolutionary origins and ecological consequences of endophyte symbiosis with grasses. *Amer. Nat.* 160 S99–S127. 10.1086/342161 18707456

[B88] Craven K. D. Hsiau P. T. W. Leuchtmann A. Hollin W. Schardl C. L. (2001). Multigene phylogeny of *Epichloë* species, fungal symbionts of grasses. *Ann. Mo. Bot. Gard* 88:14e34. 10.2307/2666129

[B89] Cross D. L. (1997). “Fescue Toxicosis in Horses,” in *Neotyphodium/Grass Interactions*, eds Bacon C. W. Hill N. S. (Boston, MA: Springer). 289–309. 10.1007/978-1-4899-0271-9_55

[B90] Cuyeu R. Rosso B. E. Pagano E. Soto G. Fox R. Ayub N. D. (2013). Genetic diversity in a world germplasm collection of tall fescue. *Gen. Mol. Biol.* 36 237–242. 10.1590/S1415-47572013005000021 23885206 PMC3715290

[B91] Decunta F. A. Pérez L. I. Malinowski D. P. Molina-Montenegro M. A. Gundel P. E. (2021). A systematic review on the effects of *Epichloë* fungal endophytes on drought tolerance in cool-season grasses. *Front. Plant Sci.* 12:644731. 10.3389/fpls.2021.644731 33841472 PMC8025668

[B92] DLF Seeds Australia (2026). *The Endophyte Experts.* Available online at: https://www.dlfseeds.com.au/innovations/endophytes (accessed May 6, 2026).

[B93] Duringer J. M. Blythe L. L. Estill C. T. Moon A. Murty L. Livesay S. et al. (2021). Determination of a sub-chronic threshold for lolitrem B and perennial ryegrass toxicosis in Angus cattle consuming endophyte-infected perennial ryegrass (*Lolium perenne*) straw over 64 days. *Livestock Sci.* 250:104570. 10.1016/j.livsci.2021.104570

[B94] Duringer J. M. Delorme M. J. M. Lehner A. Craig A. M. (2007). “A Review of the Ergot Alkaloids Found in Endophyte-Infected Tall Fescue and Perennial Ryegrass and their Metabolism after Ingestion by Livestock,” in *Proceedings of the 6th International Symposium on Fungal Endophyte of Grasses*, eds Popay A. J. Thom E. R. (Hamilton: N.Z. Grassland Association Research and Practice Series). 10.33584/rps.13.2006.3162

[B95] Dymock J. J. Prestidge R. A. Rowan D. D. (1989a). “The Effects of Lolitrem B on Argentine Stem Weevil Larvae,” in *Proceedings of the 42nd N.Z. Weed and Pest Control Conf*, (New Plymouth).

[B96] Dymock J. J. Rowan D. D. McGee I. R. (1989b). “Effects of Endophyte-produced Mycotoxins on Argentine Stem Weevil and the Cutworm *Graphania mutans*,” in *Proceedings of the 5th Australasian Conf. Grassland Invertebrate Ecology*, ed. Stahle P. P. (Melbourne).

[B97] Eady C. (2021). The impact of alkaloid-producing *Epichloë* endophyte on forage ryegrass breeding: A New Zealand perspective. *Toxins* 13:158. 10.3390/toxins13020158 33670470 PMC7922046

[B98] Easton H. S. (1999). “Ryegrass Endophyte: An Essential New Zealand Symbiosis,” in *Ryegrass Endophyte: An Essential N.Z. Symbiosis*, eds Woodfield D. R. Matthew C. (Hamilton: N.Z. Grassland Association Research and Practice Series), 10.33584/rps.7.1999.3384

[B99] Easton H. S. (2007). Grasses and *Neotyphodium* endophytes: Co-adaptation and adaptive breeding. *Euphytica* 154 295–306. 10.1007/s10681-006-9187-3

[B100] Easton H. S. Christensen M. J. Eerens J. P. J. Fletcher L. R. Hume D. E. Keogh R. G. et al. (2001). Ryegrass endophyte: A New Zealand Grassland success story. *Proc. Conference-New Zealand Grassland Assoc.* 63 37–46. 10.33584/jnzg.2001.63.2429

[B101] Eerens J. P. J. Lucas R. J. Easton S. White J. G. H. (1998). Influence of the endophyte (*Neotyphodium lolii*) on morphology, physiology, and alkaloid synthesis of perennial ryegrass during high temperature and water stress. *N. Z. J. Agric. Res.* 41 219–226. 10.1080/00288233.1998.9513305

[B102] Eichenseer H. Dahlman D. L. Bush L. P. (1991). Influence of endophyte infection, plant age and harvest interval on *Rhopalosiphum padi* survival and its relation to quantity of N-formyl and N-acetyl loline in tall fescue. *Entomol. Exp. Appl.* 60 29–38. 10.1111/j.1570-7458.1991.tb01519.x

[B103] Elbersen H. W. West C. P. (1996). Growth and water relations of field-grown tall fescue as influenced by drought and endophyte. *Grass Forage Sci.* 51 333–342. 10.1111/j.1365-2494.1996.tb02068.x

[B104] Elmi A. A. West C. P. (1995). Endophyte infection effects on stomatal conductance, osmotic adjustment and drought recovery of tall fescue. *New Phytol.* 131 61–67. 10.1111/j.1469-8137.1995.tb03055.x 33863166

[B105] Faeth S. H. Saari S. (2012). Fungal grass endophytes and arthropod communities: Lessons from plant defence theory and multitrophic interactions. *Fungal Ecol.* 5 364–371. 10.1016/j.funeco.2011.09.003

[B106] Faeth S. H. Sullivan T. (2003). Mutualistic asexual endophytes in a native grass are usually parasitic. *Am. Nat.* 161 310–325. 10.1086/345937 12675375

[B107] Ferguson J. Nichol W. Moorhead A. McKenzie S. (2025). “Impact of Selected *Epichloë* Endophyte on Perennial Ryegrass Yield Performance Across Regions in New Zealand,” in *Proceedings of the 11th International Symposium of Grass Microbial Endophytes*, eds Card S. Finch S. Popay A. (Hamilton: N.Z. Grassland Association Research and Practice Series), 10.33584/rps.18.2025.3783

[B108] Fernando K. Reddy P. Spangenberg G. C. Rochfort S. J. Guthridge K. M. (2022). Metabolic potential of *Epichloë* endophytes for host grass fungal disease resistance. *Microorganisms* 10:64. 10.3390/microorganisms10010064 35056512 PMC8781568

[B109] Fernando K. Reddy P. Vassiliadis S. Spangenberg G. C. Rochfort S. J. Guthridge K. M. (2021). The known antimammalian and insecticidal alkaloids are not responsible for the antifungal activity of *Epichloë* endophytes. *Plants* 10:2486. 10.3390/plants10112486 34834850 PMC8624124

[B110] Filho C. C. F. Andrade M. H. M. L. Nunes J. A. R. Wipff J. K. Hignight D. Rios E. F. et al. (2023). Breeding for drought tolerance in perennial ryegrass (*Lolium perenne* L.) and tall fescue (*Lolium arundinaceum* [Schreb.] Darbysh.) by exploring genotype by environment by management interactions. *Grassl. Res.* 2 22–36. 10.1002/glr2.12045

[B111] Finch S. C. Fletcher L. R. Babu J. V. (2012). The evaluation of endophyte toxin residues in sheep fat. *N. Z. Vet. J.* 60 56–60. 10.1080/00480169.2011.634746 22175431

[B112] Finch S. C. Munday J. S. Munday R. Kerby J. W. F. (2016a). Short term toxicity studies of loline alkaloids in mice. *Food Chem. Toxicol.* 94 243–249. 10.1016/j.fct.2016.06.002 27276360

[B113] Finch S. C. Pennell C. L. Kerby J. W. F. Cave V. M. (2016b). Mice find endophyte-infected seed of tall fescue unpalatable – implications for the aviation industry. *Grass Forage Sci.* 71 659–666. 10.1111/gfs.12203

[B114] Finch S. C. Munday J. S. Sprosen J. M. Bhattarai S. (2019). Toxicity studies of chanoclavine in mice. *Toxins* 11:249. 10.3390/toxins11050249 31052510 PMC6563201

[B115] Finch S. C. Munday J. S. Sutherland B. L. Vlaming J. B. Fletcher L. R. (2017). Further investigation of equine fescue oedema induced by Mediterranean tall fescue (*Lolium arundinaceum*) infected with selected fungal endophytes (*Epichloë coenophiala*) N.Z. *Vet. J.* 65 322–326. 10.1080/00480169.2017.1365660 28793837

[B116] Finch S. C. Prinsep M. R. Popay A. J. Wilkins A. L. Webb N. G. Bhattarai S. et al. (2020). Identification and structure elucidation of epoxyjanthitrems from *Lolium perenne* infected with the endophytic fungus *Epichloë festucae* var. *lolii* and determination of the tremorgenic and anti-insect activity of epoxyjanthitrem I. *Toxins* 12:526. 10.3390/toxins12080526 32824608 PMC7472112

[B117] Finch S. Thom E. Babu J. Hawkes A. Waugh C. (2013). The evaluation of fungal endophyte toxin residues in milk. *N. Z. Vet. J.* 61 11–17. 10.1080/00480169.2012.704626 22984816

[B118] Fletcher L. R. (1999). “Non-toxic Endophytes in Ryegrass and their Effect on Livestock Health and Production”, in *Ryegrass Endophyte: An Essential New Zealand Symbiosis*, eds Woodfield D. R Matthew C. (Hamilton: N.Z. Grassland Association Research and Practice Series). 10.33584/rps.7.1999.3393

[B119] Fletcher L. R. Harvey I. C. (1981). An association of *Lolium* endophyte with ryegrass staggers. *N. Z. Vet. J.* 29 185–186. 10.1080/00480169.1981.34839 6950332

[B120] Fletcher L. R. Finch S. C. Sutherland B. L. De Nicolo G. Mace W. J. Van Koten C. et al. (2017). The occurrence of ryegrass staggers and heat stress in sheep grazing ryegrass-endophyte associations with diverse alkaloid profiles. *N. Z. Vet. J.* 65 232–241. 10.1080/00480169.2017.1329673 28506113

[B121] Fletcher L. R. Markham L. J. White S. R. (1994). Endophytes and heat tolerance in lambs grazing perennial ryegrass. *Proc. N. Z. Grassl. Assoc.* 56 265–270. 10.33584/jnzg.1994.56.2116

[B122] Fletcher L. Sutherland B. Fletcher C. (1999). “The impact of endophyte on the health and productivity of sheep grazing ryegrass-based pastures,” in *Ryegrass Endophyte: An Essential NZ Symbiosis*, eds Woodfield D. R. Matthew C. (Napier: N.Z. Grassland Association Research and Practice Series), 10.33584/rps.7.1999.3403

[B123] Florea S. Schardl C. L. Hollin W. (2015). Detection and isolation of *Epichloë* species, fungal endophytes of grasses. *Curr. Protocols Microbiol.* 38 19A–11A. 10.1002/9780471729259.mc19a01s38 26237108

[B124] Forester T. Chettri P. Hudson D. Johnson R. Johnson L. (2025). “*Epichloë* is not Associated with the Pollen of Infected Ryegrass Plants,” in *Proceedings of the 11th International Symposium of Grass Microbial Endophytes*, eds Card S. Finch S. Popay A. (Hamilton: N.Z. Grassland Association Research and Practice Series), 10.33584/rps.18.2025.3764

[B125] Franzluebbers A. J. Seman D. H. Stuedemann J. A. (2009). Tall fescue persists and cattle perform well on a novel-endophyte association in the Southern Piedmont USA. *Forage Grazinglands* 7 1–8. 10.1094/FG-2009-0227-01-RS

[B126] Freeman E. M. (1903). The seed-fungus of *Lolium temulentum*. L., the Darnel. *Proc. Royal Society Lond.* 71 27–30. 10.1098/rspl.1902.0057

[B127] Freeman S. Rodriguez R. J. (1993). Genetic conversion of a fungal plant pathogen to a non-pathogenic, endophytic mutualist. *Science* 260 75–78. 10.1126/science.260.5104.75 17793536

[B128] Freitas P. (2017). *Crossing the Species Barrier: Investigating Vertical Transmission of a Fungal Endophyte from Tall Fescue within a Novel Ryegrass Association.* Ph.D. thesis, Lincoln: Lincoln University.

[B129] Fribourg H. A. Waller J. C. (2004). “*Neotyphodium* Research and Application in the USA,” in *Neotyphodium in Cool-Season Grasses*, eds Roberts C. A. West C. P. Spiers D. E. (Ames, IA: Blackwell Publishing).

[B130] Funk C. R. White R. H. Breen J. P. (1993). Importance of *Acremonium endophytes* in turf-grass breeding and management. *Agricult. Ecosyst. Environ.* 44 215–232. 10.1016/0167-8809(93)90048-T

[B131] Funk C. Halisky P. Johnson M. Siegel M. R. Stewart A. V. Ahmad S. et al. (1983). An endophytic fungus and resistance to sod webworms: Association in *Lolium perenne* L. *Nat. Biotechnol.* 1 189–191. 10.1038/nbt0483-189

[B132] Gadberry M. S. Denard T. M. Spiers D. E. Piper E. L. (2003). Effects of feeding ergovaline on lamb performance in a heat stress environment. *J. Animal Sci.* 81 1538–1545. 10.2527/2003.8161538x 12817502

[B133] Gagic M. Faville M. J. Zhang W. Forester N. T. Rolston M. P. Johnson R. D. et al. (2018). Seed transmission of *Epichloë* endophytes in *Lolium perenne* is heavily influenced by host genetics. *Front. Plant Sci.* 9:1580. 10.3389/fpls.2018.01580 30483280 PMC6242978

[B134] Gallagher R. T. Hawkes A. D. (1986). The potent tremorgenic neurotoxins lolitrem B and aflatrem: A comparison of the tremor response in mice. *Experientia* 42 823–825. 10.1007/BF01941539 3732493

[B135] Gallagher R. T. Campbell A. G. Hawkes A. D. Holland P. T. McGaveston D. A. Pansier E. A. (1982). Ryegrass staggers: The presence of lolitrem neurotoxins in perennial ryegrass seed. *N. Z. Vet. J.* 30 183–184. 10.1080/00480169.1982.34936 16030841

[B136] Gallagher R. T. Hawkes A. D. Steyn P. S. Vleggaar R. (1984). Tremorgenic neurotoxins from perennial ryegrass causing ryegrass staggers Disorder of livestock: Structure elucidation of lolitrem B. *J. Chem. Soc., Chem. Comm.* 1984 614–616. 10.1039/C39840000614

[B137] Gallagher R. T. White E. P. Mortimer P. H. (1981). Ryegrass staggers: Isolation of potent neurotoxins lolitrem A and lolitrem B from staggers-producing pastures. *N. Z. Vet. J.* 29 189–190. 10.1080/00480169.1981.34843 6950333

[B138] Gatenby W. A. Munday-Finch S. C. Wilkins A. L. Miles C. O. (1999). Terpendole M, a novel Indole- diterpenoid isolated from *Lolium perenne* infected with the endophytic fungus *Neotyphodium lolii*. *J. Agric. Food Chem.* 47 1092–1097. 10.1021/jf980767z 10552421

[B139] Gaynor D. Rowan D. (1985). “Peramine—An Argentine Stem Weevil Feeding Deterrent from Endophyte-Infected Ryegrass,” in *Proceedings of the 4th Australasian Conference on Grassland Invertebrate Ecology Canterbury*, (Canterbury).

[B140] Gentile A. Rossi M. S. Cabral D. Craven K. D. Schardl C. L. (2005). Origin, divergence, and phylogeny of *Epichloë* endophytes of native Argentine grasses. *Mol. Phylogenet. Evol.* 35 196–208. 10.1016/j.ympev.2005.01.008 15737591

[B141] Gerhards N. Neubauer L. Tudzynski P. Li S.-M. (2014). Biosynthetic pathways of ergot alkaloids. *Toxins* 6 3281–3295. 10.3390/toxins6123281 25513893 PMC4280535

[B142] Ghariani S. Charfeddine A. Amari M. Chakroun M. Neila T.-F. (2020). Interspecific molecular variation of Tunisian complex *Lolium perenne* L. and *Festuca arundinacea* Schreb. based on the Internal Transcribed Spacer locus (ITS). *Physiol. Mol. Biol. Plants* 26 331–339. 10.1007/S12298-019-00749-2 32158138 PMC7036405

[B143] Gilruth J. A. (1906). Meningoencephalitis (stomach staggers) in horses, cattle and sheep. *Ann. Rep. N. Z. Dep. Agricult.* 14 293–297.

[B144] Górzyńska K. (2025). “Living together” with *Epichloë*–Exploring the significance of non-plant partners in the fungal symbiotic network. *Fungal Biol. Rev.* 52:100429. 10.1016/j.fbr.2025.100429

[B145] Górzyńska K. Lembicz M. Olszanowski Z. Leuchtmann A. (2011). *Botanophila—Epichloë* interaction in a wild grass, *Puccinellia distans*, lacks dependence on the fly vector. *Ann. Entomol. Soc. Amer.* 104 841–846. 10.1603/AN11009

[B146] Guerre P. (2015). Ergot alkaloids produced by endophytic fungi of the genus *Epichloë*. *Toxins* 7 773–790. 10.3390/toxins7030773 25756954 PMC4379524

[B147] Gundel P. E. Omacini M. Sadras V. O. Ghersa C. M. (2010). The interplay between the effectiveness of the grass-endophyte mutualism and the genetic variability of the host plant. *Evol. Appl.* 3 538–546. 10.1111/J.1752-4571.2010.00152.X 25567945 PMC3352510

[B148] Gunter S. A. Beck P. A. (2004). Novel endophyte-infected tall fescue for growing beef cattle. *J. Anim. Sci.* 82(Suppl._13), E75–E82. 15471817 10.2527/2004.8213_supplE75x

[B149] Gwinn K. D. Gavin A. M. (1992). Relationship between endophyte infestation level of tall fescue seed lots and *Rhizoctonia zeae* seedling disease. *Plant Dis.* 76 911–914.

[B150] Hahn H. McManus M. T. Warnstorff K. Monahan B. J. Young C. A. Davies E. et al. (2008). *Neotyphodium* fungal endophytes confer physiological protection to perennial ryegrass (*Lolium perenne* L.) subjected to a water deficit. *Environ. Exp. Bot.* 63 183–199. 10.1016/j.envexpbot.2007.10.021

[B151] Hand M. L. Cogan N. O. Stewart A. V. Forster J. W. (2010). Evolutionary history of tall fescue morphotypes inferred from molecular phylogenetics of the *Lolium-Festuca* species complex. *BMC Evol. Boil.* 10:303. 10.1186/1471-2148-10-303 20937141 PMC2958922

[B152] Hannaway D. B. Fransen S. Cropper J. B. Teel M. Chaney T. Griggs T. D. et al. (1999). *Tall Fescue (Festuca arundinacea Schreb.).* PNW (Series), PNW0504, Washington State University Extension 04/1999. Available online at: https://rex.libraries.wsu.edu/esploro/outputs/report/Tall-Fescue/99900501846801842 (accessed July 03, 2026).

[B153] He L. Hatier J. H. B. Matthew C. (2017). Drought tolerance of two perennial ryegrass cultivars with and without AR37 endophyte. *N. Z. J. Agric. Res.* 60 173–188. 10.1080/00288233.2017.1294083

[B154] Hemken R. Jackson J. Jr. Boling J. (1984). Toxic factors in tall fescue. *J. Animal Sci.* 58 1011–1016. 10.2527/jas1984.5841011x 6373703

[B155] Hennessy L. M. Popay A. J. Finch S. C. Clearwater M. J. Cave V. M. (2016). Temperature and plant genotype alter alkaloid concentrations in ryegrass infected with an *Epichloë* endophyte and this affects an insect herbivore. *Front. Plant Sci.* 7:1097. 10.3389/fpls.2016.01097 27524991 PMC4966287

[B156] Hewitt K. G. Hofmann R. W. Ball O. J.-P. Cox N. Bryant R. H. Finch S. C. et al. (2024a). Root aphid (*Aploneura lentisci*) population size on perennial ryegrass is determined by drought and endophyte strain. *J. Pest. Sci.* 97 369–384. 10.1007/s10340-023-01630-8

[B157] Hewitt K. G. Hofmann R. W. Ball O. J.-P. Luo D. Finch S. C. Bryant R. H. et al. (2024b). Phosphorus fertiliser is associated with reduced grass grub (*Costelytra giveni*) fitness in *Epichloë* endophyte-infected meadow fescue and perennial ryegrass. *Pest. Manag. Sci.* 80 6409–6423. 10.1002/ps.8369 39162038

[B158] Hewitt K. G. Hofmann R. Ball O. Finch S. Bryant R. Popay A. (2025). “New Zealand’s Pastoral Ecosystems: Challenges, Solutions, and the Protective Role of *Epichloë* Endophytes,” in *Proceedings of the 11th International Symposium of Grass Microbial Endophytes*, eds Card S. Finch S. Popay A. (Hamilton: N.Z. Grassland Association Research and Practice Series). 10.33584/rps.18.2025.3761

[B159] Hewitt K. G. Popay A. J. Hofmann R. W. Caradus J. R. (2021). *Epichloë* - a lifeline for temperate grasses under combined drought and insect pressure. *Grass Res.* 1 1–11. 10.48130/GR-2021-0007

[B160] Hiroyuki K. Satoshi T. Shun-ichi T. Yoshihara T. Sakamura S. Shimanuki T. et al. (1989). New fungitoxic sesquiterpenoids, chokols A-G, from stromata of *Epichloë typhina* and the absolute configuration of chokol E. *Agric. Biol. Chem.* 53 789–796. 10.1080/00021369.1989.10869341

[B161] Hopkins A. A. Young C. A. Panaccione D. G. Simpson W. R. Mittal S. Bouton J. H. (2010). Agronomic performance and lamb health among several tall fescue novel endophyte combinations in the South-Central USA. *Crop Sci.* 50 1552–1561. 10.2135/cropsci2009.08.0473

[B162] Hoveland C. S. (1993). Importance and economic significance of the *Acremonium* endophytes to performance of animals and grass plant. *Agric. Ecosyst. Environ.* 44 3–12. 10.1016/0167-8809(93)90036-O

[B163] Hoveland C. S. (2009). “Origin and History,” in *Proceedings of the Tall Fescue for the Twenty-first Century*, eds Fribourg H. A. Hannaway D. B. West C. P. (Madison, WI), 1–10. 10.2134/agronmonogr53.c1

[B164] Hoveland C. S. Schmidt S. P. King C. C. Jr. Odom J. W. Clark E. M. McGuire J. A. et al. (1983). Steer performance and association of *Acremonium coenophialum* fungal endophyte on tall fescue pasture. *Agron. J.* 75 821–824. 10.2134/agronj1983.00021962007500050021x

[B165] Hudson D. Mace W. Popay A. Jensen J. McKenzie C. Cameron C. et al. (2021). Genetic manipulation of the ergot alkaloid pathway in *Epichloë* festucae var. *lolii* and its effect on black beetle feeding deterrence. *Toxins* 13:76. 10.3390/toxins13020076 33498584 PMC7909537

[B166] Hume D. E. (2018). “AR37-*Epichloë* Improves Rooting Mass and Depth of Perennial Ryegrass,” in *Proceedings of the 10th International Symposium on Fungal Endophyte of Grasses*, eds Vázquez de Aldana B. R. Zabalgogeazcoa I. (Salamanca).

[B167] Hume D. E. Sewell J. C. (2014). Agronomic advantages conferred by endophyte infection of perennial ryegrass (*Lolium perenne* L.) and tall fescue (*Festuca arundinacea* Schreb.) in Australia. *Crop Pasture Sci.* 65 747–757. 10.1071/CP13383

[B168] Hume D. E. Card S. D. Rolston M. P. (2013). “Effects of Storage Conditions on Endophyte and Seed Viability in Pasture Grasses,” in *Proceedings of the 22nd International Grassland Congress*, (Orange, NSW: Department of Primary Industry, Australia). Available online at: https://uknowledge.uky.edu/cgi/viewcontent.cgi?article=1182&context=igc

[B169] Hume D. E. Finch S. C. Stewart A. V. Caradus J. R. (2025). “Advances in *Epichloë* Endophyte plant Secondary Metabolites in Grasslands,” in *Advances in Temperate Grassland Science and Management*, ed. van den Pol-van Dasselaar A. (Cambridge: Burleigh Dodds Science Publishing). Available online at: https://www.taylorfrancis.com/reader/download/9a2c9c01-b0d1-4c2d-8657-5cdf1f2c10b0/chapter/pdf?context=ubx

[B170] Hume D. E. Ryan D. L. Cooper B. M. Popay A. J. (2007). Agronomic performance of AR37-infected ryegrass in northern New Zealand. *Proc. N. Z. Grassl. Assoc.* 69 201–205. 10.33584/jnzg.2007.69.2673

[B171] Hume D. E. Ryan G. D. Gibert A. Helander M. Mirlohi A. Sabzalian M. R. (2016). “*Epichloë* Fungal Endophytes for Grassland Ecosystems,” in *Sustainable Agriculture Reviews*, ed. Lichtfouse E. (Cham: Springer International Publishing). 10.1007/978-3-319-26777-7_6

[B172] Hume D. E. Stewart A. V. Simpson W. R. Johnson R. D. (2020). *Epichloë* fungal endophytes play a fundamental role in New Zealand grasslands. *J. Royal Society N. Z.* 50 279–298. 10.1080/03036758.2020.1726415

[B173] Hunt K. L. Sleper D. (1981). Fertility of hybrids between two geographic races of tall fescue. *Crop Sci.* 21 400–404. 10.2135/cropsci1981.0011183X002100030012x

[B174] Inda L. A. Sanmartín I. Buerki S. Catalán P. (2014). Mediterranean origin and miocene–holocene old world diversification of meadow fescues and ryegrasses (*Festuca subgenus Schedonorus* and *Lolium*). *J. Biogeography* 41 600–614. 10.1111/JBI.12211

[B175] Jackson A. P. A. (2004). reconciliation analysis of host switching in plant-fungal symbioses. *Evolution* 58 1909–1923. 10.1111/j.0014-3820.2004.tb00479.x 15521451

[B176] Jacobson D. R. Carr S. B. Hatton R. H. Buckner R. C. Graden A. P. Dowden D. R. et al. (1970). Growth, physiological responses, and evidence of toxicity in yearling dairy cattle grazing different grasses. *J. Dairy Sci.* 53 575–587. 10.3168/jds.S0022-0302(70)86256-7 5441911

[B177] Jensen J. G. Popay A. J. (2004). Perennial ryegrass infected with AR37 endophyte reduces survival of porina larvae. *N. Z. Plant Prot.* 57 323–328. 10.30843/nzpp.2004.57.6930

[B178] Jensen J. G. Popay A. J. (2007). “Reduction in Root Aphid Populations by Non-Toxic Endophyte Strains in Tall Fescue,” in *Proceedings of the 6th International Symposium on Fungal Endophytes of Grasses; Christchurch, New Zealand*, eds Popay A. J. Thom E. R. (Hamilton: N.Z. Grassland Association Research and Practice Series). 10.33584/rps.13.2006.3160

[B179] Jensen J. G. Miller T. A. Cave V. M. Johnson R. D. Scott B. Popay A. J. (2022). Two *Epichloë festucae* var. *lolii* endophytes in *Lolium perenne* reduce larval populations of the wheat sheath miner, Cerodontha australis. *Entomol. Experiment. Applicata* 170 145–157. 10.1111/eea.13115

[B180] Jensen J. Popay A. Tapper B. (2009). Argentine stem weevil adults are affected by meadow fescue endophyte and its loline alkaloids. *N. Z. Plant Prot.* 62 12–18. 10.30843/nzpp.2009.62.4800

[B181] Jia T. Oberhofer M. Shymanovich T. Faeth S. H. (2016). Effects of hybrid and non-hybrid *Epichloë* endophytes and their associated host genotypes on the response of a native grass to varying environments. *Microbial Ecol.* 72 185–196. 10.1007/S00248-016-0743-7 26909796

[B182] Johnson L. J. Caradus J. R. (2019). “The science required to deliver *Epichloë* endophytes to commerce,” in *Endophytes for a Growing World*, eds Hodkinson T. R. Doohan F. M. Saunders M. J. Murphy B. R. (Cambridge: Cambridge University Press).

[B183] Johnson L. J. de Bonth A. C. Briggs L. R. Caradus J. R. Finch S. C. Fleetwood D. J. et al. (2013). The exploitation of epichloae endophytes for agricultural benefit. *Fungal Diversity* 60 171–188. 10.1007/s13225-013-0239-4

[B184] Johnson L. J. Voisey C. R. Faville M. J. Moon C. D. Simpson W. R. Johnson R. D. et al. (2019). “Advances and Perspectives in Breeding for Improved Grass-Endophyte Associations,” in *Improving Sown Grasslands through Breeding and Management Proceedings of the Joint 20th Symposium of the European Grassland Federation and the 33rd Meeting of the EUCARPIA Section “Fodder Crops and Amenity Grasses”, Zürich*, eds Huguenin-Elie O. Studer B. Kölliker R. Reheul D. Probo M. Barre P. et al. (Zurich). 10.3929/ethz-b-000369264

[B185] Johnson M. C. Dahlman D. L. Siegel M. R. Bush L. P. Latch G. C. M. Potter D. A. et al. (1985). Insect feeding deterrents in endophyte-infected tall fescue. *Appl. Environ. Microbiol.* 49 568–571. 10.1128/aem.49.3.568-571.1985 16346751 PMC373550

[B186] Johnson R. D. Lane G. A. Koulman A. Cao M. Fraser K. Fleetwood D. J. et al. (2015). A novel family of cyclic oligopeptides derived from ribosomal peptide synthesis of an in planta-induced gene, gigA, in *Epichloë* endophytes of grasses. *Fungal Gen. Biol.* 85 14–24. 10.1016/j.fgb.2015.10.005 26519220

[B187] Ju Y. Sacalis J. N. Still C. C. (1998). Bioactive flavonoids from endophyte-infected bluegrass (*Poa ampla*). *J. Agric. Food Chem.* 46 3785–3788. 10.1021/jf980189m

[B188] Justus M. Witte L. Hartmann T. (1997). Levels and tissue distribution of loline alkaloids in endophyte-infected *Festuca pratensis*. *Phytochemistry* 44 51–57. 10.1016/S0031-9422(96)00535-3

[B189] Kane K. H. (2011). Effects of endophyte infection on drought stress tolerance of *Lolium perenne* accessions from the Mediterranean region. *Environ. Exp. Bot.* 71 337–344. 10.1016/j.envexpbot.2011.01.002

[B190] Kannadan S. Rudgers J. A. (2008). Endophyte symbiosis benefits a rare grass under low water availability. *Functional Ecol.* 22 706–713. 10.1111/j.1365-2435.2008.01395.x

[B191] Kaufononga S. A. F. (2019). *Indole Diterpenoid Secondary Metabolites Produced by the Epichloë festucae var. lolii – Lolium Perenne Symbiosis.* A thesis submitted in fulfilment of the requirements of the degree of Doctor of Philosophy in Chemistry, Hamilton: The University of Waikato. Available online at: https://researchcommons.waikato.ac.nz/server/api/core/bitstreams/42addaff-3776-4b5b-b811-0874880c4614/content

[B192] Kauppinen M. Saikkonen K. Helander M. Pirttilä A. M. Wäli P. R. (2016). *Epichloë* grass endophytes in sustainable agriculture. *Nat. Plants* 2:15224. 10.1038/nplants.2015.224 27249195

[B193] Kimmons C. A. Gwinn K. D. Bernard E. C. (1990). Nematode reproduction on endophyte-infected and endophyte-free tall fescue. *Plant Dis.* 74 757–761.

[B194] Kirby E. J. M. (1961). Host-parasite relations in the choke disease of grasses. *Trans. Br. Mycol. Soc.* 44 493–503. 10.1016/S0007-1536(61)80048-X

[B195] Klotz J. L. Bush L. P. Smith D. L. Shafer W. D. Smith L. L. Vevoda A. C. et al. (2006). Assessment of vasoconstrictive potential of D-lysergic acid using an isolated bovine lateral saphenous vein bioassay. *J. Animal Sci.* 84 3167–3175. 10.2527/jas.2006-038 17032812

[B196] Klotz J. L. Kirch B. H. Aiken G. E. Bush L. P. Strickland J. R. (2008). Effects of selected combinations of tall fescue alkaloids on the vasoconstrictive capacity of fescue-naïve bovine lateral saphenous veins. *J. Animal Sci.* 86 1021–1028. 10.2527/jas.2007-0576 18192563

[B197] Koga H. Christensen M. J. Bennett R. J. (1993). Incompatibility of some grass-*Acremonium* endophyte associations. *Mycol. Res.* 97 1237–1244. 10.1016/S0953-7562(09)81292-6

[B198] Kohlmeyer J. Kohlmeyer E. (1974). Distribution of *Epichloë typhina* (Ascomycetes) and its parasitic fly. *Mycologia* 66 77–86. 10.1080/00275514.1974.12019575

[B199] Koshino H. Terada S.-I. Yoshihara T. Sakamura S. Shimanuki T. Sato T. et al. (1988). Three phenolic acid derivatives from stromata of *Epichloë typhina* on *Phleum pratense*. *Phytochem.* 27 1333–1338. 10.1016/0031-9422(88)80188-2

[B200] Koshino H. Togiya S. Yoshihara T. Sakamura S. Shimanuki T. Sato T. et al. (1987). Four fungitoxic C-18 hydroxy unsaturated fatty acids from stromata of *Epichloë typhina*. *Tetrahedron. Lett.* 28 73–76. 10.1016/S0040-4039(00)95652-1

[B201] Koshino H. Yoshihara T. Okuno M. Sakamura S. Tajimi A. Shimanuki T. (1992). Gamahonolides A, B, and gamahorin, novel antifungal compounds from stromata of *Epichloë typhina* on *Phleum pratense*. *Biosci. Biotech. Biochem.* 56 1096–1099. 10.1271/bbb.56.1096 27286384

[B202] Koulman A. Lane G. A. Christensen M. J. Fraser K. Tapper B. A. (2007). Peramine and other fungal alkaloids are exuded in the guttation fluid of endophyte-infected grasses. *Phytochemistry* 68 355–360. 10.1016/j.phytochem.2006.10.012 17126863

[B203] Latch G. C. M. Christensen M. J. (1985). Artificial infection of grasses with endophytes. *Ann. Appl. Biol.* 107 17–24. 10.1111/j.1744-7348.1985.tb01543.x

[B204] Latch G. C. M. Christensen M. J. Gaynor D. L. (1985). Aphid detection of endophyte infection in tall fescue. *N. Z. J. Agric. Res.* 28 129–132. 10.1080/00288233.1985.10427006

[B205] Law R. (1985). “Evolution in a Mutualistic Environment,” in *The Biology of Mutualism*, ed. Boucher D. A. (London: Croom Helm), 145–170.

[B206] Layton D. L. Fletcher L. R. Litherland A. J. Scannell M. G. Sprosen J. Hoogendoorn C. J. et al. (2004). Effect of ergot alkaloids on liveweight gain and urine lysergol level in ewe hoggets and heifers. *Proc. N. Z. Soc. Anim. Prod.* 64 192–196. Available online at: https://www.nzsap.org/proceedings/2004/effect-ergot-alkaloids-liveweight-gain-and-urine-lysergol-level-ewe-hoggets-and

[B207] Lee K. C. de la Cerna C. C. Missaoui A. M. (2025). “Prospects of *Epichloë* Endophyte on Fall Armyworm Growth and Survival in Tall Fescue,” in *Proceedings of the 11th International Symposium of Grass Microbial Endophytes*, eds Card S. Finch S. Popay A. (Hamilton: N.Z. Grassland Association Research and Practice Series). 10.33584/rps.18.2025.3765

[B208] Lee K. Missaoui A. Mahmud K. Presley H. Lonnee M. (2021). Interaction between grasses and *Epichloë* endophytes and its significance to biotic and abiotic stress tolerance and the rhizosphere. *Microorgan* 9:21869. 10.3390/MICROORGANISMS9112186 34835312 PMC8623577

[B209] Lehtonen P. T. Helander M. Siddiqui S. A. Lehto K. Saikkonen K. (2006). Endophytic fungus decreases plant virus infections in meadow ryegrass (*Lolium pratense*). *Biol. Lett.* 2 620–623. 10.1098/rsbl.2006.0499 17148304 PMC1834004

[B210] Lenardon M. D. Munro C. A. Gow N. A. R. (2010). Chitin synthesis and fungal pathogenesis. *Curr. Opin. Microbiol.* 13 416–423. 10.1016/J.MIB.2010.05.002 20561815 PMC2923753

[B211] Leuchtmann A. (1992). Systematics, distribution, and host specificity of grass endophytes. *Nat. Toxins* 1 150–162. 10.1002/nt.2620010303 1344916

[B212] Leuchtmann A. Clay K. (1997). “The Population Biology of Grass Endophytes,” in *The Mycota, Part B, Plant Relationships*, eds Carroll G. C. Tudzynski P. (Berlin: Springer-Verlag), 185–202.

[B213] Leuchtmann A. Schardl C. L. (1998). Mating compatibility and phylogenetic relationships among two new species of *Epichloë* and other congeneric European species. *Mycol. Res.* 102 1169–1182. 10.1017/S0953756298006236

[B214] Leuchtmann A. Bacon C. W. Schardl C. L. White J. F. Jr. Tadych M. (2014). Nomenclatural realignment of *Neotyphodium species* with genus *Epichloë*. *Mycologia* 106 202–215. 10.3852/13-251 24459125

[B215] Leuchtmann A. Schardl C. L. Siegel M. R. (1994). Sexual compatibility and taxonomy of a new species of *Epichloë* symbiotic with fine fescue grasses. *Mycologia* 86 802–812. 10.1080/00275514.1994.12026487

[B216] Leuchtmann A. Young C. A. Stewart A. V. Simpson W. R. Hume D. E. Scott B. (2019). *Epichloe novae-zelandiae*, a new endophyte from the endemic New Zealand grass Poa matthewsii. *N. Z. J. Bot.* 57 271–288. 10.1080/0028825X.2019.1651344

[B217] Li C. J. Li F. Gou X. Y. Nan Z. B. (2008). “Effects of Abiotic Stresses on *Achnatherum inebrians* by Symbiotic Endophyte of *Neotyphodium gansuense*,” in *Multifunctional Grasslands in a Changing World*, ed. Organizing Committee of 2008 IGC/IRC Congress (Guangzhou: Guangdong People’s Publishing House). Available online at: https://uknowledge.uky.edu/cgi/viewcontent.cgi?article=3669&context=igc

[B218] Li F. Mei D. H. Ren T. Song Q. Y. (2023). Crude extracts and secondary metabolites of *Epichloë bromicola* against *Phytophthora infestans*. *Chem. Biodiversity* 20:e202200841. 10.1002/cbdv.202200841 36471540

[B219] Liu J. Nagabhyru P. Schardl C. L. (2017). *Epichloë* festucae endophytic growth in florets, seeds, and seedlings of perennial ryegrass (*Lolium perenne*). *Mycologia* 109 691–700. 10.1080/00275514.2017.1400305 29293414

[B220] Lowry D. Saucedo B. MacLean D. Oneil T. Propster J. R. Unger B. et al. (2010). Structural diversity among fungal hyphae: Insights into cell growth and phylogeny. *Microscopy Microanal.* 16 1148–1149. 10.1017/S1431927610058642

[B221] Lyons P. C. Plattner R. D. Bacon C. W. (1986). Occurrence of peptide and clavine ergot alkaloids in tall fescue grass. *Science* 232 487–489. 10.1126/science.3008328 3008328

[B222] Malinowski D. P. Belesky D. P. (1999). Tall fescue aluminium tolerance is affected by *Neotyphodium coenophialum* endophyte. *J. Plant Nutr.* 22 1335–1349. 10.1080/01904169909365716

[B223] Malinowski D. P. Belesky D. P. (2000). Adaptations of endophyte-infected cool-season grasses to environmental stresses: Mechanisms of drought and mineral stress tolerance. *Crop Sci.* 40 923–940. 10.2135/cropsci2000.404923x

[B224] Malinowski D. P. Belesky D. P. (2019). *Epichloe* (formerly *Neotyphodium*) fungal endophytes increase adaptation of cool-season perennial grasses to environmental stresses. *Acta Agrobotanica* 72:1767. 10.5586/aa.1767

[B225] Malinowski D. P. Belesky D. P. Lewis G. C. (2005). “Abiotic Stresses in Endophytic Grasses,” in *Neotyphodium in Cool-Season Grasses*, eds Roberts C. West C. P. Spiers D. (Oxford: Blackwell Publishing Ltd). Available online at: https://www.ars.usda.gov/research/publications/publication/?seqNo115=164279

[B226] Meijer G. Leuchtmann A. (1999). Multistrain infections of the grass *Brachypodium sylvaticum* by its fungal endophyte *Epichloë sylvatica*. *New Phytol.* 141 355–368. 10.1046/j.1469-8137.1999.00332.x 33862916

[B227] Merlet L. Dombrowski J. E. Bushman B. S. Gilmore B. S. Rivedal H. M. Martin R. C. (2022). Horizontal transmission and expression of *Epichloë typhina* in orchardgrass (*Dactylis glomerata*). *Eur. J. Plant Pathol.* 163 415–428. 10.1007/s10658-022-02485-y

[B228] Meyer W. A. Torres M. S. White J. F. Jr. (2013). “Biology and Applications of Fungal Endophytes in Turfgrasses,” in *Turfgrass: Biology, Use, and Management*, eds Stier J. C. Horgan B. P. Bonos S. A. (Madison, WI), 10.2134/agronmonogr56.c20

[B229] Miles C. O. Munday S. C. Wilkins A. L. Ede R. M. Towers N. R. (1994). Large-scale isolation of lolitrem B and structure determination of lolitrem E. *J. Agric. Food Chem.* 42 1488–1492. 10.1021/jf00043a018

[B230] Miles C. O. Uzal F. Garthwaite I. Munday-Finch S. C. di Menna M. E. (1995). “*Poa huecu* and *Festuca argentina*,” in *Toxinology and Food Safety Research Report*, ed. Garthwaite L. (Hamilton: AgResearch).

[B231] Milne G.D. (2007). “Technology transfer of novel ryegrass endophytes in New Zealand,” in *Proceedings of the 6th International Symposium on Fungal Endophytes of Grasses*, eds Popay A. J. Thom E. R. (Christchurch: N.Z. Grassland Association Research and Practice Series). 10.33584/rps.13.2006.3067

[B232] Mirzahosseini Z. Shabani L. Sabzalian M. R. Sharifi-Tehrani M. (2014). *Neotyphodium* endophytes may increase tolerance to Ni in tall fescue. *Eur. Soil. Biol.* 63 33–40. 10.1016/j.ejsobi.2014.05.004

[B233] MLA (2012). *Developing Increased Understanding, Awareness and Potential Mitigation Strategies for Perennial Ryegrass Toxicosis in Sheep Production Systems.* Available online at: https://www.mla.com.au/research-and-development/reports/2012/prgt-developing-increased-understanding-awareness-and-potential-mitigation-strategies-for-perennial-ryegrass-toxicosis-in-sheep-production-systems/#:~:text=The%20effects%20of%20PRGT%20can,in%20stocking%20rate%20was%20achieved (accessed March 28, 2026).

[B234] Moate P. J. Williams S. R. O. Grainger C. Hannah M. C. Mapleson D. Auldist M. J. et al. (2012). Effects of wild-type, AR1 and AR37 endophyte-infected perennial ryegrass on dairy production in Victoria, Australia. *Anim. Prod. Sci.* 52 1117–1130. 10.1071/AN12126

[B235] Mohammadi R. Nami Y. Tajaddod S. (2024). Biological pest control potential of symbiotic fungal endophytes of cool-season grasses. *Crop Prot.* 183:106751. 10.1016/j.cropro.2024.106751

[B236] Moon C. D. Craven K. D. Leuchtmann A. Clement S. L. Schardl C. L. (2004). Prevalence of interspecific hybrids among asexual fungal endophytes of grasses. *Mol. Ecol.* 13 1455–1467. 10.1111/j.1365-294X.2004.02138.x 15140090

[B237] Moon C. D. Guillaumin J. J. Ravel C. Li C. Craven K. D. Schardl C. L. (2007). New *Neotyphodium* endophyte species from the grass tribes Stipeae and Meliceae. *Mycologia* 99 895–905. 10.1080/15572536.2007.11832521 18333513

[B238] Moon C. D. Scott B. Schardl C. L. Christensen M. J. (2000). The evolutionary origins of *Epichloë* endophytes from annual ryegrasses. *Mycologia* 92 1103–1118. 10.1080/00275514.2000.12061258

[B239] Morgan-Jones G. Gams W. (1982). Notes on Hyphomycetes. XLI. An endophyte of *Festuca arundinacea* and the anamorph of *Epichloe typhina*, new taxa in one of two new sections of *Acremonium*. *Mycotaxon* 15 311–318.

[B240] Mortimer P. H. Young P. W. Di Menna M. E. (1984). Perennial ryegrass staggers research - an overview. *Proc. N. Z. Soc. Animal Prod.* 44 181–184. Available online at: https://www.nzsap.org/proceedings/1984/perennial-ryegrass-staggers-research-overview

[B241] Müller C. B. Krauss J. (2005). Symbiosis between grasses and asexual fungal endophytes. *Curr. Opin. Plant Boil.* 8 450–456. 10.1016/j.pbi.2005.05.007 15946893

[B242] Munday-Finch S. C. Miles C. O. Wilkins A. L. Hawkes A. D. (1995). Isolation and structure elucidation of lolitrem A, a tremorgenic mycotoxin from perennial ryegrass infected with *Acremonium lolii*. *J. Agric. Food Chem.* 43 1283–1288. 10.1021/jf00053a029

[B243] Munday-Finch S. C. Wilkins A. L. Miles C. O. (1998). Isolation of lolicine A, lolicine B, lolitriol, and lolitrem N from *Lolium perenne* infected with *Neotyphodium lolii* and evidence for the natural occurrence of 31-epilolitrem N and 31-epilolitrem F. *J. Agric. Food Chem.* 46 590–598. 10.1021/jf9706787 10554283

[B244] Munday-Finch S. C. Wilkins A. L. Miles C. O. Tomoda H. Ômura S. (1997). Isolation and structure elucidation of lolilline, a possible biosynthetic precursor of the lolitrem family of tremorgenic mycotoxins. *J. Agric. Food Chem.* 45 199–204. 10.1021/jf960396r

[B245] Nagabhyru P. Dinkins R. D. Wood C. L. Bacon C. W. Schardl C. L. (2013). Tall fescue endophyte effects on tolerance to water-deficit stress. *BMC Plant Biol.* 13:127. 10.1186/1471-2229-13-127 24015904 PMC3848598

[B246] Neill J. C. (1940). The endophyte of ryegrass (*Lolium perenne*). *N.Z. J. Sci. Technol.* 21 280A–291A.

[B247] Neill J. C. (1941). The endophytes of *Lolium* and *Festuca*. *N. Z. J. Sci. Technol.* 23 185A–193A.

[B248] Newman J. A. Gillis S. Hager H. A. (2021). Costs, benefits, parasites and mutualists: The use and abuse of the mutualism–parasitism continuum concept for *Epichloë* fungi. *Phil. Theory Practice Biol.* 14:9. 10.3998/ptpbio.2103

[B249] Niones J. T. Takemoto D. (2014). An isolate of *Epichloë festucae*, an endophytic fungus of temperate grasses, shows growth inhibitory activity to selective grass pathogens. *J. Gen. Plant Pathol.* 80 337–347. 10.1007/s10327-014-0521-7

[B250] Niones J. T. Takemoto D. (2015). VibA, a homologue of a transcription factor for fungal heterokaryon incompatibility, is involved in antifungal compound production in the plant symbiotic fungus *Epichloë festucae*. Eukaryot. *Cell* 14 13–24. 10.1128/ec.00034-14 24906411 PMC4279024

[B251] Noorifar N. Savoian M. S. Ram A. Lukito Y. Hassing B. Weikert T. W. et al. (2021). Chitin deacetylases are required for *Epichloë festucae* endophytic cell wall remodeling during establishment of a mutualistic symbiotic interaction with *Lolium perenne*. *Mol. Plant-Microbe Interact.* 34 1181–1192. 10.1094/MPMI-12-20-0347-R 34058838

[B252] NZ Veterinary Association (2007). *Research into Staggers Could Save Millions.* Available online at: https://www.scoop.co.nz/stories/BU0705/S00516/research-into-staggers-could-save-millions.htm#:~:text=Press%20Release:%20New%20Zealand%20Veterinary,industry%20$100%20million%20each%20year (accessed March 28, 2026).

[B253] Oberhofer M. Oberhofer M. Güsewell S. Leuchtmann A. (2014). Effects of natural hybrid and non-hybrid *Epichloë* endophytes on the response of *Hordelymus europaeus* to drought stress. *New Phytol.* 201 242–253. 10.1111/NPH.12496 24102453

[B254] Pagel L. Bultman T. L. Górzyńska K. Lembicz M. Leuchtmann A. Sangliana A. et al. (2019). *Botanophila flies*, vectors of *Epichloë* fungal spores, are infected by *Wolbachia*. *Mycology* 10 1–5. 10.1080/21501203.2018.1515119 30834147 PMC6394329

[B255] Pan J. Bhardwaj M. Nagabhyru P. Grossman R. B. Schardl C. L. (2014). Enzymes from fungal and plant origin required for chemical diversification of insecticidal loline alkaloids in grass-*Epichloë* Symbiota. *PLoS One* 9:e115590. 10.1371/journal.pone.0115590 25531527 PMC4274035

[B256] Panaccione D. G. Beaulieu W. T. Cook D. (2014). Bioactive alkaloids in vertically transmitted fungal endophytes. *Funct. Ecol.* 28 299–314. 10.1111/1365-2435.12076

[B257] Parish J. A. McCann M. A. Watson R. H. Paiva N. N. Hoveland C. S. Parks A. H. et al. (2003). Use of non-ergot alkaloid-producing endophytes for alleviating tall fescue toxicosis in stocker cattle. *J. Animal Sci.* 81 2856–2868. 10.2527/2003.81112856x 14601890

[B258] Patchett B. J. Chapman R. B. Fletcher L. R. Gooneratne S. R. (2008a). Endophyte-infected *Festuca pratensis* containing loline alkaloids deter feeding by *Listronotus bonariensis*. *N. Z. Plant Protect.* 61 205–209. 10.30843/nzpp.2008.61.6843

[B259] Patchett B. J. Chapman R. B. Fletcher L. R. Gooneratne S. R. (2008b). Root loline concentration in endophyte infected meadow fescue (*Festuca pratensis*) is increased by grass grub (*Costelytra zealandica*) attack. *N. Z. Plant Protect.* 61 210–214. 10.30843/nzpp.2008.61.6844

[B260] Patchett B. Gooneratne R. Chapman B. Fletcher B. (2011a). Effects of loline-producing endophyte-infected meadow fescue ecotypes on New Zealand grass grub (*Costelytra zealandica*). *N. Z. J. Agric. Res.* 54 303–313. 10.1080/00288233.2011.608686

[B261] Patchett B. Gooneratne R. Fletcher L. Chapman B. (2011b). Seasonal distribution of loline alkaloid concentration in meadow fescue infected with *Neotyphodium uncinatum*. *Crop Pasture Sci.* 62 603–609. 10.1071/CP11017

[B262] Patterson C. G. Potter D. A. Fannin F. F. (1991). Feeding deterrency of alkaloids from endophyte-infected grasses to Japanese beetle grubs. *Entomol. Exp. Appl.* 61 285–289. 10.1111/j.1570-7458.1991.tb01561.x

[B263] PBRA (2026a). *Endophyte Insect Control Ryegrass, Festulolium & Continental Tall Fescue.* Available online at: https://www.pbra.co.nz/forage-grasses-results/endophyte-insect-control-ryegrass-festulolium-continental-tall-fescue/ (accessed April 10, 2026).

[B264] PBRA (2026b). *NZPBRA Agreed Statement on Epichloë Endophyte Viability Testing.* Available online at: https://www.pbra.co.nz/scientific-papers/nzpbra-agreed-statement-on-epichloe-endophyte-viability-testing/ (accessed March 23, 2026).

[B265] Pennell C. G. L. Rolston M. P. (2013). “Avanex™ Unique Endophyte Technology - Bird deterrent endophytic grass for amenity turf and airports,” in *Proceedings of the 22nd International Grasslands Congress*, (Sydney, NSW).

[B266] Pennell C. G. L. Popay A. J. Ball O. J.-P. Hume D. E. Baird D. B. (2005). Occurrence and impact of pasture mealybug (*Balanococcus poae*) and root aphid (*Aploneura lentisci*) on ryegrass (*Lolium spp*.) with and without infection by *Neotyphodium* fungal endophytes. *N. Z. J. Agric. Res.* 48 329–337. 10.1080/00288233.2005.9513663

[B267] Pennell C. G. Popay A. J. Rolston M. P. Townsend R. J. Lloyd-West C. M. Card S. D. (2016). Avanex unique endophyte technology: Reduced insect food source at airports. *Environ. Entomol.* 45 101–108. 10.1093/ee/nvv145 26374758

[B268] Pennell C. Rolston M. Baird D. Hume D. McKenzie C. Card S. (2017a). Using novel-grass endophyte associations as an avian deterrent. *N. Z. Plant Protect.* 70 255–264. 10.30843/nzpp.2017.70.59

[B269] Pennell C. Rolston M. Koten C. Hume D. Card S. (2017b). Reducing bird numbers at New Zealand airports using a unique endophyte product. *N. Z. Plant Protect.* 70 224–234. 10.30843/nzpp.2017.70.55

[B270] Pennell C. Rolston M. de Bonth A. Simpson W. Hume D. (2010). Development of a bird-deterrent fungal endophyte in turf tall fescue. *N. Z. J. Agric. Res.* 53 145–150. 10.1080/00288231003777681

[B271] Pérez L. I. Gundel P. E. Zabalgogeazcoa I. Omacini M. (2020). An ecological framework for understanding the roles of *Epichloë* endophytes on plant defenses against fungal diseases. *Fungal Biol. Rev.* 34 115–125. 10.1016/J.FBR.2020.06.001

[B272] PGWS (2026). *AR37 the Future for Pasture.* Available online at: https://www.ar37endophyte.com/ (accessed May 6, 2026).

[B273] Philippe G. (2016). Lolitrem B and indole diterpene alkaloids produced by endophytic fungi of the genus *Epichloë* and their toxic effects in livestock. *Toxins* 8:47. 10.3390/toxins8020047 26891327 PMC4773800

[B274] Phillips T. D. Aiken G. E. (2009). Novel endophyte-infected tall fescues. *For. Grazinglands* 7 1–6. 10.1094/FG-2009-1102-01-RV

[B275] Popay A. J. Ball O. J.-P. (1998). “The Development of Fungal Endophytes as a Pest Management Tool for New Zealand Grasslands,” in *Proceedings of the 6th Australasian Applied Entomological Research Conference* (Brisbane, QLD).

[B276] Popay A. J. Cotching B. Moorhead A. Ferguson C. M. (2012). AR37 endophyte effects on porina and root aphid populations and ryegrass damage in the field. *Proc. N. Z. Grassl. Assoc.* 74 165–170. 10.33584/jnzg.2012.74.2856

[B277] Popay A. J. Cox N. R. (2016). *Aploneura lentisci* (Homoptera: Aphididae) and its interactions with fungal endophytes in perennial ryegrass (*Lolium perenne*). *Front. Plant Sci.* 7:1395. 10.3389/fpls.2016.01395 27695470 PMC5025451

[B278] Popay A. J. Gerard P. J. (2007). Cultivar and endophyte effects on a root aphid, *Aploneura lentisci*, in perennial ryegrass. *N. Z. Plant Prot.* 60 223–227. 10.30843/NZPP.2007.60.4624

[B279] Popay A.J. Hume D.E. Baltus J.G. Latch G.C.M. Tapper B.A. Lyons T.B. Cooper B.M. Pennell C.G. Eerens J.P.J. Marshall S.L. (1999). “Field Performance of Perennial Ryegrass (*Lolium perenne*) Infected with Toxin-Free Fungal Endophyte (*Neotyphodium spp*.),” in *Ryegrass Endophyte: An Essential New Zealand Symbiosis*, eds Woodfield D. R. Matthew C. (Hamilton: N.Z. Grassland Research and Practice Series). 10.33584/rps.7.1999.3388

[B280] Popay A. J. Hume D. E. Davies K. L. Tapper B. A. (2003a). Interactions between endophyte (*Neotyphodium spp*.) and ploidy in hybrid and perennial ryegrass cultivars and their effects on Argentine stem weevil (*Listronotus bonariensis*). *N. Z. J. Agric. Res.* 46 311–319. 10.1080/00288233.2003.9513559

[B281] Popay A. J. Hume D. E. Mace W. J. Faville M. J. Finch S. C. Cave V. (2021). A root aphid *Aploneura lentisci* is affected by *Epichloë* endophyte strain and impacts perennial ryegrass growth in the field. *Crop Pasture Sci.* 72 155–164. 10.1071/CP20299

[B282] Popay A. J. Hickey M. J. Stewart A. V. Hume D. E. (2008). “Potential Use of a Selected Strain of Fungal Endophyte for Insect Control in Turf Ryegrass,” in *Proceedings of the 1st European Turfgrass Society Conf*, (Pisa).

[B283] Popay A. J. Hume D. E. (2011). “Endophytes improve ryegrass persistence by controlling insects,” in *Pasture Persistence Symposium*, ed. Mercer C. F. (Hamilton: N.Z. Grassland Association Research and Practice Series).

[B284] Popay A. Jensen J. (2005). Soil biota associated with endophyte infected tall fescue in the field. *N. Z. Plant Prot.* 58 117–121. 10.30843/nzpp.2005.58.4282

[B285] Popay A. J. Jensen J. G. Cooper D. M. (2005). The effect of non-toxic endophytes in tall fescue on two major insect pests. *Proc. N. Z. Grassl. Assoc.* 71 169–173. 10.33584/jnzg.2005.67.2579

[B286] Popay A. J. Jensen J. G. Simpson W. R. Mace W. J. Somchit C. (2023). Translocation of loline alkaloids in *Epichloë*-infected cereal and pasture grasses: What the insects tell us. *J. Fungi* 9:96. 10.3390/jof9010096 36675917 PMC9865534

[B287] Popay A. J. Lane G. A. (2000). “The Effect of Crude Extracts Containing Loline Alkaloids on Two New Zealand Insect Pests,” in *Proceedings of the 4th International Neotyphodium/Grass Interactions Symposium.* eds Paul V. H. Dapprich P. D., Soest, Germany.

[B288] Popay A. J. Prestidge R. A. Rowan D. D. Dymock J. J. (1990). “The Role of *Acremonium lolii* Mycotoxins in Insect Resistance of Perennial Ryegrass (*Lolium perenne*),” in *Proceedings of the International Symposium on Acremonium/Grass Interactions*, eds Quisenberry S. S. Joost R. E.. (New Orleans, LA).

[B289] Popay A. J. Rijswijk K. Goldson S. L. (2017). Argentine stem weevil: Farmer awareness and the effectiveness of different ryegrass/endophyte associations. *J. N. Z. Grassl.* 79 153–158. 10.33584/jnzg.2017.79.570

[B290] Popay A. J. Tapper B. A. (2007). “Endophyte Effects on Consumption of Seed and Germinated Seedlings of Ryegrass and Fescue by Grass Grub (*Costelytra zealandica*) Larvae,” in *Proceedings of the 6th International Symposium on Fungal Endophytes of Grasses*, eds Popay A. J. Thom E. R. (Christchurch), 353–356.

[B291] Popay A. J. Tapper B. A. Podmore C. (2009). Endophyte-infected meadow fescue and loline alkaloids affect Argentine stem weevil larvae. *N. Z. Plant Prot.* 62 19–27. 10.30843/nzpp.2009.62.4801

[B292] Popay A. J. Thom E. R. (2009). Endophyte effects on major insect pests in Waikato dairy pasture. *Proc. N. Z. Grassl. Assoc.* 71 121–126. 10.33584/jnzg.2009.71.2758

[B293] Popay A. J. Townsend R. J. Fletcher L. R. (2003b). The effect of endophyte (*Neotyphodium uncinatum*) in meadow fescue on grass grub larvae. *N. Z. Plant Protect.* 56 123–128. 10.30843/nzpp.2003.56.6052

[B294] Popay A. J. Wyatt R. T. (1995). Resistance to Argentine stem weevil in perennial ryegrass infected with endophytes producing different alkaloids. *Proc. N. Z. Plant Prot. Conf.* 48 229–236. 10.30843/nzpp.1995.48.11487

[B295] Porter J. K. Thompson F. N. Jr. (1992). Effects of fescue toxicosis on reproduction in livestock. *J. Animal Sci.* 70 1594–1603. 10.2527/1992.7051594x 1526927

[B296] Porter J. K. Bacon C. W. Robbins J. D. (1979). Ergosine, ergosinine, and chanoclavine I from *Epichloe typhina*. *J. Agric. Food Chem.* 27 595–598. 10.1021/jf60223a045 447932

[B297] Pownall D. B. Lucas J. R. Familton A. S. Love B. G. Hines S. E. Fletcher L. R. (1993). The relationship between staggers and diarrhoea in lambs grazing different components of endophyte-infected ryegrass. *Proc. N. Z. Soc. Animal Prod.* 53 19–22. Available online at: https://www.nzsap.org/proceedings/1993/relationship-between-staggers-and-diarrhoea-lambs-grazing-different-components

[B298] Prestidge R. A. Gallagher R. T. (1985). “Lolitrem B - A Stem Weevil Toxin Isolated from *Acremonium*-infected Ryegrass,” in *Proceedings of the 38th N.Z. Weed and Pest Control Conference*, (Rotorua). 10.30843/nzpp.1985.38.9467

[B299] Prestidge R. A. Pottinger R. P. Barker G. M. (1982). “An Association of *Lolium endophyte* with Ryegrass Resistance to Argentine Stem Weevil,” in *Proceedings of the 35th N.Z. Weed and Pest Control Conf*, (Hamilton). 10.30843/nzpp.1982.35.10551

[B300] Purev E. Kondo T. Takemoto D. Niones J. T. Ojika M. (2020). Identification of ε-poly-l-lysine as an antimicrobial product from an *Epichloë* endophyte and isolation of fungal ε-PL synthetase gene. *Molecules* 25:1032. 10.3390/molecules25051032 32106587 PMC7179176

[B301] Pusztahelyi T. (2018). Chitin and chitin-related compounds in plant–fungal interactions. *Mycology* 9 189–201. 10.1080/21501203.2018.1473299 30181925 PMC6115883

[B302] Qawasmeh A. Obied H. K. Raman A. Wheatley W. (2012a). Influence of fungal endophyte infection on phenolic content and antioxidant activity in grasses: Interaction between *Lolium perenne* and different strains of *Neotyphodium lolii*. *J. Agric. Food Chem.* 60 3381–3388. 10.1021/jf204105k 22435921

[B303] Qawasmeh A. Raman A. Wheatley W. Nicol H. (2012b). Antioxidative capacity of phenolic compounds extracted from *Lolium perenne* and *Lolium arundinaceum* infected with *Neotyphodium* (Hypocreales: Clavicipitaceae). *Acta Physiol. Plant* 34 827–833. 10.1007/s11738-011-0878-6

[B304] Rao S. Baumann D. (2004). The interaction of a *Botanophila fly* species with an exotic *Epichloë fungus* in a cultivated grass: Fungivore or mutualist? *Entomol. Experiment. App.* 112 99–105. 10.1111/j.0013-8703.2004.00189.x

[B305] Read N. D. (2011). Exocytosis and growth do not occur only at hyphal tips. *Mol. Microbiol.* 81 4–7. 10.1111/J.1365-2958.2011.07702.X 21645129

[B306] Realini F. M. Escobedo V. M. Ueno A. C. Bastías D. A. Schardl C. L. Biganzoli F. et al. (2024). Anti-herbivory defences delivered by *Epichloë* fungal endophytes: A quantitative review of alkaloid concentration variation among hosts and plant parts. *Ann. Bot.* 133 509–520. 10.1093/aob/mcae014 38320313 PMC11037487

[B307] Reed K. F. M. (2005). “Pasture Ryegrass Toxins in Australian Pasture,” in *Perennial Ryegrass Toxicosis in Australia. Proceedings of the Symposium, Meat and Livestock Australia*, eds Reed K. F. M. Page S. W. Lean I. J. (Attwood, MI).

[B308] Reed K. F. M. Mace W. J. Walker L. V. Fletcher L. R. (2016). Endophyte metabolites associated with perennial ryegrass toxicosis. *Animal Prod. Sci.* 56 895–907. 10.1071/AN14495

[B309] Reid S. Hinds-Cook B. Stewart A. (2025). “Breeding Turfgrasses with *Epichloë* Endophytes for Abiotic and Biotic Stress Tolerance”, in *Proceedings of the 11th International Symposium of Grass Microbial Endophytes*, eds Card S. Finch S. Popay A. (Hamilton: N.Z. Grassland Research and Practice Series). 10.33584/rps.18.2025.3825

[B310] Ride J. P. Barber M. S. (1990). Purification and characterization of multiple forms of endochitinase from wheat leaves. *Plant Sci.* 71 185–197. 10.1016/0168-9452(90)90008-C

[B311] Riedell W. E. Kieckhefer R. E. Petroski R. J. Powell R. G. (1991). Naturally occurring and synthetic loline alkaloid derivatives: Insect feeding behavior modification and toxicity. *J. Entomol. Sci.* 26 122–129. 10.18474/0749-8004-26.1.122

[B312] Roberts C. Andrae J. (2004). Tall fescue toxicosis and management. *Crop Manag.* 3 1–18. 10.1094/CM-2004-0427-01-MG

[B313] Rolston M. Agee C. (2007). “Delivering Quality Seed to Specification-the USA and NZ Novel Endophyte Experience,” in *Proceedings of the 6th International Symposium on Fungal Endophytes of Grasses*, Vol. 13 eds Popay A. J. Thom E. R. (Christchurch: N.Z. Grassland Association Research and Practice Series), 229–231. 10.33584/rps.13.2006.3065

[B314] Rowan D. D. (1993). Lolitrems, peramine and paxilline: Mycotoxins of the ryegrass/endophyte interaction. *Agric. Ecosyst. Environ.* 44 103–122. 10.1016/0167-8809(93)90041-M

[B315] Rowan D. D. Gaynor D. L. (1986). Isolation of feeding deterrents against Argentine stem weevil from ryegrass infected with the endophyte *Acremonium loliae*. *J. Chem. Ecol.* 12 647–658. 10.1007/BF01012099 24306905

[B316] Rowan D. D. Dymock J. J. Brimble M. A. (1990). Effect of fungal metabolite peramine and analogs on feeding and development of Argentine stem weevil (*Listronotus bonariensis*). *J. Chem. Ecol.* 16 1683–1695. 10.1007/BF01014100 24263837

[B317] Rowan D. D. Hunt M. B. Gaynor D. L. (1986). Peramine, a novel insect feeding deterrent from ryegrass infected with the endophyte *Acremonium loliae*. *J. Chem. Soc. Lond. Chem. Commun.* 12 935–936. 10.1039/C39860000935 24306905

[B318] Rudgers J. A. Swafford A. L. (2009). Benefits of a fungal endophyte in *Elymus virginicus* decline under drought stress. *Basic Appl. Ecol.* 10 43–51. 10.1016/j.baae.2007.12.004

[B319] Saari S. Faeth S. H. (2012). Hybridization of *Neotyphodium endophytes* enhances competitive ability of the host grass. *New Phytol.* 195 231–236. 10.1111/j.1469-8137.2012.04140.x 22489964

[B320] Sabzalian M. R. Mirlohi A. (2010). *Neotyphodium* endophytes trigger salt resistance in tall and meadow fescues. *J. Plant Nutr. Soil. Sci.* 173 952–957. 10.1002/jpln.200900345

[B321] Saikia S. Parker E. J. Koulman A. Scott B. (2006). Four gene products are required for the fungal synthesis of the indole-diterpene, paspaline. *FEBS Lett.* 580 1625–1630. 10.1016/j.febslet.2006.02.008 16494875

[B322] Saikkonen K. Helander M. Faeth S. H. (2004a). “Fungal Endophytes: Hitchhikers of the Green World,” in *Plant Microbiology*, eds Gillings M. Holmes A. (Oxford: BIOS Scientific Publishers Limited). Available online at: https://www.taylorfrancis.com/chapters/edit/10.4324/9780203506608-5/fungal-endophytes-hitch-hikers-green-world-saikkonen-helander-faeth

[B323] Saikkonen K. Wali P. M. Faeth S. H. (2004b). Evolution of endophyte-plant symbioses. *Trends Plant Sci.* 9 275–280. 10.1016/j.tplants.2004.04.005 15165558

[B324] Saikkonen K. Young C. A. Helander M. Helander M. Schardl C. L. (2016). Endophytic *Epichloë* species and their grass hosts: From evolution to applications. *Plant Mol. Biol.* 90 665–675. 10.1007/S11103-015-0399-6 26542393 PMC4819788

[B325] Sampaio N. Gishen M. Reed K. Brown M. Gregory D. Munyard K. (2008). The occurrence and severity of grass toxicoses in Australian alpaca (*Vicugna pacos*) herds. *Aust. J. Exptal Agric.* 48 1099–1104. 10.1071/EA06325

[B326] Sampson K. (1933). The systemic infection of grasses by *Epichloe typhina* (Pers.) Tul. *Trans. Br. Mycol. Soc.* 18 30–47. 10.1016/S0007-1536(33)80025-8

[B327] Sampson K. (1935). The presence and absence of an endophytic fungus in *Lolium temulentum* and *L. perenne*. *Trans. Br. Mycol. Soc.* 19 337–343. 10.1016/S0007-1536(35)80031-4

[B328] Sampson K. (1937). Further observations on the systemic infection of *Lolium*. *Trans. Br. Mycol. Soc.* 21 84–97. 10.1016/S0007-1536(37)80007-8

[B329] Satognon F. (2024). Advancements in tall fescue (*Festuca arundinacea*) in the US: Origin, American introduction, development, and improvement. *J. Crop Impr.* 38 665–687. 10.1080/15427528.2024.2389456

[B330] Schardl C. L. (1996). *Epichloë* species: Fungal symbionts of grasses. *Ann. Rev. Phytopathol.* 34 109–130. 10.1146/annurev.phyto.34.1.109 15012537

[B331] Schardl C. L. (2001). *Epichloë festucae* and related mutualistic symbionts of grasses. *Fungal Genet. Boil.* 33 69–82. 10.1006/fgbi.2001.1275 11456460

[B332] Schardl C. L. Craven K. D. (2003). Interspecific hybridization in plant-associated fungi and oomycetes: A review. *Mol. Ecol.* 12 2861–2873. 10.1046/j.1365-294X.2003.01965.x 14629368

[B333] Schardl C. L. Leuchtmann A. (1999). Three new species of *Epichloë* symbiotic with North American grasses. *Mycologia* 91 95–107. 10.1080/00275514.1999.12060996

[B334] Schardl C. L. Tsai H. F. (1993). Molecular biology and evolution of the grass endophytes. *Nat. Toxins* 1 171–184. 10.1002/nt.2620010305 1344918

[B335] Schardl C. L. Craven K. D. Speakman S. Stromberg A. Lindstrom A. Yoshida R. (2008). A novel test for host-symbiont codivergence indicates ancient origin of fungal endophytes in grasses. *Syst. Biol.* 57 483–498. 10.1080/10635150802172184 18570040

[B336] Schardl C. L. Grossman R. B. Nagabhyru P. Faulkner J. R. Mallik U. P. (2007). Loline alkaloids: Currencies of mutualism. *Phytochemistry* 68 980–996. 10.1016/j.phytochem.2007.01.010 17346759

[B337] Schardl C. L. Leuchtmann A. Spiering M. J. (2004). Symbioses of grass with seedborne fungal endophytes. *Ann. Rev. Plant Biol.* 55 315–340. 10.1146/annurev.arplant.55.031903.141735 15377223

[B338] Schardl C. L. Leuchtmann A. Chung K. R. Penny D. Siegel M. R. (1997). Coevolution by common descent of fungal symbionts (*Epichloë spp*.) and grass hosts. *Mol. Biol. Evol.* 14 133–143. 10.1093/oxfordjournals.molbev.a025746

[B339] Schardl C. L. Leuchtmann A. Tsai H.-F. Collett M. A. Watt D. M. Scott D. B. (1994). Origin of a fungal symbiont of perennial ryegrass by interspecific hybriization of a mutualist with the ryegrass choke pathogen, *Epichloë typhina*. *Genetics* 136 1307–1317. 10.1093/genetics/136.4.1307 8013907 PMC1205911

[B340] Schardl C. L. Panaccione D. G. Tudzynski P. (2006). Ergot alkaloids–biology and molecular biology. *Alkaloids: Chem. Biol.* 63 45–86. 10.1016/s1099-4831(06)63002-2 17133714

[B341] Schardl C. L. Scott B. Florea S. Zhang D. (2009). “*Epichloë* Endophytes: Clavicipitaceous Symbionts of Grasses,” in *Plant Relationships*, 2nd Edn, ed. Deising V. H. (Berlin: Springer-Verlag).

[B342] Schardl C. L. Young C. A. Faulkner J. R. Florea S. Pan J. (2012). Chemotypic diversity of epichloae, fungal symbionts of grasses. *Fungal Ecol.* 5 331–344. 10.1016/j.funeco.2011.04.005

[B343] Schardl C. L. Young C. A. Hesse U. Amyotte S. G. Andreeva K. Calie P. J. et al. (2013a). Plant symbiotic fungi as chemical engineers: Multi-genome analysis of the Clavicipitaceae reveals dynamics of alkaloid loci. *PLoS Genet.* 9:e1003323. 10.1371/journal.pgen.1003323 23468653 PMC3585121

[B344] Schardl C. L. Young C. A. Pan J. Florea S. Takach J. E. Panaccione D. G. et al. (2013b). Currencies of mutualisms: Sources of alkaloid genes in vertically transmitted Epichloae. *Toxins* 5 1064–1088. 10.3390/toxins5061064 23744053 PMC3717770

[B345] Schirrmann M. K. Zoller S. Croll D. Stukenbrock E. H. Leuchtmann A. Fior S. (2018). Genomewide signatures of selection in *Epichloë* reveal candidate genes for host specialization. *Mol. Ecol.* 27 3070–3086. 10.1111/mec.14585 29633410

[B346] Schirrmann M. K. Zoller S. Fior S. Leuchtmann A. (2015). Genetic evidence for reproductive isolation among sympatric *Epichloë* endophytes as inferred from newly developed microsatellite markers. *Microb. Ecol.* 70 51–60. 10.1007/S00248-014-0556-5 25542204

[B347] Schmidt D. (1993). “Effects of *Acremonium uncinatum* and a Phialophora-like Endophyte on Vigour, Insect and Disease Resistance of Meadow Fescue,” in *Proceedings of the 2nd International Symposium on Acremonium/grass Interactions*, eds Hume D. E. Latch G. C. M. Easton H. S. (Palmerston North).

[B348] Schulz B. Boyle C. (2005). The endophytic continuum. *Mycol. Res.* 109 661–686. 10.1017/S095375620500273X 16080390

[B349] Schumann B. Lebzien P. Ueberschar K.-H. Dänicke S. (2009). Effects of the level of feed intake and ergot contaminated concentrate on ergot alkaloid metabolism and carry over into milk. *Mol. Nutr. Food Res.* 53 931–938. 10.1002/mnfr.200800319 19685550

[B350] Scott B. Green K. Berry D. (2018). The fine balance between mutualism and antagonism in the *Epichloë* festucae–grass symbiotic interaction. *Curr. Opin. Plant Biol.* 44 32–38. 10.1016/j.pbi.2018.01.010 29454183

[B351] Selosse M.-A. Schardl C. L. (2007). Fungal endophytes of grasses: Hybrids rescued by vertical transmission? An evolutionary perspective. *New Phytol.* 173 452–458. 10.1111/j.1469-8137.2007.01978.x 17244040

[B352] Seman D. H. Stuedemann J. A. Breedlove D. L. Wilkinson S. R. Belesky D. P. Thompson F. N. et al. (1990). “Differences in Grazing Behaviour of Steers Consuming Endophyte Infected and Non-Infected Tall Fescue,” in *Proceedings of the International Symposium Acremonium/Grass Interactions*, eds Quisenberry S. S. Joost R. E. Baton R. E. (Rouge: Louisiana Agricultural Experimental Station).

[B353] Servedio M. R. Noor M. A. F. (2003). The role of reinforcement in speciation: Theory and data. *Annu. Rev. Ecol. Evol. Syst.* 34 339–364. 10.1146/annurev.ecolsys.34.011802.132412

[B354] Seto Y. Takahashi K. Matsuura H. Kogami Y. Yada H. Yoshihara T. et al. (2007). Novel cyclic peptide, epichlicin, from the endophytic fungus, *Epichloe typhina*. *Biosci. Biotech. Biochem.* 71 1470–1475. 10.1271/bbb.60700 17587677

[B355] Shiba T. Sugawara K. (2009). Fungal loline alkaloids in grass endophyte associations confer resistance to the rice leaf bug, *Trigonotylus caelestialium*. *Entomol. Exp. Appl.* 130 55–62. 10.1111/j.1570-7458.2008.00791.x

[B356] Shymanovich T. Charlton N. D. Musso A. M. Scheerer J. R. Cech N. B. Faeth S. H. et al. (2017). Interspecific and intraspecific hybrid *Epichloë* species symbiotic with the North American native grass *Poa alsodes*. *Mycologia* 109 459–474. 10.1080/00275514.2017.1340779 28723242

[B357] Shymanovich T. Musso A. M. Cech N. B. Faeth S. H. (2019). *Epichloë* endophytes of *Poa alsodes* employ alternative mechanisms for host defence: Insecticidal versus deterrence. *Arthropod-Plant Interact.* 13 79–90. 10.1007/S11829-018-9635-8

[B358] Shymanovich T. Saari S. Lovin M. E. Jarmusch A. K. Jarmusch S. A. Musso A. M. et al. (2015). Alkaloid variation among epichloid endophytes of sleepygrass (*Achnatherum robustum*) and consequences for resistance to insect herbivores. *J. Chem. Ecol.* 41 93–104. 10.1007/s10886-014-0534-x 25501262

[B359] Siegel M. R. Bush L. P. (1996). “Defensive Chemicals in Grass-Fungal Endophyte Associations,” in *Phytochemical Diversity and Redundancy in Ecological Interactions. Recent Advances in Phytochemistry*, Vol. 30 eds Romeo J. T. Saunders J. A. Barbosa P. (Boston, MA: Springer), 10.1007/978-1-4899-1754-6_4

[B360] Siegel M. R. Latch G. C. M. (1991). Expression of antifungal activity in agar culture by isolates of grass endophytes. *Mycologia* 83 529–537. 10.2307/3760368

[B361] Siegel M. R. Latch G. C. M. Bush L. P. Fannin F. F. Rowan D. D. Tapper B. A. et al. (1990). Fungal endophyte-infected grasses: Alkaloid accumulation and aphid response. *J. Chem. Ecol.* 16 3301–3315. 10.1007/BF00982100 24263431

[B362] Simpson W. R. Faville M. J. Moraga R. A. Williams W. M. McManus M. T. Johnson R. J. (2014). *Epichloë* fungal endophytes and the formation of synthetic symbioses in Hordeeae (=Triticeae) grasses. *J. Syst. Evol.* 52 794–806. 10.1111/jse.12107

[B363] Smith B. Julian A. F. (1998). Perennial ryegrass staggers. *Surveillance* 25 3–5.

[B364] Smith S. Phillips T. D. (2016). *Novel Endophyte Varieties: What’s the Difference?.* Available online at: https://uknowledge.uky.edu/forage_kca/2016/Session/4/ (accessed March 22, 2026).

[B365] Song H. Song Q.-Y. Li X. Z. Nan Z. (2015). Are *Epichloë* endophytes specific to Elymus grass hosts. *Gen. Mol. Res.* 14 17463–17471. 10.4238/2015.DECEMBER.21.17 26782389

[B366] Song Q. Y. Nan Z. B. Gao K. Song H. Tian P. Zhang X. X. et al. (2015). Antifungal, phytotoxic, and cytotoxic activities of metabolites from *Epichloë* bromicola, a fungus obtained from *Elymus tangutorum* grass. *J. Agric. Food Chem.* 63 8787–8792. 10.1021/acs.jafc.5b04260 26395226

[B367] Song Q. Y. Yu H. T. Zhang X. X. Nan Z. B. Gao K. (2017). Dahurelmusin A, a hybrid peptide–polyketide from *Elymus dahuricus* Infected by the *Epichloë bromicola* endophyte. *Organ. Lett.* 19 298–300. 10.1021/acs.orglett.6b03568 28029264

[B368] Sonnenberg C. B. Haugen P. (2023). Bipartite genomes in enterobacterales: Independent origins of chromids, elevated openness and donors of horizontally transferred genes. *Int. J. Mol. Sci.* 24:4292. 10.3390/ijms24054292 36901726 PMC10002438

[B369] Spiering M. J. Davies E. Tapper B. A. Schmid J. Lane G. A. (2002). Simplified extraction of ergovaline and peramine for analysis of tissue distribution in endophyte-infected grass tillers. *J. Agric. Food Chem.* 50 5856–5862. 10.1021/jf025602b 12358450

[B370] Spiering M. J. Lane G. A. Christensen M. J. Schmid J. (2005). Distribution of the fungal endophyte *Neotyphodium lolii* is not a major determinant of the distribution of fungal alkaloids in *Lolium perenne* plants. *Phytochemistry* 66 195–202. 10.1016/j.phytochem.2004.11.021 15652576

[B371] Steinebrunner F. Schiestl F. P. Leuchtmann A. (2008a). Ecological role of volatiles produced by *Epichloë*: Differences in antifungal toxicity. *FEMS Microbiol. Ecol.* 64 307–316. 10.1111/j.1574-6941.2008.00452.x 18328083

[B372] Steinebrunner F. Twele R. Francke W. Leuchtmann A. Schiestl F. P. (2008b). Role of odour compounds in the attraction of gamete vectors in endophytic *Epichloë* fungi. *New Phytol.* 178 401–411. 10.1111/j.1469-8137.2007.02347.x 18194147

[B373] Stewart A. V. (2006). “Genetic Origins of Perennial Ryegrass (*Lolium perenne*) for New Zealand Pastures,” in *Advances in Plant Breeding*, Vol. 12 ed. Mercer C. F. (Hamilton: N.Z. Grassland Association Research and Practice Series), 55–61. 10.33584/RPS.12.2006.3042

[B374] Stewart A. V. Barcellos G. Brilman L. (2022). Use of endophytic fungi in turfgrasses: Difficulties in delivery to the market. *Intern. Turfgrass Soc. Res. J.* 14 1070–1073. 10.1002/its2.131

[B375] Stewart A. V. Hume D. E. Harrison F. (2025). “Meadow Fescue and its *Epichloë* Endophytes in New Zealand,” in *Proceedings of the 11th International Symposium of Grass Microbial Endophytes*, eds Card S. Finch S. Popay A. (Hamilton: N.Z. Grassland Association Research and Practice Series). 10.33584/rps.18.2025.3767

[B376] Strickland J. R. Aiken G. E. Spiers D. E. Fletcher L. R. Oliver J. W. (2009). “Physiological basis of fescue toxicosis,” in *Tall Fescue for the Twenty-first Century*, eds Fribourg H. A. Hannaway D. B. West C. P. (Madison, WI), 10.2134/agronmonogr53.c12

[B377] Stuedemann J. A. Hoveland C. S. (1988). Fescue endophyte: History and impact on animal agriculture. *J. Prod. Agric.* 1 39–44. 10.2134/jpa1988.0039

[B378] Stuedemann J. A. Breedlove D. L. Pond K. R. Belesky D. D. Tate L. P. Thompson F. N. et al. (1989). “Effect of Endophyte (*Acremonium coenophialum*) Infection of Tall Fescue and Paddock Exchange on Intake and Performance of Grazing Steers,” in *Proceedings of the XVI International Grassland Congress*, Nice, France. Available online at: https://uknowledge.uky.edu/cgi/viewcontent.cgi?article=9096&context=igc

[B379] Tadych M. Ambrose K. V. Bergen M. S. Belanger F. C. White J. F. Jr. (2012). Taxonomic placement of *Epichloë poae* sp. nov. and horizontal dissemination to seedlings via conidia. *Fungal Diversity* 54 117–131. 10.1007/s13225-012-0170-0

[B380] Tadych M. Bergen M. S. White J. F. (2014). *Epichloë* spp. associated with grasses: New insights on life cycles, dissemination and evolution. *Mycologia* 106 181–201. 10.3852/106.2.181 24877257

[B381] Tanaka A. Christensen M. J. Takemoto D. Park P. Scott B. (2006). Reactive oxygen species play a role in regulating a fungus–perennial ryegrass mutualistic interaction. *Plant Cell* 18 1052–1066. 10.1105/TPC.105.039263 16517760 PMC1425850

[B382] Tanaka A. Tapper B. A. Popay A. Parker E. J. Scott B. (2005). A symbiosis expressed non-ribosomal peptide synthetase from a mutualistic fungal endophyte of perennial ryegrass confers protection to the symbiotum from insect herbivory. *Mol. Microbiol.* 57 1036–1050. 10.1111/j.1365-2958.2005.04747.x 16091042

[B383] Tapper B. A. Lane G. A. (2004). “Janthitrems Found in a *Neotyphodium endophyte* of Perennial Ryegrass,” in *Proceedings of the 5th International Symposium on Neotyphodium/Grass interactions (Poster 301)*, (Fayetteville, AR: University of Arkansas Press).

[B384] Thom E. R. Popay A. J. Hume D. E. Fletcher L. R. (2012). Evaluating the performance of endophytes in farm systems to improve farmer outcomes – a review. *Crop Pasture Sci.* 63 927–943. 10.1071/CP12152

[B385] Thom E. R. Popay A. J. Waugh C. D. Minneé E. M. K. (2014). Impact of novel endophytes in perennial ryegrass on herbage production and insect pests from pastures under dairy cow grazing in northern New Zealand. *Grass Forage Sci.* 69 191–204. 10.1111/gfs.12040

[B386] Thom E. R. Waugh C. D. Minneé E. M. K. Waghorn G. C. (2013). Effects of novel and wild-type endophytes in perennial ryegrass on cow health and production. *N. Z. Vet. J.* 61 87–97. 10.1080/00480169.2012.715379 22913546

[B387] Thünen T. Becker Y. Cox M. P. Ashrafi S. (2022). *Epichloë scottii* sp. nov., a new endophyte isolated from *Melica uniflora* is the missing ancestor of *Epichloë disjuncta*. *IMA Fungus* 13:2. 10.1186/s43008-022-00088-0 35109929 PMC8812020

[B388] Tian Z. Wang R. Ambrose K. V. Clarke B. B. Belanger F. C. (2017). The *Epichloë festucae* antifungal protein has activity against the plant pathogen *Sclerotinia homoeocarpa*, the causal agent of dollar spot disease. *Sci. Rep.* 7:5643. 10.1038/s41598-017-06068-4 28717232 PMC5514056

[B389] Tor-Agbidye J. Blythe L. L. Craig A. M. (2001). Correlation of endophyte toxins (ergovaline and lolitrem B) with clinical disease: Fescue foot and perennial ryegrass staggers. *Vet. Hum. Toxicol.* 43 140–146. Available online at: https://pubmed.ncbi.nlm.nih.gov/11383653/11383653

[B390] Treindl A. D. Leuchtmann A. (2019). Assortative mating in sympatric ascomycete fungi revealed by experimental fertilizations. *Fungal Biol.* 123 676–686. 10.1016/j.funbio.2019.06.005 31416587

[B391] Treindl A. D. Stapley J. Winter D. J. Cox M. P. Leuchtmann A. (2021). Chromosome-level genomes provide insights into genome evolution, organization and size in *Epichloe* fungi. *Genomics* 113 4267–4275. 10.1016/J.YGENO.2021.11.009 34774981

[B392] Tsai H. F. Liu J. S. Staben C. Christensen M. J. Latch G. C. Siegel M. R. et al. (1994). Evolutionary diversification of fungal endophytes of tall fescue grass by hybridization with *Epichloë species*. *Proc. Natl. Acad. Sci. U. S. A.* 91 2542–2546. 10.1073/pnas.91.7.2542 8172623 PMC43405

[B393] van Heeswijck R. McDonald G. (1992). *Acremonium* endophytes in perennial ryegrass and other pasture grasses in Australia and New Zealand. *Aust. J. Agric. Res.* 43 1683–1709. 10.1071/AR9921683

[B394] Vassiliadis S. Reddy P. Hemsworth J. Spangenberg G. C. Guthridge K. M. Rochfort S. J. (2023). Quantitation and distribution of *Epichloë*-derived alkaloids in perennial ryegrass tissues. *Metabolites* 13:205. 10.3390/metabo13020205 36837825 PMC9966479

[B395] Voisey C. R. (2010). Intercalary growth in hyphae of filamentous fungi. *Fungal Biol. Rev.* 24 123–131. 10.1016/j.fbr.2010.12.001

[B396] von Cräutlein M. Helander M. Korpelainen H. Leinonen P. H. Vázquez, de Aldana B. R. et al. (2021). Genetic diversity of the symbiotic fungus *Epichloë festucae* in naturally occurring host grass populations. *Front. Microbiol.* 12:756991. 10.3389/fmicb.2021.756991 34925265 PMC8678516

[B397] Wang Z. Li C. White J. (2020). Effects of *Epichloë* endophyte infection on growth, physiological properties and seed germination of wild barley under saline conditions. *J. Agro. Crop Sci.* 206 43–51. 10.1111/jac.12366

[B398] Weedon C. M. Mantle P. G. (1987). Paxilline biosynthesis by *Acremonium loliae*; a step towards defining the origin of lolitrem neurotoxins. *Phytochemistry* 26 969–971. 10.1016/S0031-9422(00)82327-4

[B399] West C. P. (1994). “Physiology and drought tolerance of endophyte-infected grasses,” in *Biotechnology of Endophytic Fungi of Grasses*, eds Bacon C. W. White J. F. Jr. (Boca Raton, FL: CRC Press). Available online at: https://www.taylorfrancis.com/chapters/edit/10.1201/9781351070324-7/physiology-drought-tolerance-endophyte-lnfected-grasses-charles-west

[B400] West C. P. Izekor E. Oosterhuis D. M. Robbins R. T. (1988). The effect of *Acremonium coenophialum* on the growth and nematode infestation of tall fescue. *Plant Soil* 112 3–6. 10.1007/BF02181745

[B401] West C. P. Izekor E. Turner K. E. Elmi A. A. (1993). Endophyte effects on growth and persistence of tall fescue along a water-supply gradient. *Agron. J.* 85 264–270. 10.2134/agronj1993.00021962008500020019x

[B402] Western J. H. Cavett J. J. (1959). The choke disease of cocksfoot (*Dactylis glomerata*) caused by *Epichloe typhina* (Fr.) Tul. *Trans. Brit. Mycol. Soc.* 42 298–307. 10.1016/S0007-1536(56)80037-5

[B403] White J. F. Jr. (1987). Widespread distribution of endophytes in the Poaceae. *Plant Dis.* 71 340–342. 10.1094/PD-71-0340

[B404] White J. F. Jr. (1988). Endophyte–host associations in forage grasses. XI. A proposal concerning origin and evolution. *Mycologia* 80 442–446. 10.1080/00275514.1988.12025565

[B405] White J. F. Cole G. T. (1986). Endophyte-host associations in forage grasses. IV. The endophyte of *Festuca versuta*. *Mycologia* 78 102–107. 10.2307/3793384

[B406] White J. F. Sullivan R. F. Moy M. Meyer W. Cabral D. (2001). “Evolution of *Epichloë/Neotyphodium Endophytes* and other *Clavicipitalean biotrophs*,” in *Proceedings of the Symbiosis. Cellular Origin, Life in Extreme Habitats and Astrobiology)*, Vol. 4 ed. Seckbach J. (Dordrecht: Springer), 10.1007/0-306-48173-1_26

[B407] White R. H. Engelke M. C. Morton S. J. Johnson-Cicalese J. M. Ruemmele B. A. (1992). *Acremonium* endophyte effects on tall fescue drought tolerance. *Crop Sci.* 32 1392–1396. 10.2135/cropsci1992.0011183X003200060017x

[B408] Wilkinson H. H. Siegel M. R. Blankenship J. D. Mallory A. C. Bush L. P. Schardl C. L. (2000). Contribution of fungal loline alkaloids to protection from aphids in a grass–endophyte mutualism. *Mol. Plant Microbe Interact.* 13 1027–1033. 10.1094/MPMI.2000.13.10.1027 11043464

[B409] Wille P. Boller T. Kaltz O. (2002). Mixed inoculation alters infection success of strains of the endophyte *Epichloë bromicola* on its grass host Bromus erectus. *Proc. Biol. Sci.* 269 397–402. 10.1098/rspb.2001.1889 11886628 PMC1690907

[B410] Wolfe B. A. Bush M. Monfort S. L. Mumford S. L. Pessier A. Montali R. J. (1998). Abdominal lipomatosis attributed to tall fescue toxicosis in deer. *J. Amer. Vet. Med. Assoc.* 213 1783–1786. Available online at: https://pubmed.ncbi.nlm.nih.gov/9861975/9861975

[B411] Wood L. Simpson W. Johnson R. Roy S. Hoffman R. Caradus J. R. (2024). “Breeding to Improve the Wheat Phenotype While Maintaining *Epichloë endophyte* Compatibility,” in *Proceedings of the 3rd International Wheat Congress*, (Perth, WA). Available online at: https://www.iwc2024.com/wp-content/uploads/2024/10/241008_IWC-Abstract-Book-Workshop-Presentations.pdf

[B412] Xia C. Li N. Zhang Y. Li C. Zhang X. Nan Z. (2018). Role of *Epichloë endophytes* in defense responses of cool-season grasses to pathogens: A review. *Plant Dis.* 102 2061–2073. 10.1094/PDIS-05-18-0762-FE 30270751

[B413] Xu L. Li X. Han L. Li D. Song G. (2017). *Epichloë* endophyte infection improved drought and heat tolerance of tall fescue through altered antioxidant enzyme activity. *Eur. J. Hortic. Sci.* 82 90–97. 10.17660/eJHS.2017/82.2.4

[B414] Yates S. G. Fenster J. C. Bartelt R. J. (1989). Assay of tall fescue seed extracts, fractions, and alkaloids using the large milkweed bug. *J. Agric. Food Chem.* 37 354–357. 10.1021/jf00086a018

[B415] Yin L. Ren A. Wei M. Wu L. Zhou Y. Li X. et al. (2014). *Neotyphodium coenophialum*-infected tall fescue and its potential application in the phytoremediation of saline soils. *Int. J. Phytoremediat.* 16 235–246. 10.1080/15226514.2013.773275 24912220

[B416] Yoshihara T. Togiya S. Koshino H. Sakamura S. Shimanuki T. Sato T. et al. (1985). Three fungitoxic cyclopentanoid sesquiterpenes from stromata of *Epichloë typhina*. *Tetrahedron Lett.* 26 5551–5554. 10.1016/S0040-4039(01)80885-6

[B417] Young C. A. Charlton N. D. Takach J. E. Swoboda G. A. Trammell M. A. Huhman D. V. et al. (2014). Characterization of *Epichloë coenophiala* within the US: Are all tall fescue endophytes created equal? *Front. Chem.* 2:95. 10.3389/fchem.2014.00095 25408942 PMC4219521

[B418] Young C. A. Hume D. E. McCulley R. L. (2013). Forages and pastures symposium: Fungal endophytes of tall fescue and perennial ryegrass: Pasture friend or foe? *J. Animal Sci.* 91 2379–2394. 10.2527/JAS.2012-5951 23307839

[B419] Young C. A. Schardl C. L. Panaccione D. G. Florea S. Takach J. E. Charlton N. D. et al. (2015). Genetics, genomics and evolution of ergot alkaloid diversity. *Toxins* 7 1273–1302. 10.3390/toxins7041273 25875294 PMC4417967

[B420] Yue Q. Miller C. J. White J. F. Richardson M. D. (2000). Isolation and characterization of fungal inhibitors from *Epichloë festucae*. *J. Agric. Food Chem.* 48 4687–4692. 10.1021/jf990685q 11052720

[B421] Zandavar H. Afshari Babazad M. (2023). “Secondary Metabolites: Alkaloids and Flavonoids in Medicinal Plants,” in *Herbs and Spices - New Advances*, ed. Ivanišová E. (London: IntechOpen). 10.5772/intechopen.108030

[B422] Zaurov D. E. Bonos S. Murphy J. A. Richardson M. Belanger F. C. (2001). Endophyte infection can contribute to aluminium tolerance in fine fescues. *Crop Sci.* 41 1981–1984. 10.2135/cropsci2001.1981

[B423] Zbib N. Repussard C. Tardieu D. Priymenko N. Domange C. Guerre P. (2015). Toxicity of endophyte-infected ryegrass hay containing high ergovaline level in lactating ewes. *J. Animal Sci.* 93 4098–4109. 10.2527/jas.2014-8848 26440189

[B424] Zebeli Q. Lindner L. Metzler-Zebeli B. U. (2026). The dark side of grasslands: Endophyte toxicosis in horses—exposure risks, health consequences, and management. *Toxins* 18:117. 10.3390/toxins18030117 41893540 PMC13029916

[B425] Zhang W. Forester N. T. Moon C. D. Maclean P. H. Gagic M. Arojju S. K. et al. (2022). *Epichloë* seed transmission efficiency is influenced by plant defense response mechanisms. *Front. Plant Sci.* 13:1025698. 10.3389/fpls.2022.1025698 36340377 PMC9635450

[B426] Zhang X. Nan Z. Li C. Gao K. (2014). Cytotoxic effect of ergot alkaloids in *Achnatherum inebrians* infected by the *Neotyphodium gansuense* endophyte. *J. Agric. Food Chem.* 62 7419–7422. 10.1021/jf502264j 24851700

